# Management of Poisoned Patients: Implementing a Blended Toxicology Curriculum for Emergency Medicine Residents

**DOI:** 10.21980/J8C937

**Published:** 2022-04-15

**Authors:** Madeline Dwyer, Megan Stobart-Gallagher, Jared Kilpatrick, Alanna O’Connell

**Affiliations:** * Thomas Jefferson University Hospital, Department of Emergency Medicine, Philadelphia, PA; ^ Brookdale University Hospital, Department of Emergency Medicine, Brooklyn, NY

## Abstract

**Audience:**

This curriculum is appropriate for emergency medicine residents PGY 1-3 as a toxicology curriculum.

**Length of Curriculum:**

The intent is to run this curriculum over one week.

**Introduction:**

Toxicology is an important part of the emergency medicine (EM) curriculum and defined in the Council of Residency Directors (CORD) 2019 Model of Clinical Practice of Emergency Medicine as a key area of core content expected to be mastered by graduating EM seniors.^1^ Unfortunately, programs may not have time in their schedules for a dedicated toxicology curriculum, and residents may not have time to learn this important subject outside of conference didactics. Many emergency medicine programs have mandatory toxicology rotations, as many as 66% according to a 2018 study, with an additional 22% of EM programs offering an elective.^2^ At our institution, we have limited toxicology faculty available for instruction, and until now have only been able to incorporate occasional lectures into regular conference didactics, prompting our development of a new approach. Developing an asynchronous curriculum allows more dedicated time to study toxicology for our learners and allows greater flexibility for our limited toxicology faculty to teach during a synchronous component. Several asynchronous toxicology curricula have been developed previously.^3^,^4^ There have also been several novel synchronous toxicology curricular innovations for introduction during regular conference didactics.^5^,^6^ While some learners can benefit from asynchronous learning alone, it has been shown that having synchronous components in distance learning can be very important for improving learning experience and improving deep understanding rather than surface learning.^7^ Here we propose a one-week, blended asynchronous and synchronous rotation in toxicology that aims to give learners a foundation in important core toxicological concepts that they can implement on shift in the Emergency Department.

**Educational Goals:**

The goal of this curriculum is to introduce EM residents to core toxicology concepts and to reinforce toxicology principles through a multimodal approach that leads to increased confidence in the management of poisoned patients on shift.

**Educational Methods:**

The educational strategies used in this curriculum include: 1) Online asynchronous modules for each day of the week consisting of free open access medical education (FOAMed) articles, instruction on core topics, and daily quizzes. The content was created, organized, and published utilizing Articulate Rise 360 8 as a learning management system (LMS) but could easily be adapted to other LMS platforms, such as Google Classroom. The majority of educational content used to build the modules was based on *Rosen’s Emergency Medicine Concepts and Clinical Practice*. (5th editions). 2) A virtual simulation session reviewing toxicology cases with a faculty member. Cases were initially oral boards style cases but were later adapted to independent learning sessions utilizing pre-made Full-Code 9 scenarios. This could likely be adapted to other platforms such as in-person simulation for institutions without Full Code subscriptions. 3) A virtual discussion and question & answer board review session with a staff toxicologist.

**Research Methods:**

Following completion of the course, residents were encouraged to fill out a survey developed by the writer of the course designed to assess their thoughts about the course, their confidence in recognizing toxidromes as well as their comfort in the medical management of the poisoned patient. This survey was developed in-house and utilized a Likert scale and was administered on Google Forms. In an effort to promote honest feedback, residents were made aware that submissions were anonymous and email information was not collected.

**Results:**

Of the 22 participating residents (PGY1-3), 15 responded to our survey for a response rate of 68%. Overall, resident responses to the course were favorable. All participants except for one answered that they were “satisfied” or “very satisfied” with the course; the respondent who did not mark “satisfied” or “very satisfied” marked the option labeled “neutral.” Similarly, 93% (14/15) of respondents “agreed” or “strongly agreed” that they would recommend this course to a colleague, and 86% (13/15) “agreed” or “strongly agreed” that the course was a valuable use of their time.

Resident responses also indicated an increased confidence in both the recognition of toxidromes and the management of poisoned patients. The majority of respondents (9/15) indicated that their perceived confidence in recognizing toxidromes improved after completion of the course; the remainder, except for one, remarked the same level of confidence before and after completing the course. The resident who had a decline in their confidence said they were “confident” in recognizing toxidromes prior to the course and “somewhat confident” after the course. Unsurprisingly, perceived confidence in the medical management of toxicology patients improved for 87% (13/15) of respondents after having taken the course, with 2 respondents noting the same level of confidence before and after taking the course.

Lastly, multiple residents wrote in the free-response section that the toxicology rotation had been directly helpful to them when managing various toxidromes with real cases in the emergency department. For example, one response noted that they had since managed both a tricyclic antidepressant and a calcium channel blocker overdose, which they felt more comfortable with after completing the course. Another resident wrote about the experience of having a pediatric patient suffering from an ingestion of both acetaminophen and aspirin who was placed on a bicarbonate drip.

**Discussion:**

This blended synchronous and asynchronous approach to a toxicology course was a success with the residents. Based on our survey responses, the majority of the residents felt this was a valuable educational experience. Many of the residents commented on times after the course where they were directly able to apply the knowledge learned from the modules, which was also encouraging. While COVID limitations kept our synchronous aspects virtual, these were also successful with the residents. While we had initially used oral boards style cases for the simulation session, we had found that engagement with learners during these sessions was not as high as we had hoped. We subsequently switched to using the virtual simulation platform Full Code.^8^ The learners seemed to enjoy these cases much more with having more visual stimulus during the cases. It was also less work on the part of the faculty to have pre-written toxicology cases to use and lab values/imaging results a click away. However, for institutions without a Full Code subscription, oral boards style cases or in person simulation would be a worthwhile alternative. Additionally, video conference sessions with our toxicology faculty members were helpful for the residents to go over the information they had learned in a question-and-answer format.

Regarding the synchronous aspect of the course, having written the modules ahead of time, it was very easy to upload into an LMS. We particularly found Articulate Rise^7^ to be helpful as an LMS, especially with integrating some interactive elements into each module. However, this could easily be adapted into any LMS your institution prefers, or even into slideshow software.

**Topics:**

General approach to poisoned patient, gastric decontamination, dialysis in toxicology, acetaminophen overdose (od), salicylate od, carbon monoxide poisoning, pediatric toxicology considerations, alcohol withdrawal, toxic alcohols, beta blocker od, calcium channel blocker od, tca od, serotonin syndrome, opiate od, body packers vs stuffers, marijuana, synthetic cannabinoids, gamma hydroxybutyric acid (ghb) od; cocaine toxicity, inhalant abuse, spider envenomations, snake envenomations, marine envenomations, mushroom toxicities, organophosphate poisoning.

## User Guide

**Table:** 

List of Resources:	
Abstract	1
User Guide	5
Didactics and Hands on Curriculum Chart	11
Synchronous Toxicology Curriculum Question and Answers PDF	14
Monday Quiz	15
Tuesday Quiz	17
Wednesday Quiz	18
Thursday Quiz	21
Friday Quiz	23
Monday Quiz Answers	24
Tuesday Quiz Answers	26
Wednesday Quiz Answers	27
Thursday Quiz Answers	30
Friday Quiz Answers	32

**Learner Audience:**

Interns, junior residents, senior residents

**Length of Curriculum:**

It is currently executed as a required one-week core rotation for PGY1 residents and an optional one-week elective for PGY2/3 residents.

**Topics:**

General approach to poisoned patient, gastric decontamination, dialysis in toxicology, acetaminophen overdose (od), salicylate od, carbon monoxide poisoning, pediatric toxicology considerations, alcohol withdrawal, toxic alcohols, beta blocker od, calcium channel blocker od, tca od, serotonin syndrome, opiate od, body packers vs stuffers, marijuana, synthetic cannabinoids, gamma hydroxybutyric acid (ghb) od; cocaine toxicity, inhalant abuse, spider envenomations, snake envenomations, marine envenomations, mushroom toxicities, organophosphate poisoning.

**Objectives:**

Module objectives separated by each day of the rotation.

1. Day 1 Objectives: General Toxicology Overview and Common Tox Scenarios
  - Review the basic approach to the intoxicated patient
  - Differentiate the types of gastric decontamination
  - Review the use of dialysis in toxicology
  - Understand the pathophysiology and treatment of the acetaminophen overdose
  - Understand the pathophysiology and treatment of the salicylate overdose
  - Recognize the symptoms and treatment of carbon monoxide poisoning
2. Day 2 Objectives: Toxic Alcohols, Alcohol Withdrawal, Pediatric Toxicology
  - Attend Poison Control Center Grand Rounds and/or review “one pill kills” in pediatric toxicology.
  - Contrast the different treatments for alcohol withdrawal
  - Compare the presentations of the different toxic alcohols
3. Day 3 Objectives: Major Resuscitations, Simulations
  - Manage a case of beta blocker overdose
  - Contrast what findings would be in a calcium channel blocker overdose
  - Manage a TCA overdose
  - Recognize Serotonin Syndrome
4. Day 4 Objectives: Drugs of Abuse
  - Identify opiate overdoses and treatment
  - Review the differences between body packing and stuffing
  - Contrast marijuana and synthetic marijuana overdose presentations
  - Understand GHB overdose and treatment
  - Recognize cocaine toxicity and treatment
  - Understand the dangers of inhalant abuse
5. Day 5 Objectives: Envenomations, Plant Toxicology, Organophosphates
  - Differentiate brown recluse from black widow envenomations
  - Identify common snakes that cause significant envenomations
  - Classify commonly tested mushroom/plant toxicities
  - Understand the toxidrome and treatment of organophosphate poisoning

### Brief introduction

Toxicology is an important part of the emergency medicine (EM) curriculum and defined in the Council of Residency Directors (CORD) 2019 Model of Clinical Practice of Emergency Medicine as a key area of core content expected to be mastered by graduating EM seniors.^1^ This is an area frequently assessed on both the In-Training examination (ITE), as well as the Emergency Medicine Written and Oral Board Exams. We frequently see patients with toxicological emergencies in practice as well which necessitates a strong background in toxicology to best care for these patients.

### Problem identification, general and targeted needs assessment

Many EM residency programs may not have time in their schedules for a dedicated toxicology curriculum so residents may not have dedicated time to learn this important subject outside of conference didactics. Many emergency medicine programs have mandatory toxicology rotations, as many as 66% according to a 2018 study with an additional 22% offering an elective.^2^ At our particular institution, we have limited toxicology faculty available for instruction and until now have only been able to incorporate sporadic lectures into regular conference didactics, prompting our development of a new approach. Developing an asynchronous curriculum would allow more dedicated time to study toxicology for our learners, and allow greater flexibility for our limited toxicology faculty to teach during a synchronous component. Several asynchronous toxicology curricula have been developed previously.^2^,^3^ There also have been several novel synchronous toxicology curricular innovations for introduction during regular conference didactics.^4^,^5^ While some learners can benefit from asynchronous learning alone, it has been shown that having synchronous components in distance learning can be very important for improving learning experience and improving deep understanding rather than surface learning.^6^

Here we propose a one-week, blended asynchronous and synchronous rotation in toxicology that will give learners a foundation in important core toxicology concepts emergency medicine residents can implement on shift in the Emergency Department. This was a required PGY 1 rotation, but was available to residents of other years as well as an elective. Using a cognitivist framework for this approach, residents spend time on the asynchronous modules, and then have the ability to link the concepts learned online to scenarios proposed during synchronous activities with faculty. We hope this will lead to improved patient care since residents will have a deeper understanding of how to manage the poisoned patient.

### Goals of the curriculum

To introduce EM residents to core toxicology concepts, and to reinforce toxicology principles through a multimodal approach that leads to increased confidence in the management of poisoned patients on shift.

### Objectives of the curriculum

Module objectives separated by each day of the rotation

1. Day 1 Objectives: General Toxicology Overview and Common Tox Scenarios
  - Review the basic approach to the intoxicated patient
  - Differentiate the types of gastric decontamination
  - Review the use of dialysis in toxicology
  - Understand the pathophysiology and treatment of the acetaminophen overdose
  - Understand the pathophysiology and treatment of the salicylate overdose
  - Recognize the symptoms and treatment of carbon monoxide poisoning
2. Day 2 Objectives: Toxic Alcohols, Alcohol Withdrawal, Pediatric Toxicology
  - Attend Poison Control Center Grand Rounds and/or review “one pill kills” in pediatric toxicology.
  - Contrast the different treatments for alcohol withdrawal
  - Compare the presentations of the different toxic alcohols
3. Day 3 Objectives: Major Resuscitations, Simulations
  - Manage a case of beta blocker overdose
  - Contrast what findings would be in a calcium channel blocker overdose
  - Manage a TCA overdose
  - Recognize Serotonin Syndrome
4. Day 4 Objectives: Drugs of Abuse
  - Identify opiate overdoses and treatment
  - Review the differences between body packing and stuffing
  - Contrast marijuana and synthetic marijuana overdose presentations
  - Understand GHB overdose and treatment
  - Recognize cocaine toxicity and treatment
  - Understand the dangers of inhalant abuse
5. Day 5 Objectives: Envenomations, Plant Toxicology, Organophosphates
  - Differentiate brown recluse from black widow envenomations
  - Identify common snakes that cause significant envenomations
  - Classify commonly tested mushroom/plant toxicities
  - Understand the toxidrome and treatment of organophosphate poisoning

### Educational Strategies

(See curriculum chart) Please refer to the curriculum chart of linked objectives and educational strategies.

### Results and tips for successful implementation

In total, 22 residents completed the previously described toxicology curriculum between July 2020–July 2021. Due to social distancing guidelines of the coronavirus pandemic, the entire curriculum was administered virtually; however, the synchronous portions of this curriculum could likely be easily adapted to in-person learning as well. Satisfaction with the course as well as perceived confidence in medical management of toxicology patients was assessed using a survey distributed to residents after completion of the course.

Of the 22 participating residents (PGY1-3), 15 responded to our survey for a response rate of 68%. Overall, resident responses to the course were favorable. All participants except for one answered that they were “satisfied” or “very satisfied” with the course; the respondent who did not mark “satisfied” or “very satisfied,” marked the option labeled “neutral.” Similarly, 93% (14/15) of respondents “agreed” or “strongly agreed” that they would recommend this course to a colleague, and 86% (13/15) “agreed” or “strongly agreed” that the course was a valuable use of their time (table 1).

Resident responses also indicated an increased confidence in both the recognition of toxidromes and the management of poisoned patients. The majority of respondents (9/15) indicated that their perceived confidence in recognizing toxidromes improved after completion of the course; the remainder, except for one, remarked the same level of confidence before and after completing the course. The resident who had a decline in confidence was “confident” in recognizing toxidromes prior to the course and “somewhat confident” after the course. Unsurprisingly, perceived confidence in the medical management of toxicology patients improved for 87% (13/15) of respondents after having taken the course, with 2 respondents noting the same level of confidence before and after taking the course.

Lastly, multiple residents wrote in the free-response section that the toxicology rotation had been directly helpful to them when managing various toxidromes with real cases in the emergency department. For example, one response noted that they had since managed both a tricyclic antidepressant and a calcium channel blocker overdose, which they felt more comfortable with after completing the course. Another resident wrote about the experience of having a pediatric patient suffering from an ingestion of both acetaminophen and aspirin who was placed on a bicarbonate drip.

This blended synchronous and asynchronous approach to a toxicology course was a success with the residents. Based on our survey responses, the majority of the residents felt this was a valuable educational experience. Many of the residents commented on times after the course where they were directly able to apply the knowledge learned from the modules, which was also encouraging. While COVID limitations kept our synchronous aspects virtual, these were also successful with the residents. While we had initially used oral boards style cases for the simulation session, we had found that engagement with learners during these sessions was not as high as we had hoped. We subsequently switched to using the virtual simulation platform Full Code.^8^ The learners seemed to enjoy these cases much more with having more visual stimulus during the cases. It was also less work on the part of the faculty to have prewritten toxicology cases to use and lab values/imaging results a click away. However, for institutions without a Full Code subscription, oral boards style cases or in-person simulation would be a worthwhile alternative. Additionally, video conference sessions with our toxicology faculty members were helpful for the residents to go over the information they had learned in a question-and-answer format.

Regarding the synchronous aspect of the course, having written the modules ahead of time, it was very easy to upload into an LMS. We particularly found Articulate Rise^7^ to be helpful as an LMS, especially with integrating some interactive elements into each module. However, this could easily be adapted into any LMS your institution prefers, or even into slideshow software.

### Facilitator Instructions

What worked best at our institution was to delegate a faculty member/fellow to be available during the week to answer questions as they came up for the residents as they were reviewing the modules. The same facilitator would be available on Wednesdays to either run Full Code simulations, or if your institution does not have access, “oral board” style cases or full simulations. Some alternatives have been listed below. On Fridays there was a Q and A session with our staff toxicologist, and we have made his slides available for your use should you choose to use them. They are also linked below.

### Evaluation and Feedback

Initially simulations were done virtually on a video conferencing platform as an oral boards style case. However, residents had commented that this was not as engaging as an in-person simulation case would be. We transitioned to using Full-Code,^8^ an online simulation platform to help increase engagement. This was met with much more positive feedback.

### Associated Content

Link to our module on Rise 360:

- https://rise.articulate.com/share/UjRQ7D_jh2tSWnzDZ2n8397SvUPCgUZm

List of Full Code Cases Used:

- #32: Calcium channel blocker overdose
- #21: Tricyclic antidepressant overdose
- #25: Sulfonylurea overdose
- #36: Snake envenomation
- #113: Acetaminophen overdose
- #81: Opioid overdose
- #78: Opioid overdose
- #64: Caustic ingestion
- #10: Salicylate overdose
- #5: Organophosphate exposure
- #3: Alcohol withdrawal

Link to American College of Emergency Physicians (ACEP) sample toxicology cases for institutions without Full Code access:

- https://www.acep.org/tox/

Appendices:

- Synchronous Toxicology Curriculum Question and Answers PDF
- Monday Quiz
- Tuesday Quiz
- Wednesday Quiz
- Thursday Quiz
- Friday Quiz
- Monday Quiz Answers
- Tuesday Quiz Answers
- Wednesday Quiz Answers
- Thursday Quiz Answers
- Friday Quiz Answers

### Further Readings

#### General Tox/Approach

1. Meehan TJ. Approach to the Poisoned Patient. In: Walls R, Hockberger R, Gausche-Hill M, eds. *Rosen's Emergency Medicine Concepts and Clinical Practice*. 9th ed. Philadelphia, PA: Elsevier; 2018:1813–1822.
2. Nickson C. Gastric lavage. LITFL. November 3, 2020. Retrieved October 21, 2021 from: https://litfl.com/gastric-lavage/

#### Acetaminophen

1. Hendrickson RG, McKeown NJ. Acetaminophen. In: Walls R, Hockberger R, Gausche-Hill M, eds. *Rosen's Emergency Medicine Concepts and Clinical Practice*. 9th ed. Philadelphia, PA: Elsevier; 2018:1852–1857.
2. Abdrabbo M, Santos C. TOXCard: Acetaminophen Toxicity and Management. emDOCs.net. March 29, 2018. Retrieved October 21, 2021, from: http://www.emdocs.net/toxcard-acetaminophen-toxicity-and-management/
3. Farkas J. Acetaminophen Toxicity - EMCrit Project. October 8, 2021. Retrieved October 21, 2021, from: https://emcrit.org/ibcc/acetaminophen/

#### Salicylates

1. Hendrickson RG, McKeown NJ. Salicylates. In: Walls R, Hockberger R, Gausche-Hill M, eds. *Rosen's Emergency Medicine Concepts and Clinical Practice*. 9th ed. Philadelphia, PA: Elsevier; 2018:1852–57.
2. Farkas J. Salicylate Intoxication. EMCrit Project. October 1, 2021. Retrieved October 21, 2021, from: https://emcrit.org/ibcc/salicylates/
3. Swaminathan A. Salicylate Toxicity. REBEL EM. Emergency Medicine Blog. May 17, 2018. Retrieved October 21, 2021, from: https://rebelem.com/salicylate-toxicity/

#### Carbon Monoxide

1. Nelson LS, Hoffman RS. Carbon Monoxide. In: Walls R, Hockberger R, Gausche-Hill M, eds. *Rosen's Emergency Medicine Concepts and Clinical Practice*. 9th ed. Philadelphia, PA: Elsevier; 2018;1931–1933.
2. Repplinger D. Carbon Monoxide Poisoning: Common Questions and Dilemmas. (n.d.). ALiEM. Retrieved October 21, 2021, from: https://www.aliem.com/dive-dive/
3. Simon E. Carbon Monoxide Poisoning. emDOCs.net. May 20^th^, 2017. Retrieved October 21, 2021, from: http://www.emdocs.net/carbon-monoxide-poisoning/
4. Nickson, C. Carbon Monoxide Poisoning. LITFL. November 3, 2020. Retrieved October 21, 2021, from: https://litfl.com/carbon-monoxide-poisoning/

#### Alcohol Withdrawal

1. Alcohol Withdrawal: Featuring Drunkbrain! One Minute Medical School. YouTube. May 26, 2013. Retrieved October 21, 2021, from: https://www.youtube.com/watch?v=WhOvN5XoIgIToxicAlcohols
2. Finnell JT. Alcohol-Related Disease. In: Walls R, Hockberger R, Gausche-Hill M, eds. *Rosen's Emergency Medicine Concepts and Clinical Practice*. 9th ed. 2018. Philadelphia, PA: Elsevier;1838–1849.
3. Spyres M. Episode 129.0 – Toxic Alcohols. – Core EM. January 22, 2018. Retrieved October 21, 2021, from https://coreem.net/podcast/episode-129-0-toxic-alcohols/

#### Beta Blocker and Calcium Channel Blocker Overdose

1. Cole J, Spyres M. Tox and Hound - One Therapy to Rule Them All? Sugar vs Squeeze in Cardiotoxic Poisoning. February 18, 2020. Retrieved October 21, 2021, from: https://emcrit.org/toxhound/hdi-vs-pressor/
2. Tox Cards: Intralipid Rescue. emDOCs.net. Retrieved October 21, 2021, from: http://www.emdocs.net/intralipid-rescue/

#### TCA Overdose

1. Misch M. Crit Cases 1:| Massive TCA Overdose. | Emergency Medicine Cases. January 2016. Retrieved October 21, 2021, from https://emergencymedicinecases.com/critcases-massive-tca-overdose/

#### Serotonin Syndrome and Toxin Induced Hyperthermic Disorders

1. Farkas J. Serotonin syndrome - EMCrit Project. October 1, 2021. Retrieved October 21, 2021, from https://emcrit.org/ibcc/serotonin/
2. Traficante D, Kashani J. Tips for Diagnosing, Treating Toxin-Induced Hyperthermic Disorders. ACEP Now. December 14, 2016. Retrieved October 21, 2021, from: https://www.acepnow.com/article/tipsdiagnosingtreatingmbertoxin-induced-hyperthermic-disorders/

#### Opiates

1. Nikolaides JT, Thompson TM. Opioids. In: Walls R, Hockberger R, Gausche-Hill M, eds. *Rosen's Emergency Medicine Concepts and Clinical Practice*. 9th ed. Philadelphia, PA: Elsevier; 2018:1943–1946.
2. Cole J. Great! Naloxone worked! Now what? Tox and Hound. November 4, 2019. Retrieved October 21, 2021, from: https://emcrit.org/toxhound/naloxone-now-what
3. Long N. Opioid Toxicity. LITFL. November 3, 2020. Retrieved October 21, 2021, from: https://litfl.com/opioid-toxicity/
4. Ramzy M. The HOUR Trial: Clinical Decision Rule for Opioid Overdose Patients in the Emergency Department. REBEL EM. January 21, 2019. Retrieved October 21, 2021, from: https://rebelem.com/hour-trial/

#### Synthetic Cannabinoids

1. Iwaniki JL. Cannabinoids. In: Walls R, Hockberger R, Gausche-Hill M, eds. *Rosen's Emergency Medicine Concepts and Clinical Practice*. 9th ed. Philadelphia, PA: Elsevier; 2018:1907–1911.
2. Oliver J. EM@3AM: Synthetic Cannabinoid Intoxication. emDOCs.net. January 27, 2018. Retrieved October 21, 2021, from: http://www.emdocs.net/em3am-synthetic-cannabinoid-intoxication/
3. Roberts, J. R. InFocus-Synthetic Cannabinoids. *Emergency Medicine News*; 2015: *37*(8), 12–14.

#### GHB

1. Gussow L, Carson A. Sedative Hypnotics. In: Walls R, Hockberger R, Gausche-Hill M, eds. *Rosen's Emergency Medicine Concepts and Clinical Practice*. 9th ed. Philadelphia, PA: Elsevier; 2018:1974–1982.
2. Long N. GHB toxicity. LITFL. March 6, 2019. Retrieved October 21, 2021, from: http://litfl.com/ghb-toxicity/
3. Garrett P, Ofoma M, Bral DO. GHB: A Forgotten Foe Rises. EMRA. December 16, 2018. Retrieved October 21, 2021, from: https://www.emra.org/emresident/article/ghb/

#### Cocaine

1. Rama BR, Hoffman RS, Erickson TB. Cocaine. In: Walls R, Hockberger R, Gausche-Hill M, eds. *Rosen's Emergency Medicine Concepts and Clinical Practice*. 9th ed. 2018. Philadelphia, PA: Elsevier;1895–1903.
2. Simon E. Cocaine Toxicity. emDOCs.net. June 18, 2017. Retrieved October 21, 2021, from http://www.emdocs.net/em3am-cocaine-toxicity/
3. Nickson C. Cocaine Toxicity. LITFL. November 3, 2020. Retrieved October 21, 2021, from: https://litfl.com/cocaine-toxicity-ccc/
4. Nickson C. Cocaine-related Chest Pain. LITFL. November 3, 2020. Retrieved October 21, 2021, from: https://litfl.com/cocaine-related-chest-pain/

#### Huffing

1. Wang GS, Buchanan JA. Hydrocarbons. In: Walls R, Hockberger R, Gausche-Hill M, eds. *Rosen's Emergency Medicine Concepts and Clinical Practice*. 9th ed. Philadelphia, PA: Elsevier; 2018:1921.
2. Fox FM. Huffing Hydrocarbons: Inhalant Abuse. Pediatric EM Morsels. September 7, 2018. Retrieved October 21, 2021, from: https://pedemmorsels.com/huffing-hydrocarbons-inhalant-abuse
3. Inhalant Abuse. Emergency Physicians Monthly. Retrieved October 21, 2021, from: https://epmonthly.com/article/inhalant-abuse/

#### Envenomations

1. Otten EJ. Venomous Animal Injuries. In: Walls R, Hockberger R, Gausche-Hill M, eds. *Rosen's Emergency Medicine Concepts and Clinical Practice*. 9th ed. Philadelphia, PA: Elsevier; 2018: 698–714. emDOCs.net
2. Pirrote A, Wagner J, Pirrote M. A Case of Severe Brown Recluse Envenomation. emDOCs.net September 22, 20116. Retrieved October 21, 2021, from: http://www.emdocs.net/case-severe-brown-recluse-envenomation/
3. Venomous Creatures: Black Widow Envenomation (Latrodectism). Kings County Downstate Emergency Medicine. June 3, 2019. Retrieved October 21 2021 from:http://blog.clinicalmonster.com/2019/06/03/venomous-creatures-widows-latrodectism/
4. Riester J, Shenvi C. Envenomations: Initial Management of Common U.S. Snakebites. June 23, 2017. Retrieved October 21, 2021, fromL https://www.aliem.com/envenomations-snakebites/

#### Mushroom Toxicity

1. Lim CS, Aks SE. Plants, Mushrooms, and Herbal Medications. In: Walls R, Hockberger R, Gausche-Hill M, eds. *Rosen's Emergency Medicine Concepts and Clinical Practice*. 9th ed. Philadelphia, PA: Elsevier; 2018:1957.
2. Nickson C. Mushroom Toxicity. LITFL. November 3, 2020. October 21, 2021, from: https://litfl.com/mushroom-toxicity/

#### Marine Envenomations

1. Cheema N. 5-Step Approach to Marine Envenomations. EMRA. April 7, 2016. Retrieved October 21, 2021, from: https://www.emra.org/emresident/article/5-step-approach-to-marine-envenomations/
2. Spyres M. Tox and Hound - Some Like It Hot. January 14, 2019. Retrieved October 21, 2021, from https://emcrit.org/toxhound/likeithot/

#### Organophosphates

1. Welker K, Thompson TM. Pesticides. In: Walls R, Hockberger R, Gausche-Hill M, eds. *Rosen's Emergency Medicine Concepts and Clinical Practice*. 9th ed. 2018. Philadelphia, PA: Elsevier;1947–1949.
2. Sahi N, Santos C. TOXCard: Nerve Agents. emDOCs.net. July 18, 2018. Retrieved October 21, 2021, from: http://www.emdocs.net/toxcard-nerve-agents/

## Supplementary Information



## Figures and Tables

**Table:** 

Topic	Recommended Educational Strategy	Educational Content	Objectives	Learners	Timing, Resources Needed (Space, Instructors, Equipment, Citations of JETem pubs or other literature)	Recommended Assessment, Milestones Addressed
Day 1: General Toxicology Overview and Common Toxicology Scenarios	Asynchronous learning modules on an online learning management system	Review the basic approach to the intoxicated patient Differentiate the types of gastric decontamination Review the use of dialysis in toxicology Understand the pathophysiology and treatment of acetaminophen overdose Understand the pathophysiology and treatment of salicylate overdose Recognize the symptoms and treatment of carbon monoxide poisoning	-The learner will understand the indications for dialysis and gastric decontamination in toxicology scenarios -The learner will review the pathophysiology, management, and treatment options for acetaminophen, salicylate, and carbon monoxide poisonings -The learner will develop a framework for a basic approach to the management of poisoned patients	PGY-1 to PGY-3 resident physicians	Time to complete module: 1 hour Resources needed: Access to your preferred learning management system	Assessment: Post-module quiz; PC1, PC4, PC5, PC9
Day 2: Toxic Alcohols, Alcohol Withdrawal, Pediatric Toxicology	Asynchronous learning modules on an online learning management system	Attend Poison Control Center Grand Rounds and/or review “one pill kills” in pediatric toxicology. Review alcohol withdrawal treatments Compare the presentations of the different toxic alcohols	-The learner will be able to differentiate between the clinical presentations caused by toxic alcohols -The learner will review the most dangerous pediatric ingestions of common medications -The learner will contrast different treatments for alcohol withdrawal	PGY-1 to PGY-3 resident physicians	Time to complete module: 1 hour Resources needed: Access to your preferred learning management system Access to Poison Center Grand Rounds (if available)	Assessment: Post-module quiz; PC1, PC4, PC5
Day 3: Major Resuscitations, Simulations	Asynchronous learning modules on an online learning management system Online clinical simulation software, such as FullCode. Alternatively, a live, in-person simulation lab could be used if available	Complete a simulation case of beta blocker overdose Contrast what findings would be seen in a calcium channel blocker overdose Complete a simulation case on TCA overdose Recognize the signs and symptoms of serotonin syndrome	-The learner will identify beta blocker overdose and employ appropriate treatment modalities in a synchronous simulation session -The learner will be able to differentiate a calcium channel overdose from a beta blocker overdose -The learner will recognize tricyclic antidepressant overdose and employ appropriate treatment modalities in a synchronous simulation session -The learner will review serotonin syndrome and its management	PGY-1 to PGY-3 resident physicians	Time to complete module: 1 hour Resources needed: Access to your preferred learning management system Access to an online simulation system or simulation lab A faculty member to run the simulation session for 30 minutes to 1 hour per group	Assessment: Post-module quiz and real time feedback regarding the simulation; PC1, PC4, PC5
Day 4: Drugs of Abuse	Asynchronous learning modules on an online learning management system	Identify opiate overdoses and treatment Review the differences between body packing and stuffing Contrast marijuana and synthetic marijuana overdose presentations Understand gamma-hydroxybutyrate (GHB) overdose and treatment Recognize cocaine toxicity and treatment Understand the dangers of inhalant abuse	-The learner will be able to differentiate between the toxidromes of various recreational drugs and learn the management of these intoxications -The learner will be able to identify the dangers associated with body packing and stuffing	PGY-1 to PGY-3 resident physicians	Time to complete module: 1 hour Resources needed: Access to your preferred learning management system	Assessment: Post-module quiz; PC1, PC4, PC5
Day 5: Envenomations, Plant Toxicology, Organophosphates	Asynchronous learning modules on an online learning management system Synchronous question and answer session with toxicologist	Differentiate brown recluse from black widow envenomations Identify common snakes that cause significant envenomations Classify commonly tested mushroom/plant toxicities Understand the toxidrome and treatment of organophosphate poisoning Review commonly tested toxicology questions and develop a systematic approach for toxicology questions	-The learner will distinguish between envenomations and management differences of the black widow and brown recluse spider bites -The learner will identify snakes that cause clinically significant envenomations -The learner will review mushroom and plant toxicities -The learner will identify the organophosphate toxicity and become familiar with treatment options	PGY-1 to PGY-3 resident physicians	Time to complete module: 1 hour Resources needed: Access to your preferred learning management system; approximately 1 hour of time per small group from a toxicologist (or toxicology interested faculty member) to review toxicology questions	Assessment: Post module quiz and real time feedback during question-and-answer session from a toxicologist; PC1, PC4, PC5

**Figure:**
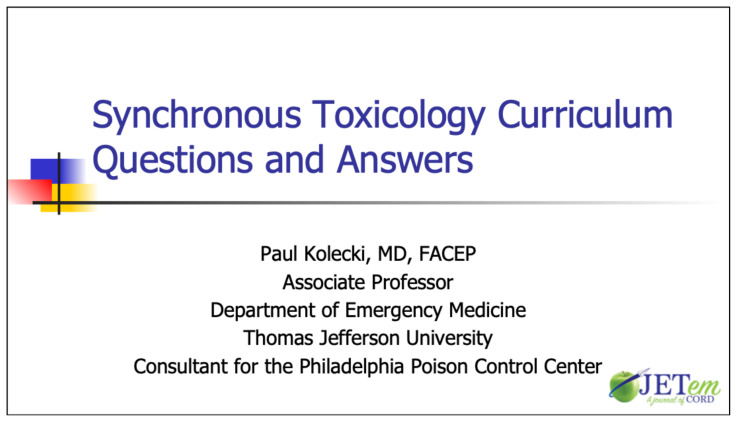
Please see associated PowerPoint file

**Figure:**
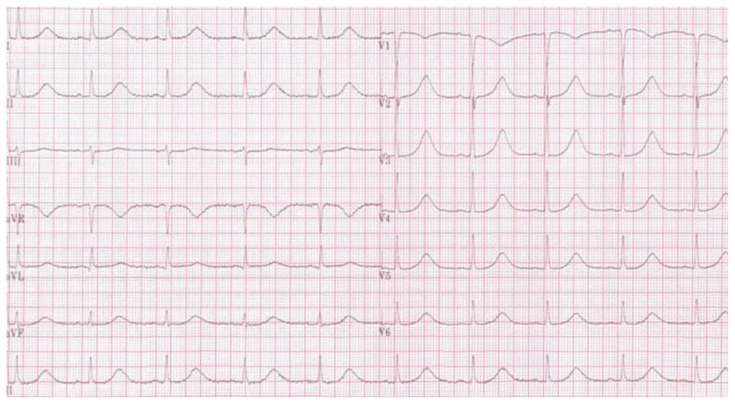
Source: CardioNetworks. Long QT Syndrome. Wikipedia. https://en.wikipedia.org/wiki/Long_QT_syndrome Creative Commons Attribution-ShareAlike License 3.0

**Figure:**
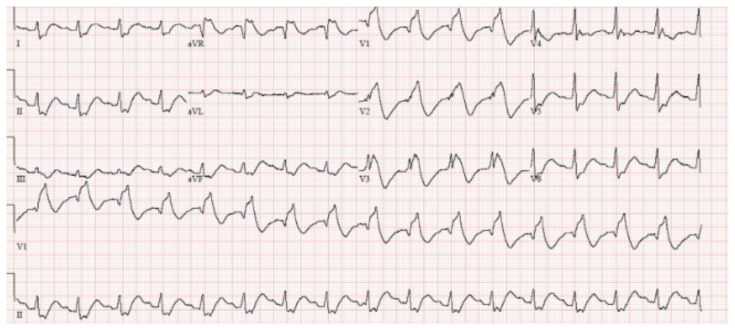
Source: Authors’ Own Image

**Figure:**
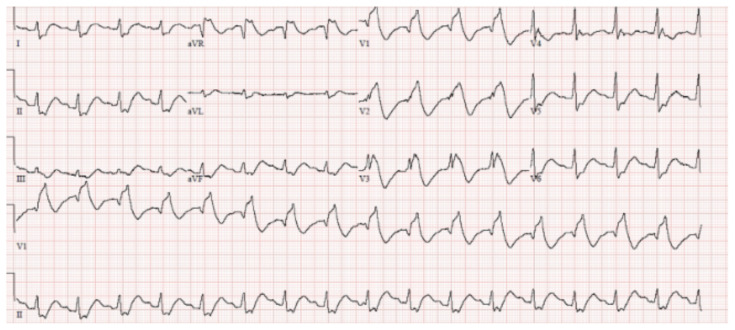
Source: Authors’ Own Image

**Figure:**
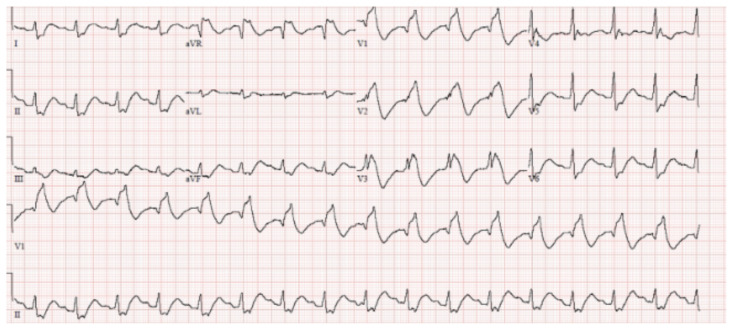
Source: Authors’ Own Image

**Figure:**
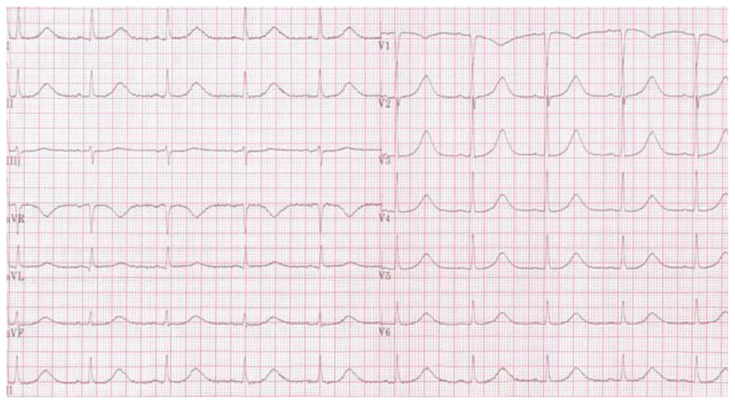
Source: CardioNetworks. Long QT Syndrome. Wikipedia. https://en.wikipedia.org/wiki/Long_QT_syndrome Creative Commons Attribution-ShareAlike License 3.0

**Figure:**
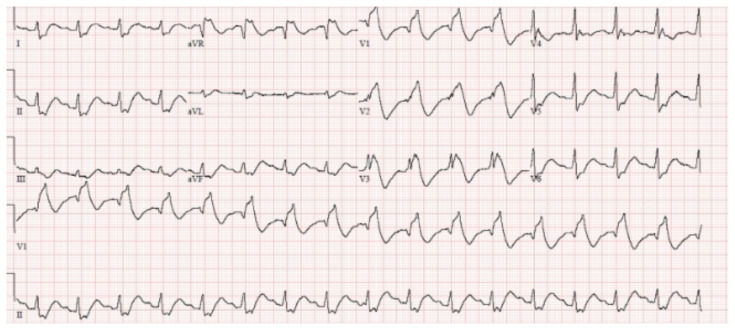
Source: Authors’ Own Image

**Figure:**
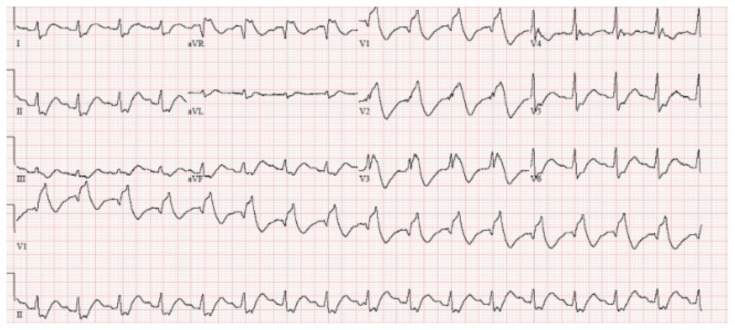
Source: Authors’ Own Image

**Figure:**
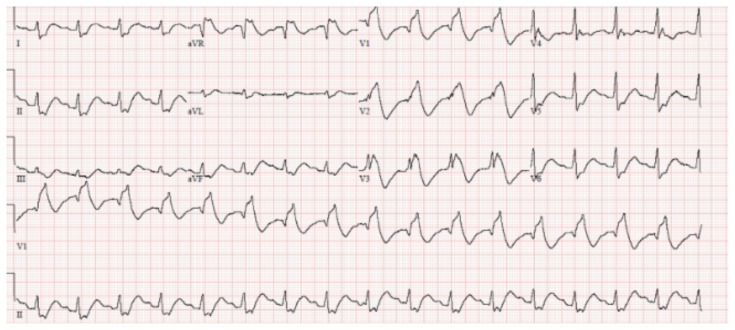
Source: Authors’ Own Image

**Table 1:** Survey Results

What Year in Residency Are You?	Was this a required rotation or an elective for you?	If this was an elective for you, did you feel that this was a valuable use of your elective time?	For all rotators, were you satisfied with your knowledge gained from this week?	Would you recommend this rotation to your co- residents?	How confident were you at recognizing various toxidromes prior to completion of this course?	How confident are you at recognizing various toxidromes now that you have completed this course?	How confident were you in the medical management of toxicology patients prior to completion of this course?	How confident are you in the medical management of toxicology patients now that you have completed this course?
PGY1	Required	Agree	Satisfied	Agree	Not confident	Confident	Not confident	Somewhat confident
PGY1	Required	Agree	Satisfied	Agree	Not confident	Somewhat confident	Not confident	Somewhat confident
PGY1	Required	Neutral	Very Satisfied	Strongly Agree	Confident	Somewhat confident	Not confident	Somewhat confident
PGY1	Required	Strongly Agree	Very Satisfied	Strongly Agree	Not confident	Very confident	Not confident	Very confident
PGY1	Required	Neutral	Neutral	Neutral	Somewhat confident	Somewhat confident	Somewhat confident	Somewhat confident
PGY1	Required		Very Satisfied	Agree	Somewhat confident	Confident	Not confident	Somewhat confident
PGY1	Required		Very Satisfied	Agree	Somewhat confident	Confident	Not confident	Somewhat confident
PGY1	Required		Satisfied	Agree	Somewhat confident	Somewhat confident	Not confident	Somewhat confident
PGY2	Elective	Strongly Agree	Very Satisfied	Strongly Agree	Somewhat confident	Very confident	Somewhat confident	Very confident
PGY2	Elective	Strongly Agree	Very Satisfied	Strongly Agree	Very confident	Very confident	Very confident	Very confident
PGY2	Elective	Strongly Agree	Very Satisfied	Strongly Agree	Somewhat confident	Confident	Somewhat confident	Confident
PGY2	Elective	Agree	Satisfied	Strongly Agree	Somewhat confident	Confident	Somewhat confident	Confident
PGY3	Elective	Strongly Agree	Satisfied	Strongly Agree	Confident	Very confident	Confident	Very confident
PGY3	Elective	Strongly Agree	Very Satisfied	Strongly Agree	Very confident	Very confident	Somewhat confident	Very confident
PGY3	Elective	Strongly Agree	Very Satisfied	Strongly Agree	Somewhat confident	Confident	Somewhat confident	Confident

## DIDACTICS AND HANDS-ON CURRICULUM

### Monday Quiz

1. You have a patient presenting after overdosing on lithium. She states she took 100 pills 30 minutes ago. Your attending thinks you should give activated charcoal. Why is this a bad idea?
  - She is not in the time frame activated charcoal would be effective.
  - Activated Charcoal will not be effective against Lithium.
2. What is the toxic metabolite of acetaminophen?
  - Glutathione
  - NAPQI
  - NAC
  - Acetaminophen Reductase
3. You have a patient who presents after a Tylenol ingestion which took place about 4 hours ago. The patient’s Tylenol level is 250micrograms/ml. What tool can you use to determine if they need NAC?
  - Laboratory value cut off
  - Nomogram
4. For the same patient: "You have a patient who presents after a Tylenol ingestion which took place about 4 hours ago. The patient’s Tylenol level is 250micrograms/ml." Based on the nomogram should you give NAC?
  - Yes
  - No
5. What is the initial dose of NAC?
  - 250 mg/kg
  - 150 mg/kg
  - 50 mg/kg
  - 25 mg/kg
6. You have a patient presenting with ringing in her ears. She has been doing a lot of strenuous workouts and applying tiger balm liberally to soothe her aching muscles. What salicylate level would you expect her blood work to show?
  - >10 mg/DL
  - >20 mg/DL
  - >30 mg/DL
7. What electrolyte do we especially care about replacing in salicylate overdose?
  - Magnesium
  - Potassium
  - Calcium
  - You have a patient presenting to your ER after a house fire. On primary survey their ABC’s are intact, but you notice they are responding inappropriately to questions. Glasgow Coma Scale is 14 with a point taken away for that. While your colleague is performing the secondary survey you are placing orders in the computer. What lab tests should you consider ordering given the patient’s altered mental status? (free response)
8. You have a pregnant patient who is under investigation for possible CO exposure. At what level should you consult your local hyperbaric center for possible dive?
  - >5%
  - >8%
  - >15%

### Tuesday Quiz

1. You are seeing a patient for anxiety. Patient states he has been feeling very anxious since this morning. He is tremulous on evaluation and appears diaphoretic. His vitals are notable for being tachycardic and hypertensive. You suspect alcohol withdrawal and want to start treatment. How do most treatments for alcohol withdrawal work?
  - GABA Agonist
  - GABA Antagonist
2. A patient presents with altered mental status. You obtain a head CT which is normal and basic labs are unremarkable except for an anion gap of 32. What do you suspect could be causing his altered mental status? (select all that apply)
  - Methanol
  - Ethanol
  - Cocaine
  - Ethylene glycol
3. For the same patient, the wife finally arrives and you obtain history that he was found in the garage next to an empty bottle of antifreeze. What treatment should you start immediately? (free response)
4. It turns out the hospital has only enough fomepizole for one dose; what other treatment modalities could you consider? (free response)

### Wednesday Quiz

1. What is the main concerning abnormal finding on this EKG?
  - STEMI
  - Hyperkalemia
  - Long QT
  - Widened QRS
2. What rhythm can a prolonged QT interval lead to?
  - Atrial fibrillation
  - Bidirectional tachycardia
  - Torsades de points
  - PEA
  - You have a patient presenting with fever and tachycardia after overdosing on their citalopram (an SSRI). In addition to supportive care (cooling, benzodiazepines, etc) what medication could you consider administering? (free response)
3. You have a patient presenting with hyperthermia and altered mental status. You discuss the case with the attending and express your concern for serotonin syndrome since you are now an expert in this. The attending wants to know why you think it’s not malignant hyperthermia or neuroleptic malignant syndrome. What key physical exam finding will help you differentiate these?
  - Wet Skin/Diaphoresis
  - Findings of empty pill bottles in pockets
  - Ankle clonus on neuro exam
  - Pupils appear normal
4. What is the main concerning abnormal finding on this EKG?
  - STEMI
  - Hyperkalemia
  - Prolonged QT
  - Widened QRS
5. What’s the toxic dose of lidocaine without epinephrine added?
  - 4 mg/kg
  - 5 mg/kg
  - 6 mg/kg
  - 7 mg/kg
6. You have a patient who arrives to the ER for suicidal ideation. After talking with the patient, they admit to ingesting the entire bottle of their antidepressant as a suicide attempt. They do not remember the name of the drug they ingested. You obtain an EKG which looks like the one below. Based on this EKG, what type of medication do you think they ingested? (free response)
7. You are evaluating a patient with history of hypertension who is found to be profoundly hypotensive and bradycardic after ingestion of an unknown substance. You perform standard resuscitation with IV fluids and have started vasopressors without improvement. What medication should you consider giving to increase inotropy? (free response)
8. Despite your best efforts, your patient who was presumed to overdose on calcium channel blockers is getting worse, and you are concerned they will imminently go into cardiac arrest. What is a "last ditch" medication you can try to save the patient? (free response)

### Thursday Quiz

1. A patient is dropped off by family in your ambulance bay. They state they found the patient unresponsive in their bathroom. They know the patient has a history of drug use, but they aren’t sure what they use. You note the patient is significantly hypoxic to the 70’s on room air and is only breathing at a rate of 6 bpm. What medicine should you reach for while preparing for a potential intubation? (free response)
2. A patient is brought in for change in mental status. Was found by family members in his room completely disoriented. They did find several foil packets next to him that they say he sometimes gets from the corner store. They are labeled as incense not for human consumption. His urine drug screen is negative. What do you suspect caused this presentation?
  - Marijuana
  - GHB (gamma-Hydroxybutyric acid)
  - Synthetic Cannabinoids
  - Heroin
3. A patient presents to the ER for altered mental status. He was hanging out with his friends when they thought he fell asleep, but they were unable to wake him up. They state they drank a few beers playing video games today but deny any other drug use. They say he is very healthy and is constantly at the gym and don’t think he has any medical problems. On your evaluation, he is unresponsive with severe respiratory depression. Naloxone does not help. He has no gag reflex on exam. You intubate him. The ICU wants to know what you suspect is going on; what drug do you suspect? (free response)
4. You have been monitoring a patient in the ER for cocaine intoxication. The nurse calls you over to re-evaluate the patient. He has become increasingly more agitated, diaphoretic and now with increasing tachycardia. You obtain the following EKG. What is another drug that can cause an EKG like this? (free response)

### Friday Quiz

1. You are seeing a patient who says he was bitten by a spider yesterday. He has a red lesion on his forearm with a central area of necrosis. Based on this finding, you have a pretty good idea what kind of spider bit him. The patient is asking if he can have an antivenom and if one is available:
  - Yes
  - No
2. It looks like your colleague was bitten by a snake with a triangular head. He seems pale, diaphoretic, and you are concerned he could be going into shock. What treatment should you try to obtain for him? (free response)
3. You have a patient presenting to the ER after they ate a mushroom. They state they ingested it about 2 hours ago, and they have had some mild nausea and vomited once but now feel better. What should you do for this patient?
  - Line, Labs, Benzos, Admit for monitoring
  - Observe in ED and provide reassurance
  - Consider giving N-Acetyl Cysteine (NAC) or penicillin
  - Ask them if they have any more mushrooms and make a salad
4. You have a patient you suspect has organophosphate poisoning. Which symptoms correlate with this toxidrome? (Select more than one answer.)
  - Diarrhea
  - Constipation
  - Salivation
  - Emesis

### Monday Quiz Answers

1. You have a patient presenting after overdosing on lithium. She states she took 100 pills 30 minutes ago. Your attending thinks you should give activated charcoal. Why is this a bad idea?
  - She is not in the time frame activated charcoal would be effective.
  - **Activated Charcoal will not be effective against Lithium**.
2. What is the toxic metabolite of acetaminophen?
  - Glutathione
  - **NAPQI**
  - NAC
  - Acetaminophen Reductase
3. You have a patient who presents after a Tylenol ingestion which took place about 4 hours ago. The patient’s Tylenol level is 250micrograms/ml. What tool can you use to determine if they need NAC?
  - Laboratory value cut off
  - **Nomogram**
4. For the same patient: "You have a patient who presents after a Tylenol ingestion which took place about 4 hours ago. The patient’s Tylenol level is 250micrograms/ml." Based on the nomogram should you give NAC?
  - **Yes**
  - No
5. What is the initial dose of NAC?
  - 250 mg/kg
  - **150 mg/kg**
  - 50 mg/kg
  - 25 mg/kg
6. You have a patient presenting with ringing in her ears. She has been doing a lot of strenuous workouts and applying tiger balm liberally to soothe her aching muscles. What salicylate level would you expect her blood work to show?
  - >10 mg/DL
  - >20 mg/DL
  - **>30 mg/DL**
7. What electrolyte do we especially care about replacing in salicylate overdose?
  - Magnesium
  - **Potassium**
  - Calcium
8. You have a patient presenting to your ER after a house fire. On primary survey their ABC’s are intact, but you notice they are responding inappropriately to questions. Glasgow Coma Scale is 14 with a point taken away for that. While your colleague is performing the secondary survey you are placing orders in the computer. What lab tests should you consider ordering given the patient’s altered mental status? (free response)
  **Answer: Blood carboxyhemoglobin level, arterial blood gas (ABG), and lactate are all acceptable responses**.
9. You have a pregnant patient who is under investigation for possible CO exposure. At what level should you consult your local hyperbaric center for possible dive?
  - >5%
  - >8%
  - **>15%**

### Tuesday Quiz Answers

1. You are seeing a patient for anxiety. Patient states he has been feeling very anxious since this morning. He is tremulous on evaluation and appears diaphoretic. His vitals are notable for being tachycardic and hypertensive. You suspect alcohol withdrawal and want to start treatment. How do most treatments for alcohol withdrawal work?
  - **GABA Agonist**
  - GABA Antagonist
2. A patient presents with altered mental status. You obtain a head CT which is normal and basic labs are unremarkable except for an anion gap of 32. What do you suspect could be causing his altered mental status? (select all that apply)
  - **Methanol**
  - Ethanol
  - Cocaine
  - **Ethylene glycol**
3. For the same patient, the wife finally arrives and you obtain history that he was found in the garage next to an empty bottle of antifreeze. What treatment should you start immediately? (free response section)
  **Answer: fomepizole**
4. It turns out the hospital has only enough fomepizole for one dose; what other treatment modalities could you consider? (free response)
  **Answer: Acceptable responses are ethanol, dialysis, folate, thiamine, pyridoxine**.

### Wednesday Quiz Answers

1. What is the main concerning abnormal finding on this EKG?
  - STEMI
  - Hyperkalemia
  - **Long QT**
  - Widened QRS
2. What rhythm can a prolonged QT interval lead to?
  - Atrial fibrillation
  - Bidirectional tachycardia
  - **Torsades de points**
  - PEA
3. You have a patient presenting with fever and tachycardia after overdosing on their citalopram (an SSRI). In addition to supportive care (cooling, benzodiazepines, etc) what medication could you consider administering? (free response)
  **Answer: Cyproheptadine**.
4. You have a patient presenting with hyperthermia and altered mental status. You discuss the case with the attending and express your concern for serotonin syndrome since you are now an expert in this. The attending wants to know why you think it’s not malignant hyperthermia or neuroleptic malignant syndrome. What key physical exam finding will help you differentiate these?
  - Wet Skin/Diaphoresis
  - Findings of empty pill bottles in pockets
  - **Ankle clonus on neuro exam**
  - Pupils appear normal
5. What is the main concerning abnormal finding on this EKG?
  - STEMI
  - Hyperkalemia
  - Prolonged QT
  - **Widened QRS**
6. What’s the toxic dose of lidocaine without epinephrine added?
  - 4 mg/kg
  - **5 mg/kg**
  - 6 mg/kg
  - 7 mg/kg
7. You have a patient who arrives to the ER for suicidal ideation. After talking with the patient, they admit to ingesting the entire bottle of their antidepressant as a suicide attempt. They do not remember the name of the drug they ingested. You obtain an EKG which looks like the one below. Based on this EKG, what type of medication do you think they ingested? (free response)
  **Answer: Tricyclic Antidepressant (TCA)**.
8. You are evaluating a patient with history of hypertension who is found to be profoundly hypotensive and bradycardic after ingestion of an unknown substance. You perform standard resuscitation with IV fluids and have started vasopressors without improvement. What medication should you consider giving to increase inotropy? (free response)
  **Answer: Acceptable responses are high dose insulin, HDI (hexamethylene diisocyanate), insulin**
9. Despite your best efforts, your patient who was presumed to overdose on calcium channel blockers is getting worse, and you are concerned they will imminently go into cardiac arrest. What is a "last ditch" medication you can try to save the patient? (free response)
  **Answer: lipid emulsion therapy, intralipid**.

### Thursday Quiz Answers

1. A patient is dropped off by family in your ambulance bay. They state they found the patient unresponsive in their bathroom. They know the patient has a history of drug use, but they aren’t sure what they use. You note the patient is significantly hypoxic to the 70’s on room air and is only breathing at a rate of 6 bpm. What medicine should you reach for while preparing for a potential intubation? (free response)
  **Answer: Narcan, naloxone**.
2. A patient is brought in for change in mental status. Was found by family members in his room completely disoriented. They did find several foil packets next to him that they say he sometimes gets from the corner store. They are labeled as incense not for human consumption. His urine drug screen is negative. What do you suspect caused this presentation?
  - Marijuana
  - GHB (gamma-Hydroxybutyric acid)
  - **Synthetic Cannabinoids**
  - Heroin
3. A patient presents to the ER for altered mental status. He was hanging out with his friends when they thought he fell asleep, but they were unable to wake him up. They state they drank a few beers playing video games today but deny any other drug use. They say he is very healthy and is constantly at the gym and don’t think he has any medical problems. On your evaluation, he is unresponsive with severe respiratory depression. Naloxone does not help. He has no gag reflex on exam. You intubate him. The ICU wants to know what you suspect is going on; what drug do you suspect? (free response)
  **Answer: GHB (Gamma Hydroxybutyrate)**.
4. You have been monitoring a patient in the ER for cocaine intoxication. The nurse calls you over to reevaluate the patient. He has become increasingly more agitated, diaphoretic and now with increasing tachycardia. You obtain the following EKG. What is another drug that can cause an EKG like this? (free response)
  **Answer: Acceptable responses are lidocaine, TCA, anesthetic, carbamazepine, antiarrhythmics, antimalarials**.

### Friday Quiz Answers

1. You are seeing a patient who says he was bitten by a spider yesterday. He has a red lesion on his forearm with a central area of necrosis. Based on this finding, you have a pretty good idea what kind of spider bit him. The patient is asking if he can have an antivenom and if one is available:
  - Yes
  - **No**
2. It looks like your colleague was bitten by a snake with a triangular head. He seems pale, diaphoretic, and you are concerned he could be going into shock. What treatment should you try to obtain for him? (free response)
  **Answer: Crofab**.
3. You have a patient presenting to the ER after they ate a mushroom. They state they ingested it about 2 hours ago, and they have had some mild nausea and vomited once but now feel better. What should you do for this patient?
  - Line, Labs, Benzos, Admit for monitoring
  - **Observe in ED and provide reassurance**
  - Consider giving N-Acetyl Cysteine (NAC) or penicillin
  - Ask them if they have any more mushrooms and make a salad
4. You have a patient you suspect has organophosphate poisoning. Which symptoms correlate with this toxidrome? (Select more than one answer.)
  - **Diarrhea**
  - Constipation
  - **Salivation**
  - **Emesis**
