# Supplementary material for: Management of Poisoned Patients: Implementing a Blended Toxicology Curriculum for Emergency Medicine Residents
Source: J Educ Teach Emerg Med. 2022 Apr 15;7(2):C1–C32. doi: 10.21980/J8C937 (PMC10332743; doi:10.21980/J8C937)
Supplement: Supplementary file 1 [file JETem-7-2-c1-supp1.pptx]

## Slide 1
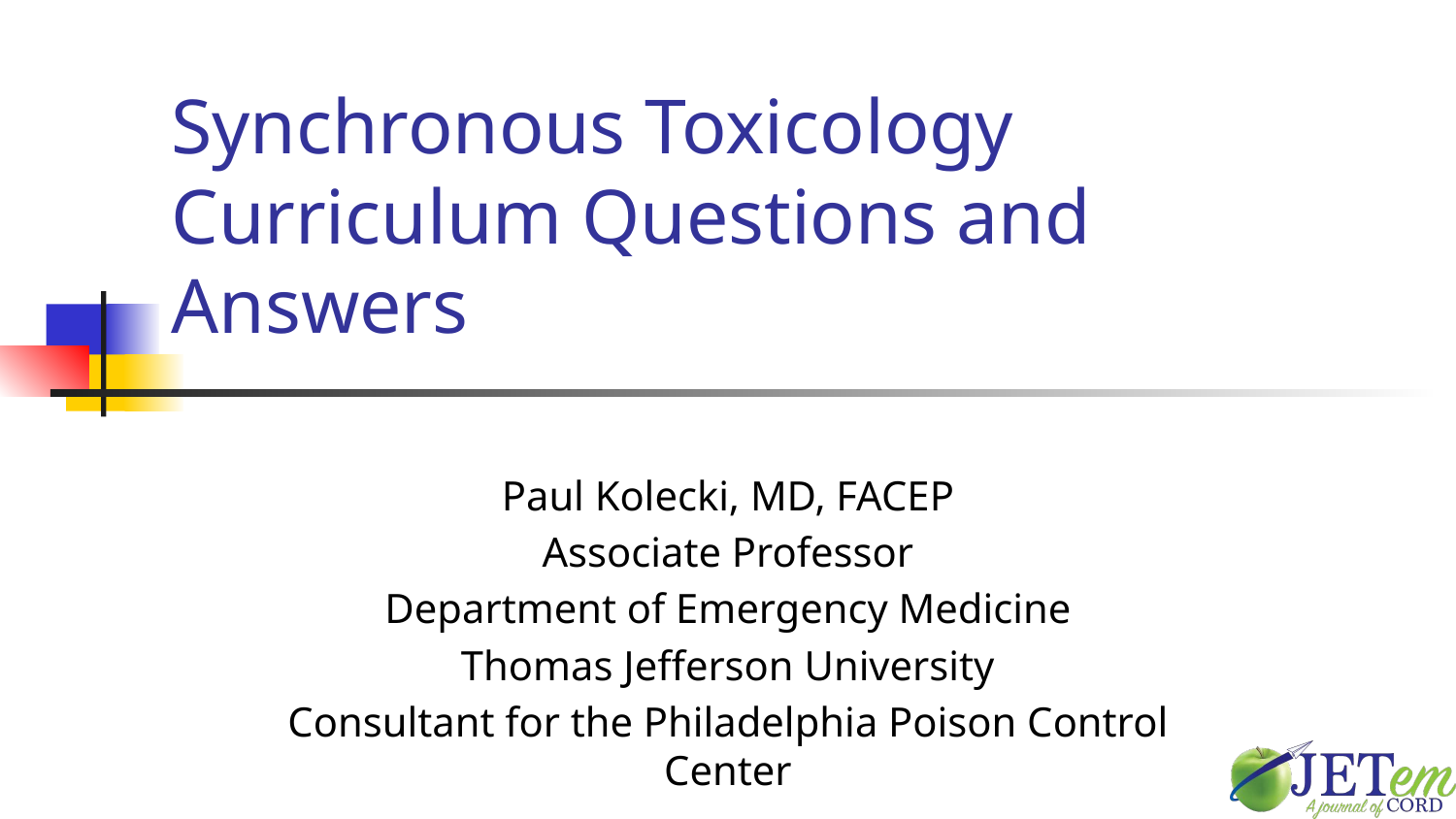

# Synchronous Toxicology Curriculum Questions and Answers
Paul Kolecki, MD, FACEP
Associate Professor
Department of Emergency Medicine
Thomas Jefferson University
Consultant for the Philadelphia Poison Control Center

## Slide 2
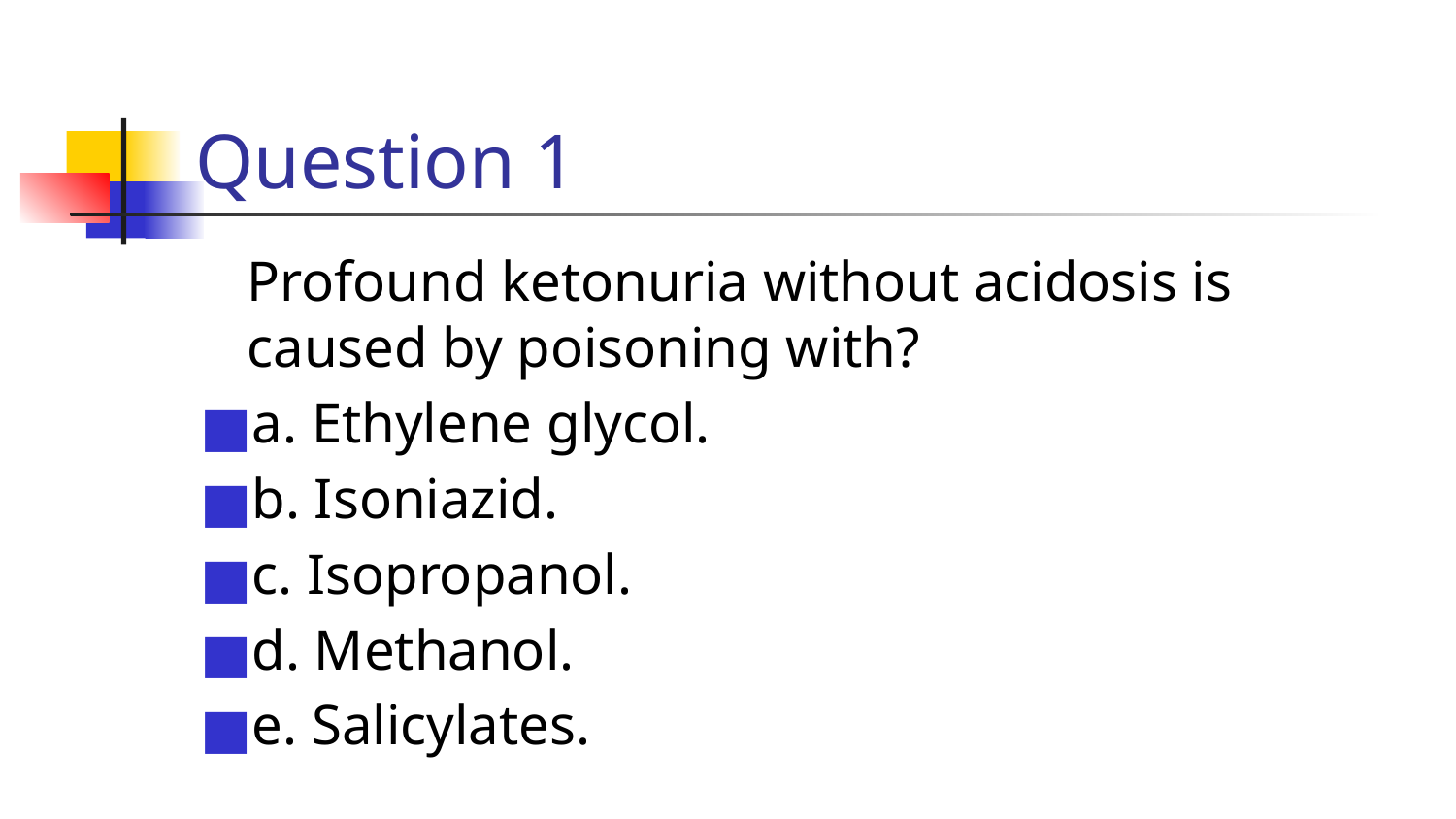

# Question 1
	Profound ketonuria without acidosis is caused by poisoning with?
a. Ethylene glycol.
b. Isoniazid.
c. Isopropanol.
d. Methanol.
e. Salicylates.

## Slide 3
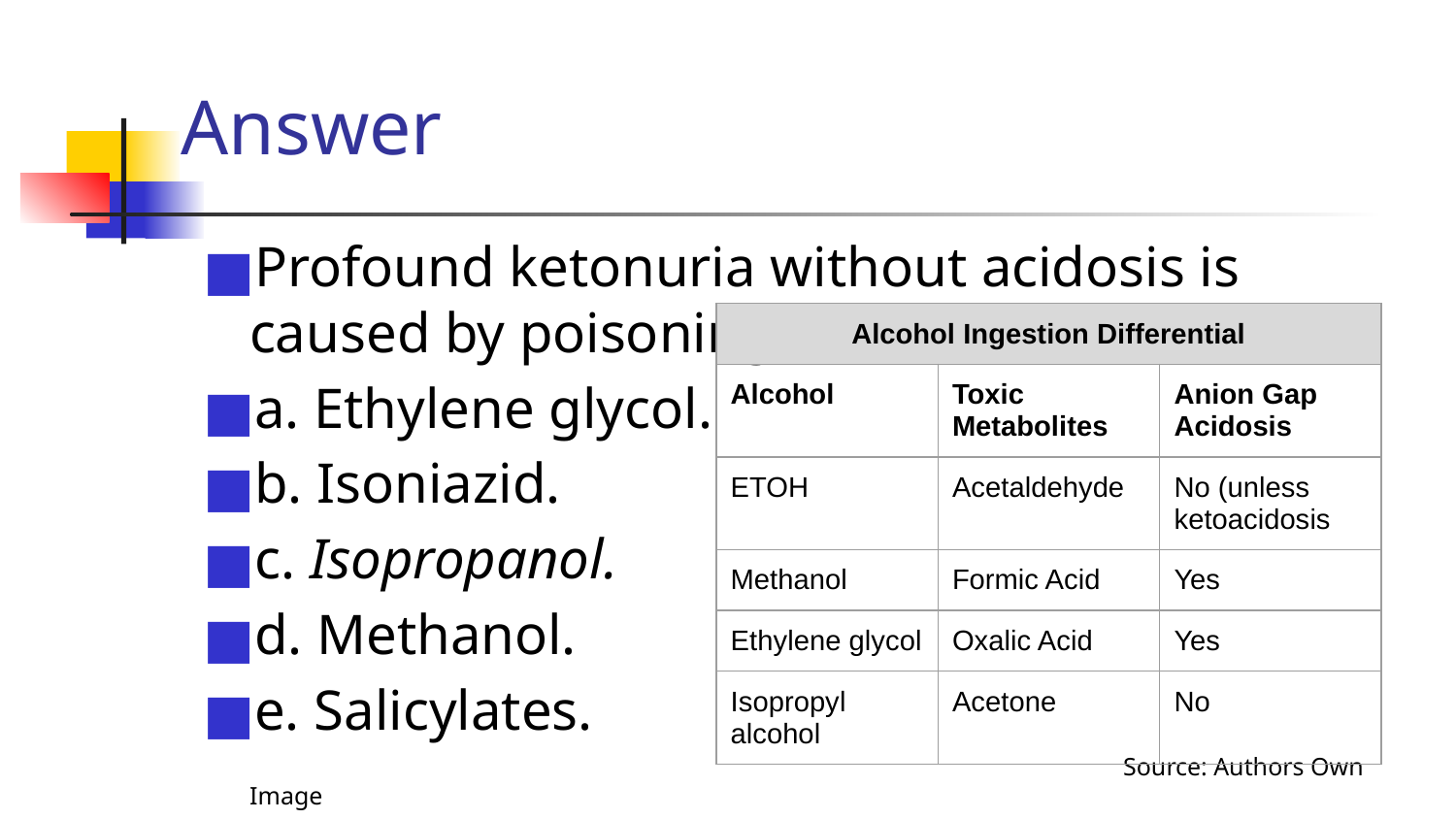

# Answer
Profound ketonuria without acidosis is caused by poisoning with?
a. Ethylene glycol.
b. Isoniazid.
c. Isopropanol.
d. Methanol.
e. Salicylates.
							Source: Authors Own Image
| Alcohol Ingestion Differential | | |
| --- | --- | --- |
| Alcohol | Toxic Metabolites | Anion Gap Acidosis |
| ETOH | Acetaldehyde | No (unless ketoacidosis |
| Methanol | Formic Acid | Yes |
| Ethylene glycol | Oxalic Acid | Yes |
| Isopropyl alcohol | Acetone | No |

## Slide 4
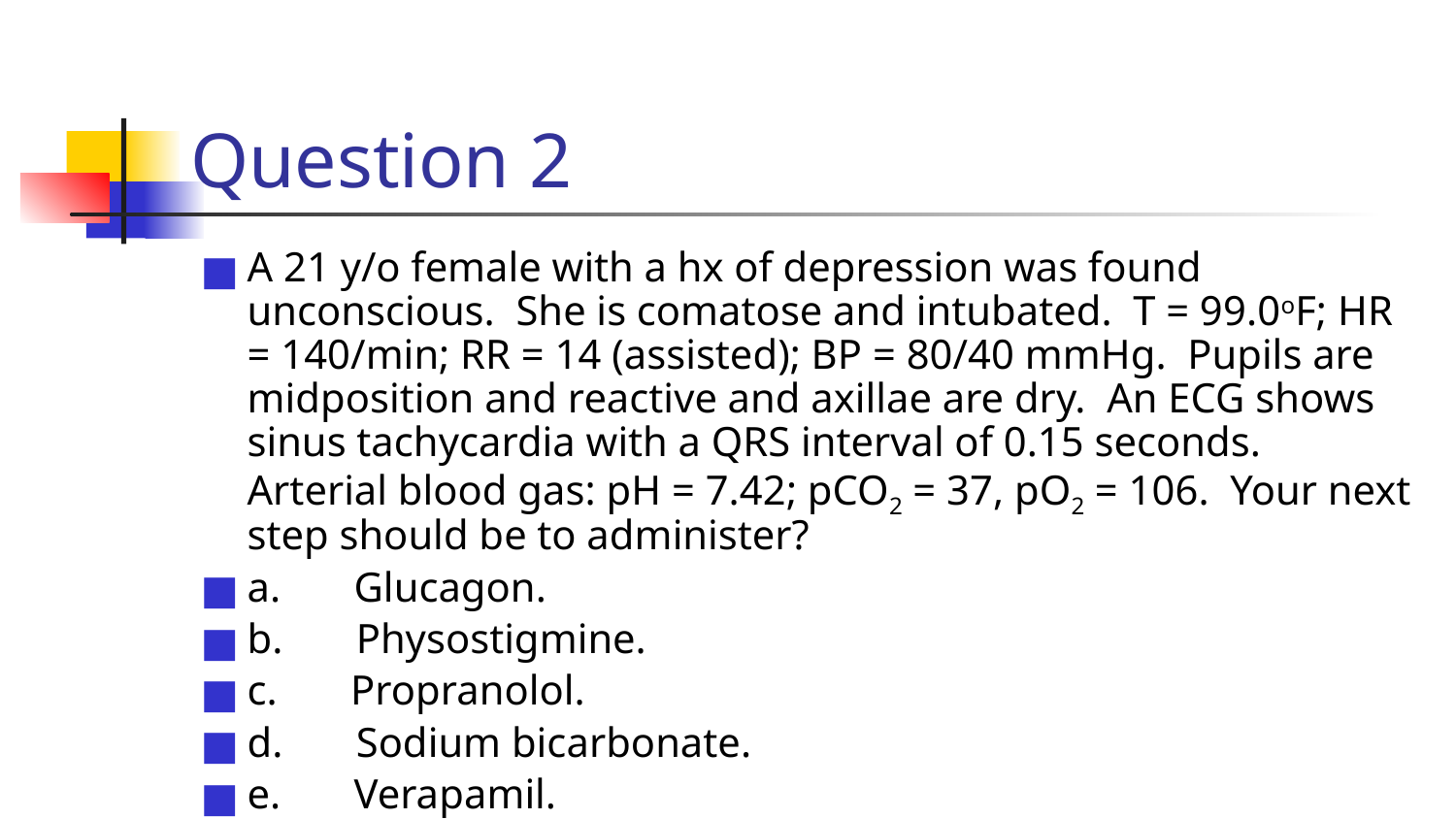

# Question 2
A 21 y/o female with a hx of depression was found unconscious. She is comatose and intubated. T = 99.0oF; HR = 140/min; RR = 14 (assisted); BP = 80/40 mmHg. Pupils are midposition and reactive and axillae are dry. An ECG shows sinus tachycardia with a QRS interval of 0.15 seconds. Arterial blood gas: pH = 7.42; pCO2 = 37, pO2 = 106. Your next step should be to administer?
a.       Glucagon.
b.       Physostigmine.
c.       Propranolol.
d.       Sodium bicarbonate.
e.       Verapamil.

## Slide 5
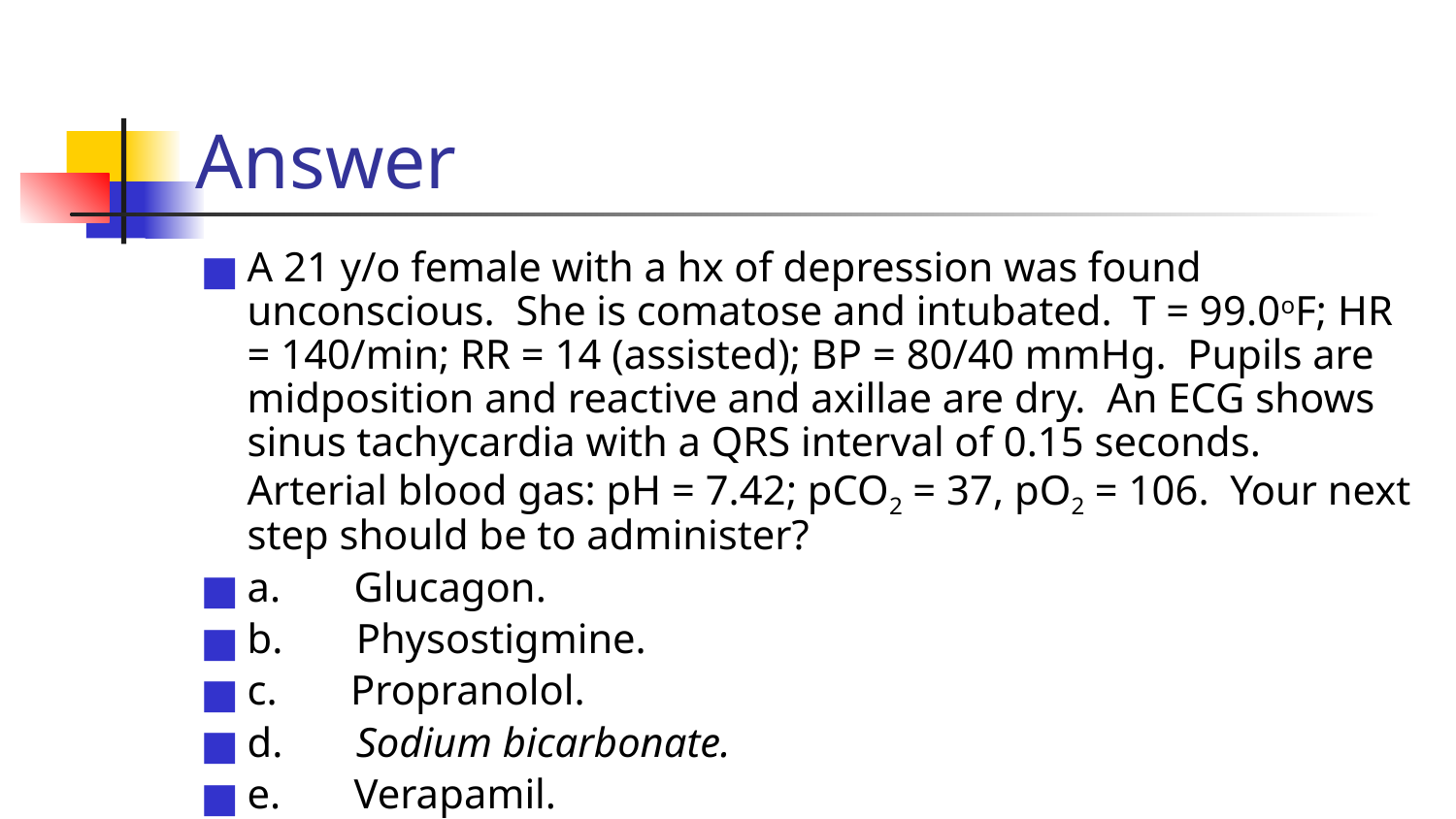

# Answer
A 21 y/o female with a hx of depression was found unconscious. She is comatose and intubated. T = 99.0oF; HR = 140/min; RR = 14 (assisted); BP = 80/40 mmHg. Pupils are midposition and reactive and axillae are dry. An ECG shows sinus tachycardia with a QRS interval of 0.15 seconds. Arterial blood gas: pH = 7.42; pCO2 = 37, pO2 = 106. Your next step should be to administer?
a.       Glucagon.
b.       Physostigmine.
c.       Propranolol.
d.       Sodium bicarbonate.
e.       Verapamil.

## Slide 6
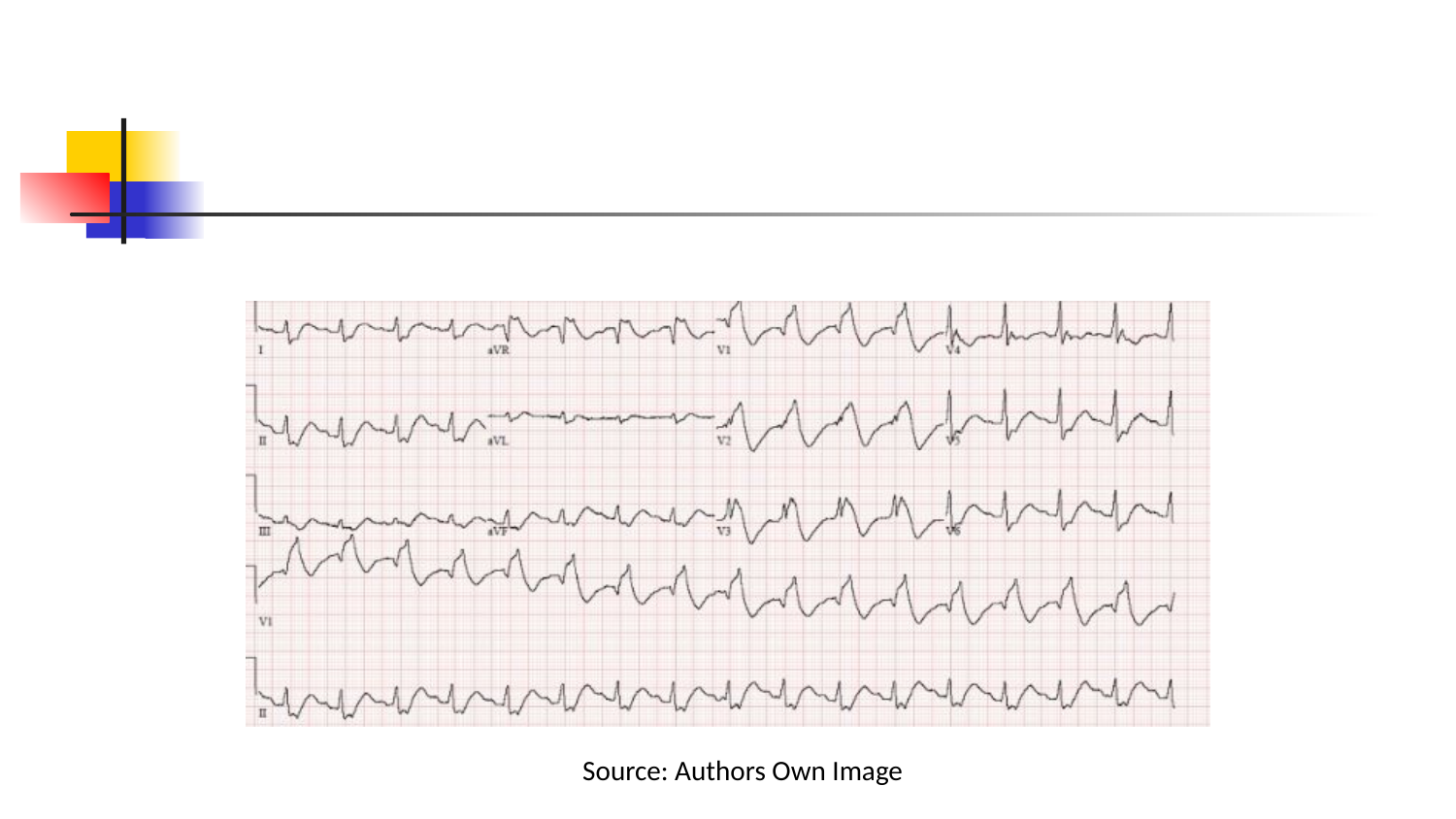

#
Source: Authors Own Image

## Slide 7
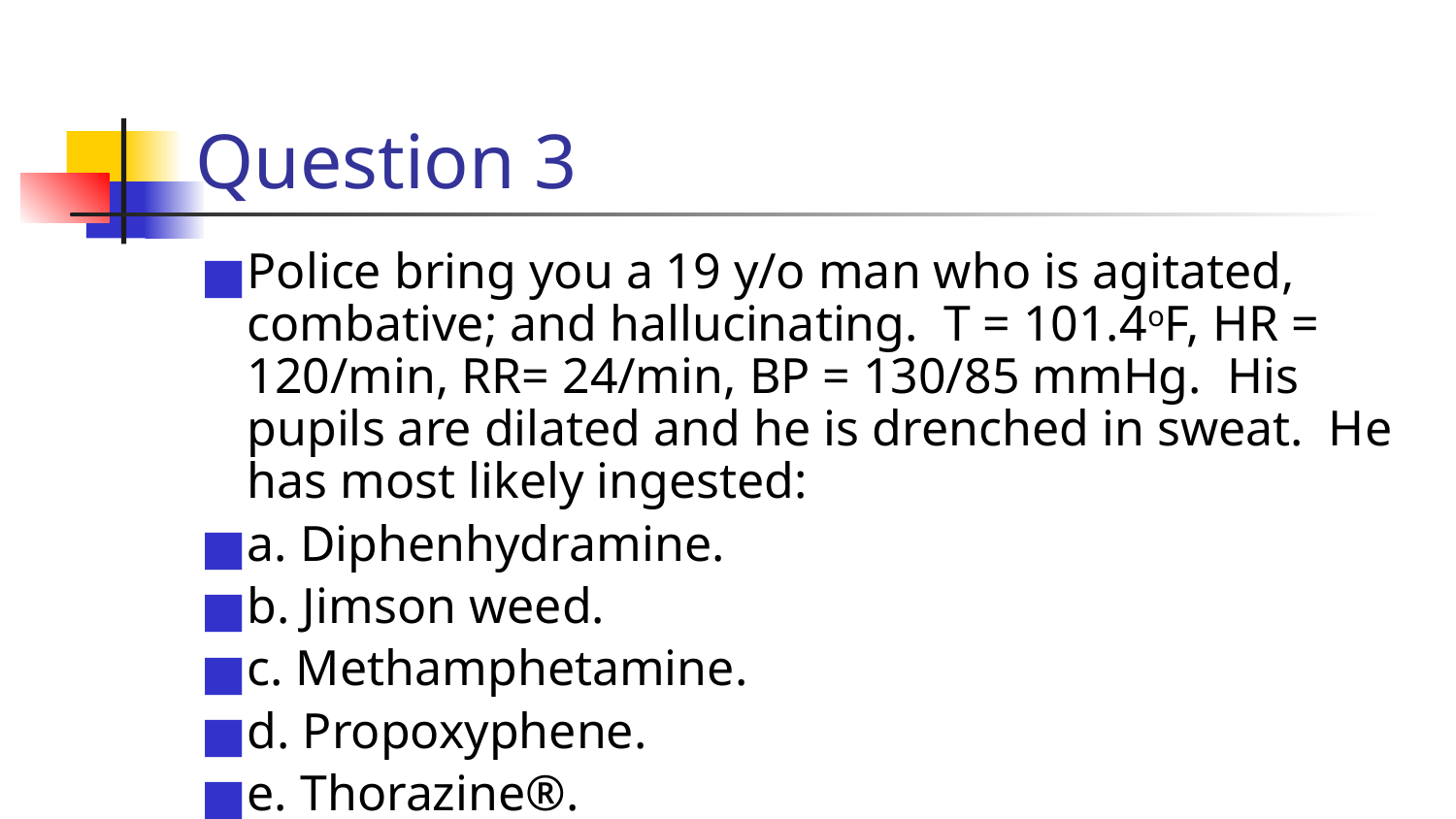

# Question 3
Police bring you a 19 y/o man who is agitated, combative; and hallucinating. T = 101.4oF, HR = 120/min, RR= 24/min, BP = 130/85 mmHg. His pupils are dilated and he is drenched in sweat. He has most likely ingested:
a. Diphenhydramine.
b. Jimson weed.
c. Methamphetamine.
d. Propoxyphene.
e. Thorazine®.

## Slide 8
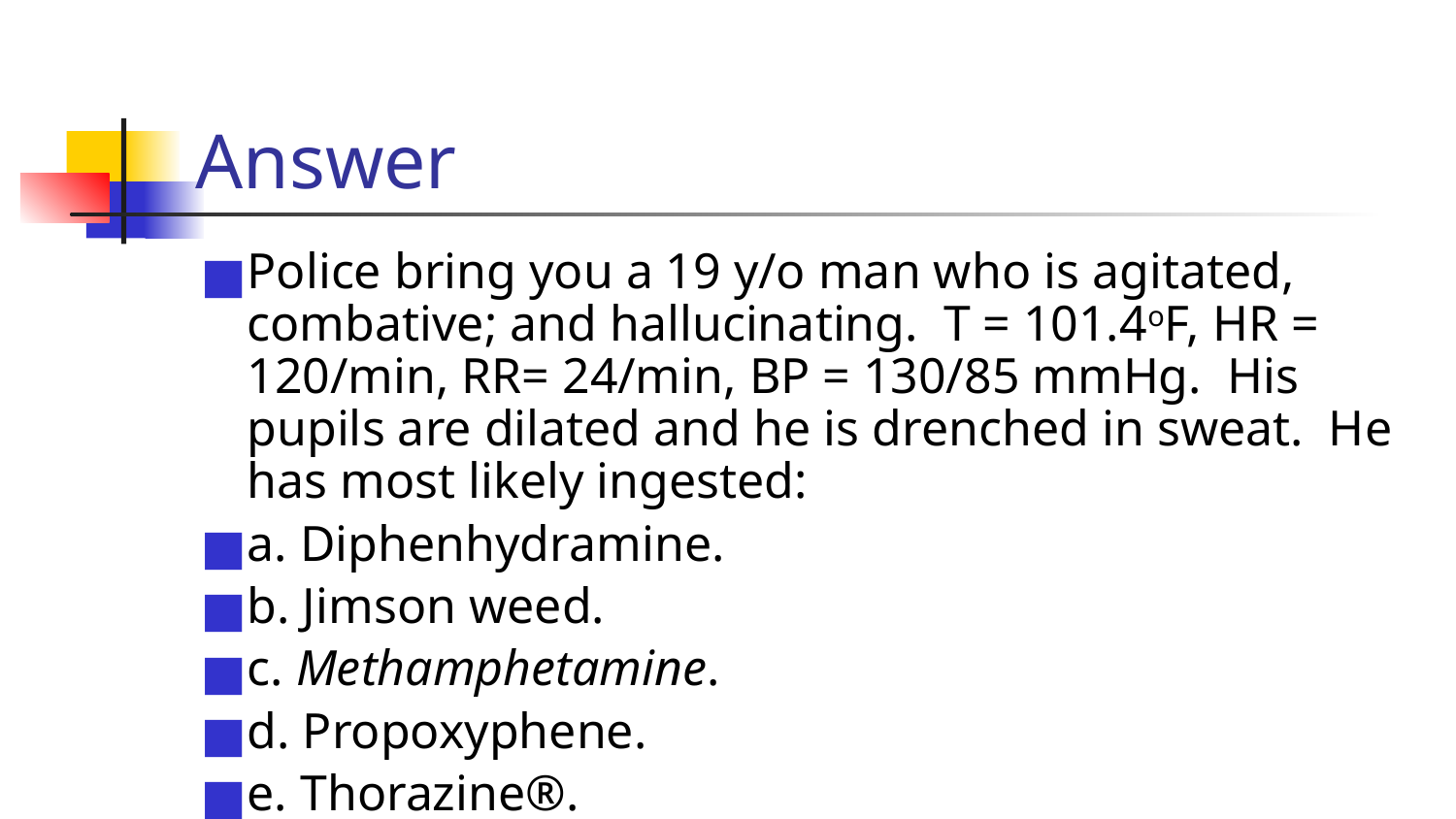

# Answer
Police bring you a 19 y/o man who is agitated, combative; and hallucinating. T = 101.4oF, HR = 120/min, RR= 24/min, BP = 130/85 mmHg. His pupils are dilated and he is drenched in sweat. He has most likely ingested:
a. Diphenhydramine.
b. Jimson weed.
c. Methamphetamine.
d. Propoxyphene.
e. Thorazine®.

## Slide 9
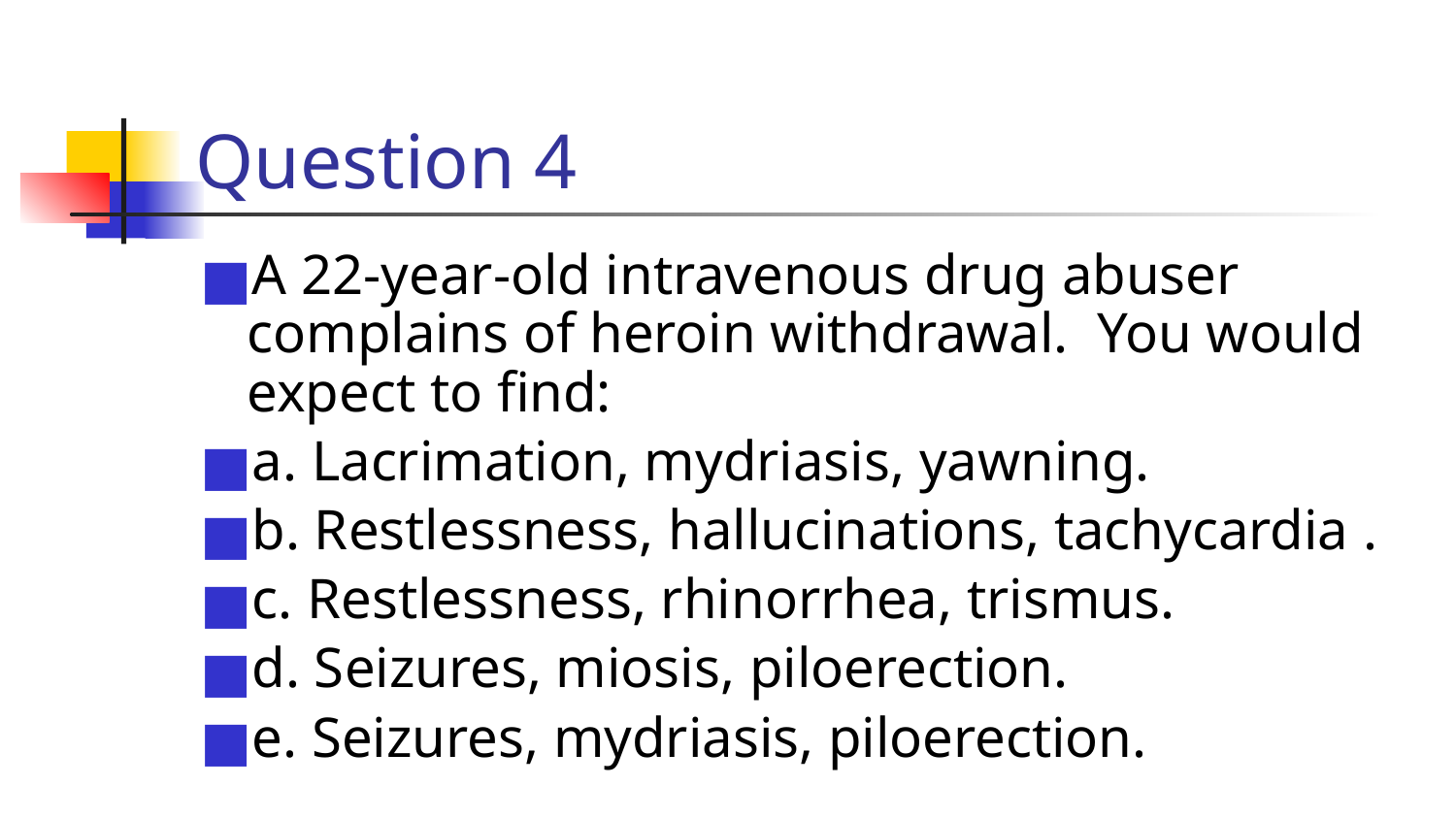

# Question 4
A 22-year-old intravenous drug abuser complains of heroin withdrawal. You would expect to find:
a. Lacrimation, mydriasis, yawning.
b. Restlessness, hallucinations, tachycardia .
c. Restlessness, rhinorrhea, trismus.
d. Seizures, miosis, piloerection.
e. Seizures, mydriasis, piloerection.

## Slide 10
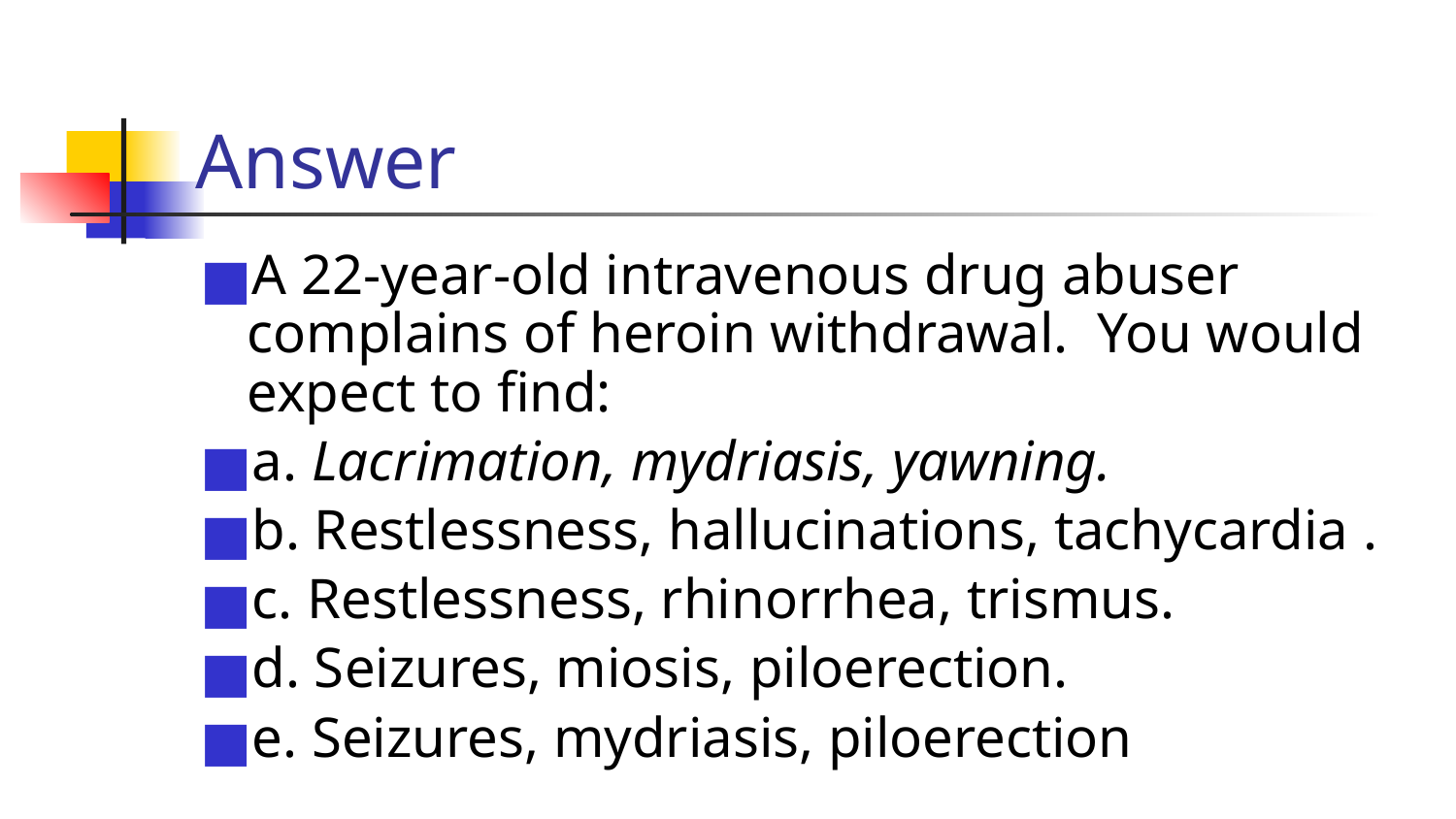

# Answer
A 22-year-old intravenous drug abuser complains of heroin withdrawal. You would expect to find:
a. Lacrimation, mydriasis, yawning.
b. Restlessness, hallucinations, tachycardia .
c. Restlessness, rhinorrhea, trismus.
d. Seizures, miosis, piloerection.
e. Seizures, mydriasis, piloerection

## Slide 11
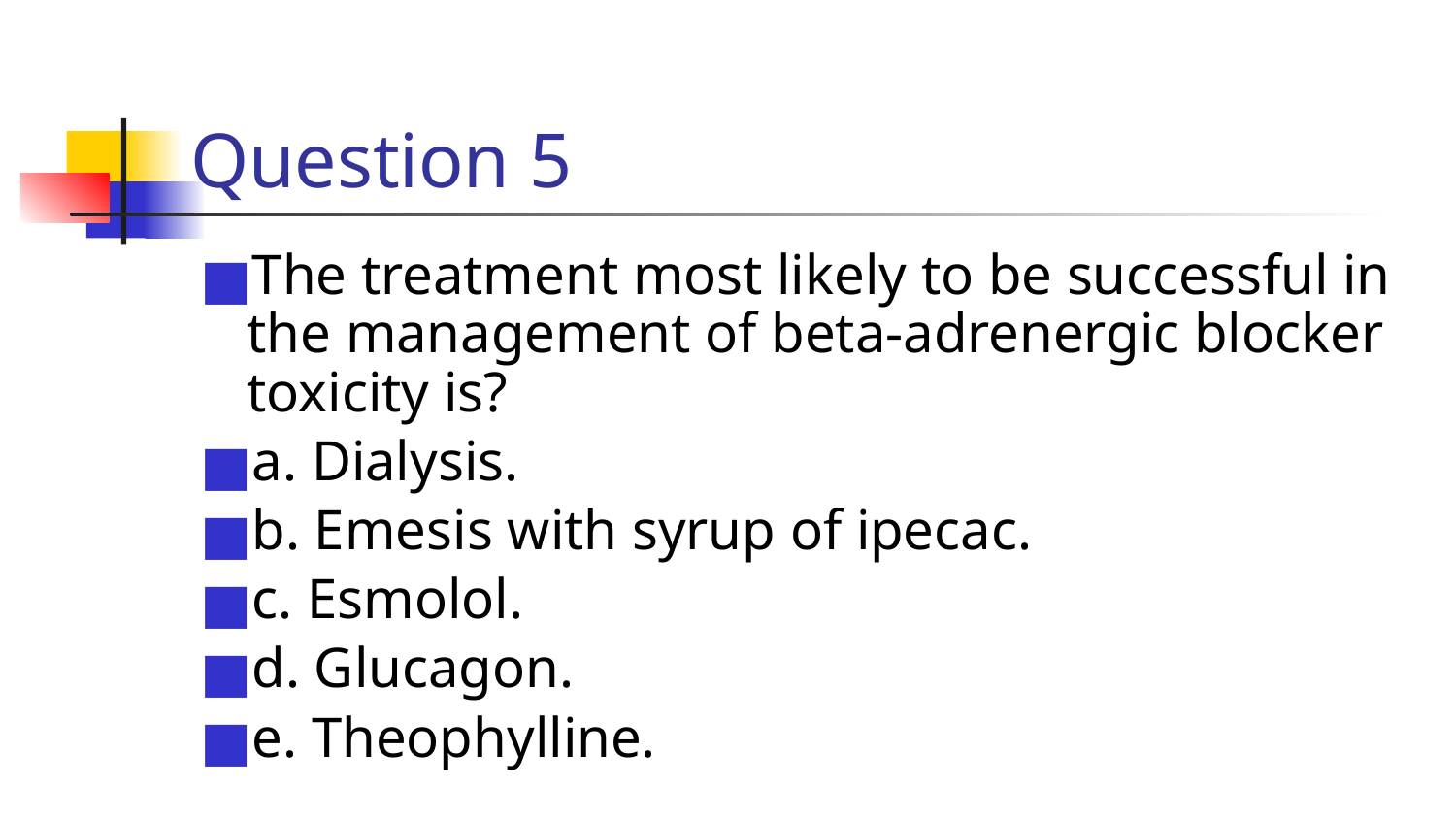

# Question 5
The treatment most likely to be successful in the management of beta-adrenergic blocker toxicity is?
a. Dialysis.
b. Emesis with syrup of ipecac.
c. Esmolol.
d. Glucagon.
e. Theophylline.

## Slide 12
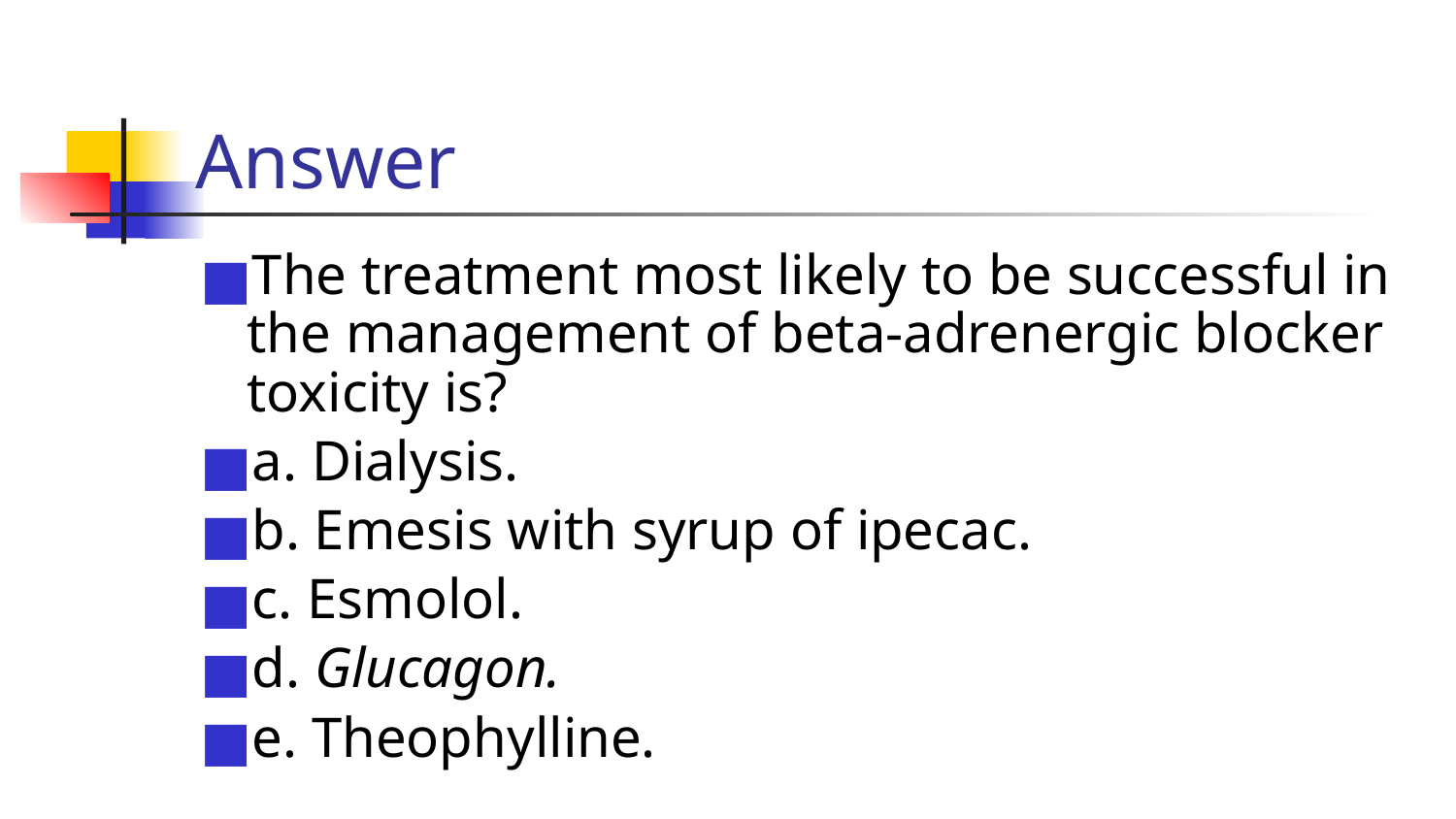

# Answer
The treatment most likely to be successful in the management of beta-adrenergic blocker toxicity is?
a. Dialysis.
b. Emesis with syrup of ipecac.
c. Esmolol.
d. Glucagon.
e. Theophylline.

## Slide 13
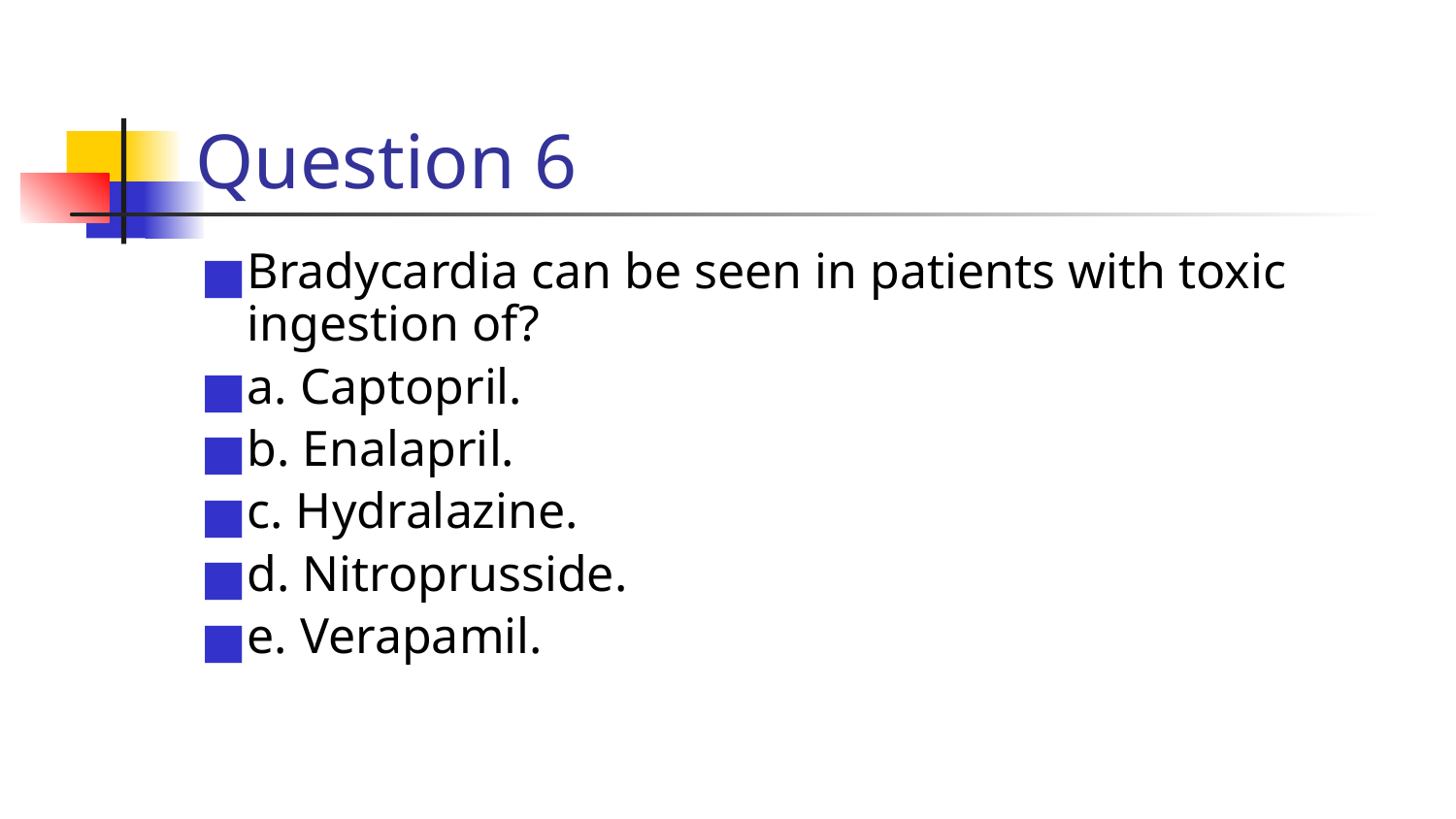

# Question 6
Bradycardia can be seen in patients with toxic ingestion of?
a. Captopril.
b. Enalapril.
c. Hydralazine.
d. Nitroprusside.
e. Verapamil.

## Slide 14
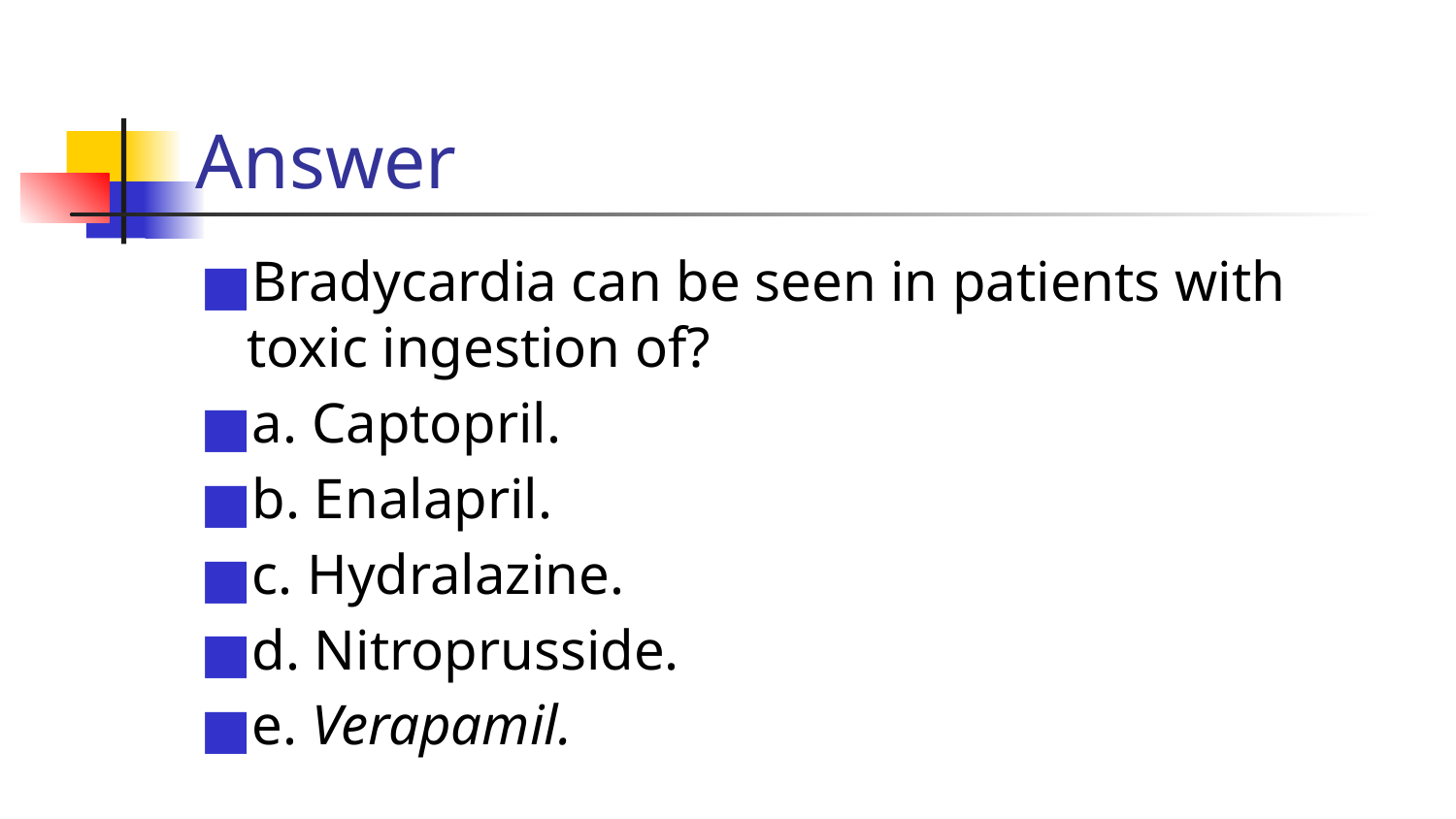

# Answer
Bradycardia can be seen in patients with toxic ingestion of?
a. Captopril.
b. Enalapril.
c. Hydralazine.
d. Nitroprusside.
e. Verapamil.

## Slide 15
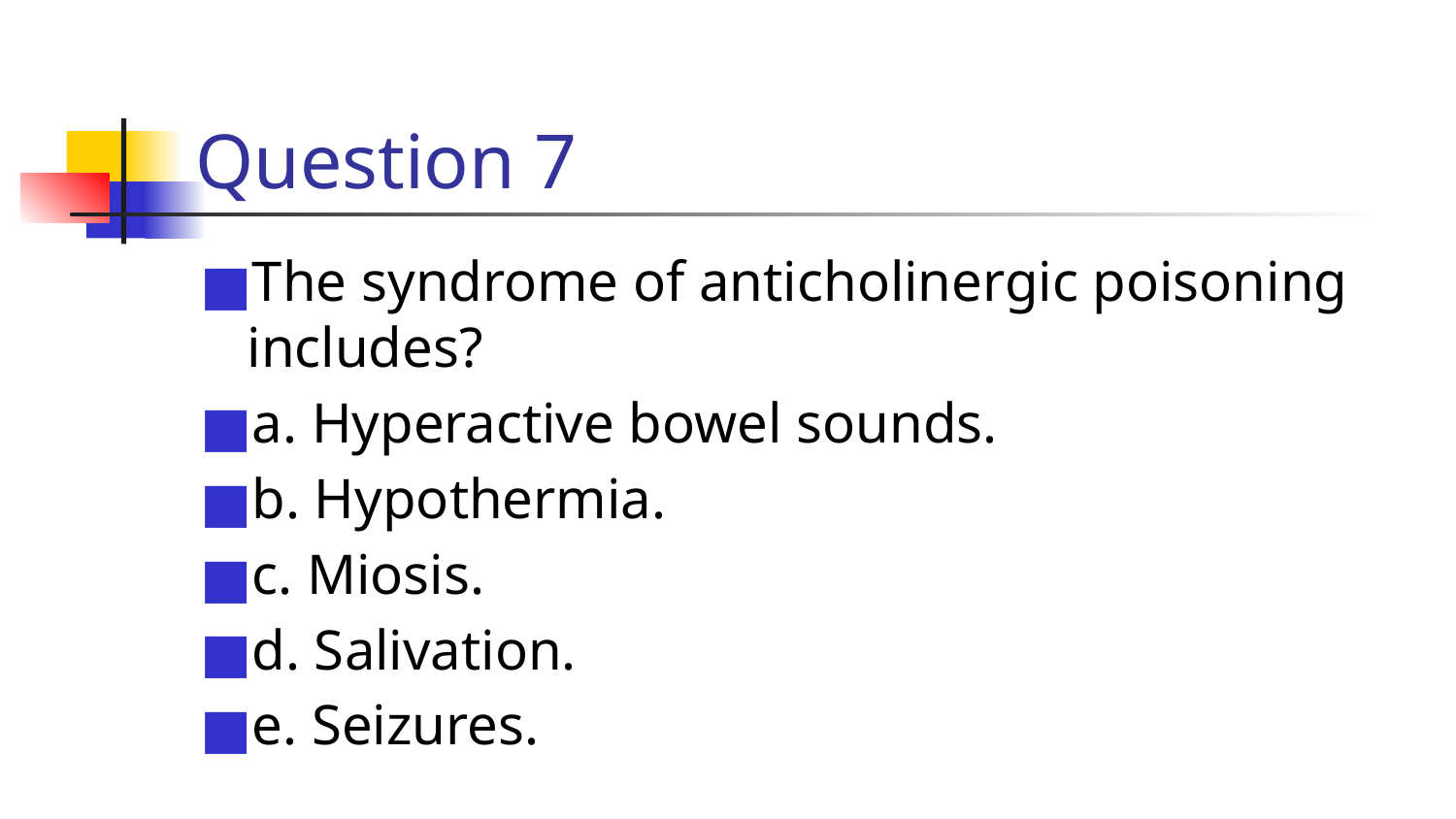

# Question 7
The syndrome of anticholinergic poisoning includes?
a. Hyperactive bowel sounds.
b. Hypothermia.
c. Miosis.
d. Salivation.
e. Seizures.

## Slide 16
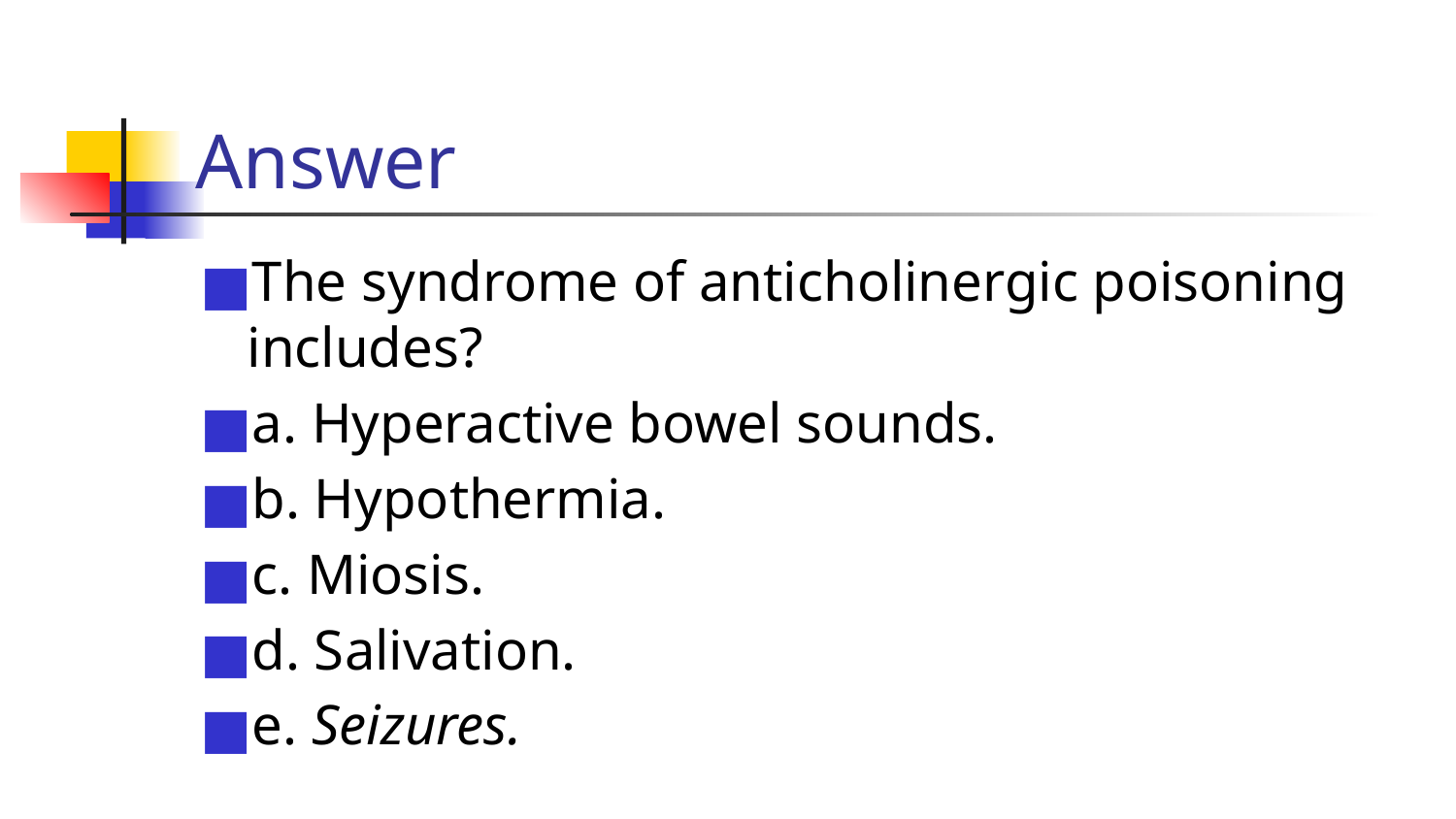

# Answer
The syndrome of anticholinergic poisoning includes?
a. Hyperactive bowel sounds.
b. Hypothermia.
c. Miosis.
d. Salivation.
e. Seizures.

## Slide 17
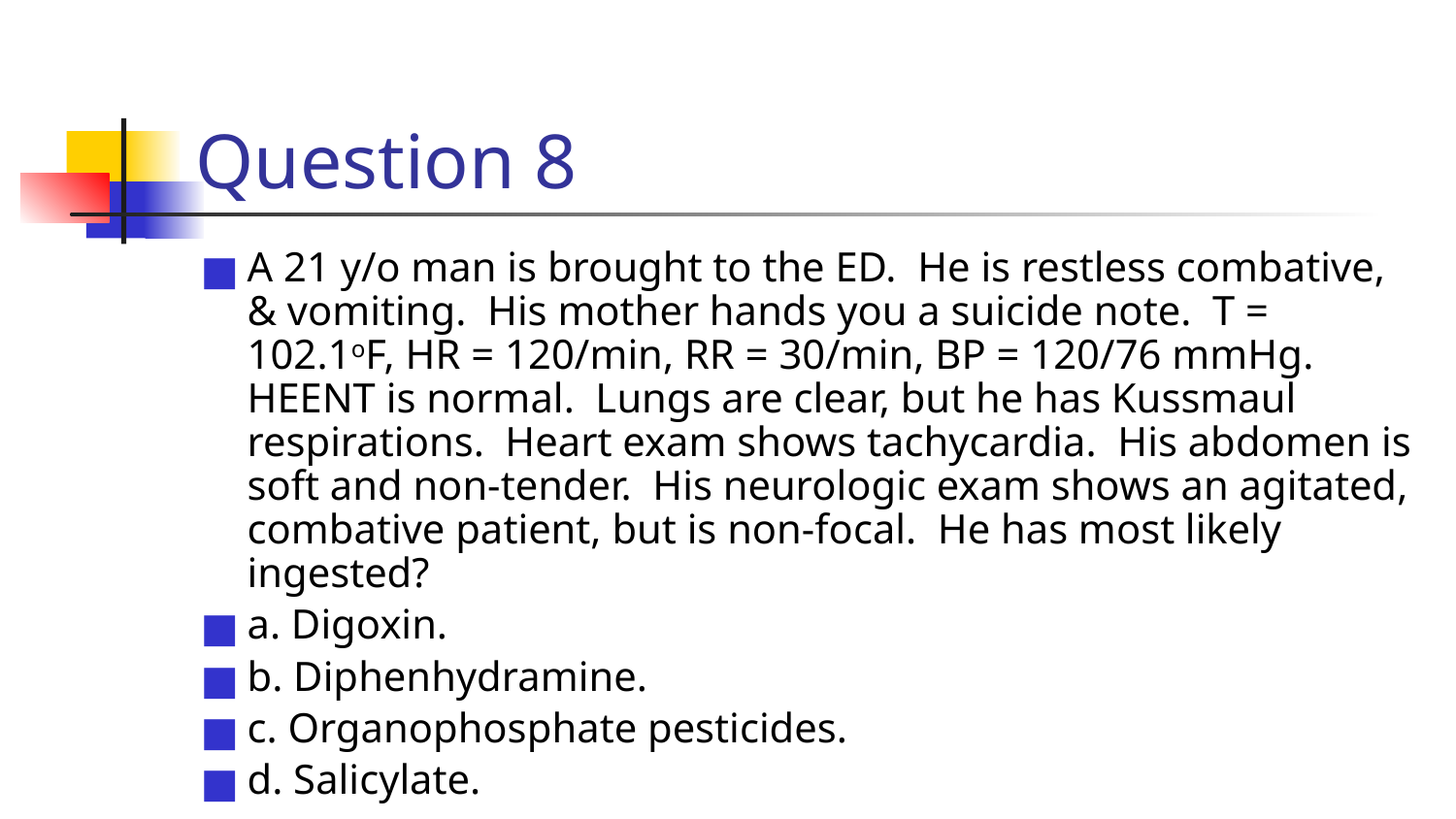

# Question 8
A 21 y/o man is brought to the ED. He is restless combative, & vomiting. His mother hands you a suicide note. T = 102.1oF, HR = 120/min, RR = 30/min, BP = 120/76 mmHg. HEENT is normal. Lungs are clear, but he has Kussmaul respirations. Heart exam shows tachycardia. His abdomen is soft and non-tender. His neurologic exam shows an agitated, combative patient, but is non-focal. He has most likely ingested?
a. Digoxin.
b. Diphenhydramine.
c. Organophosphate pesticides.
d. Salicylate.
e. Tricyclic antidepressants.

## Slide 18
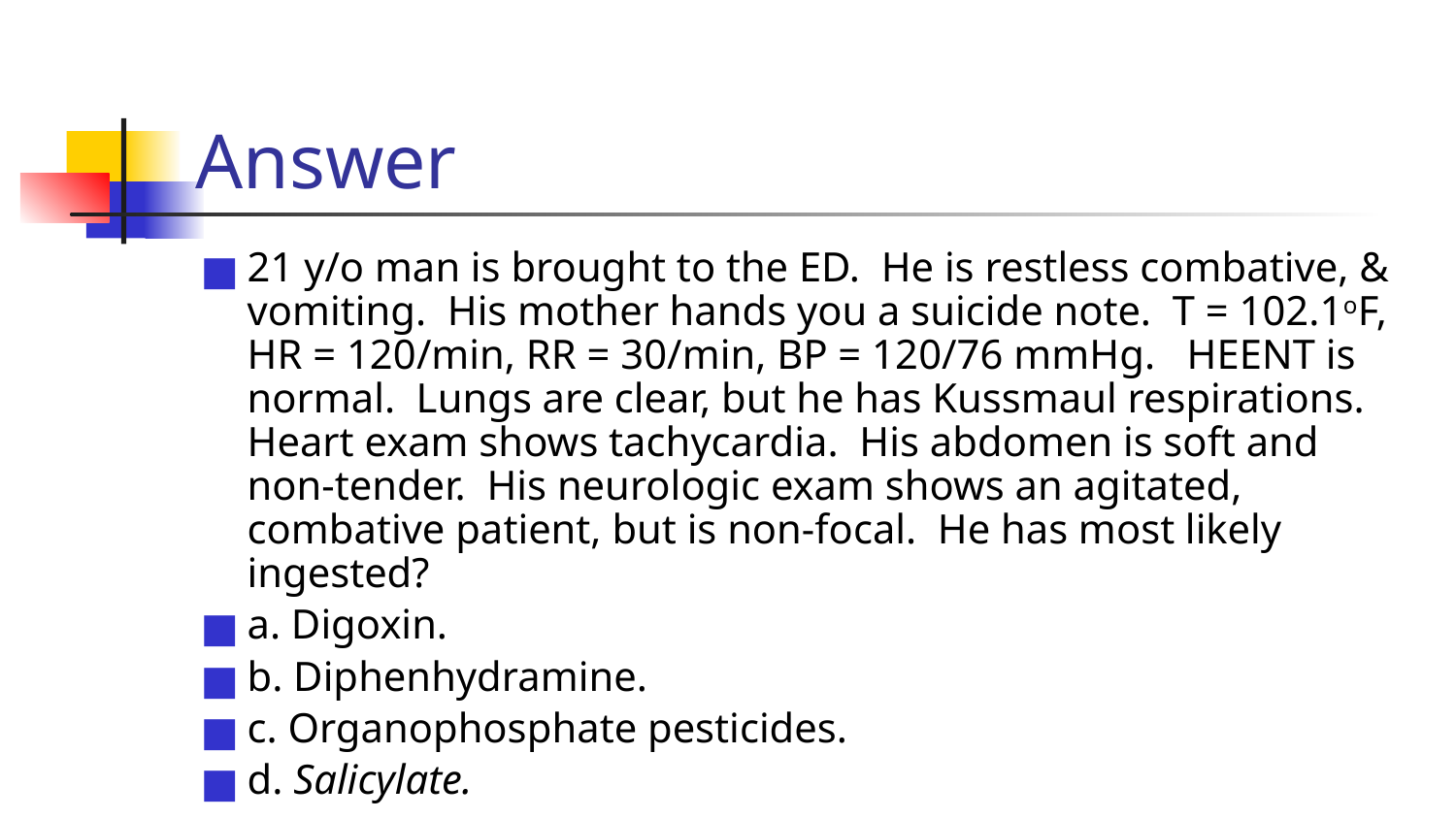

# Answer
21 y/o man is brought to the ED. He is restless combative, & vomiting. His mother hands you a suicide note. T = 102.1oF, HR = 120/min, RR = 30/min, BP = 120/76 mmHg. HEENT is normal. Lungs are clear, but he has Kussmaul respirations. Heart exam shows tachycardia. His abdomen is soft and non-tender. His neurologic exam shows an agitated, combative patient, but is non-focal. He has most likely ingested?
a. Digoxin.
b. Diphenhydramine.
c. Organophosphate pesticides.
d. Salicylate.
e. Tricyclic antidepressants.

## Slide 19
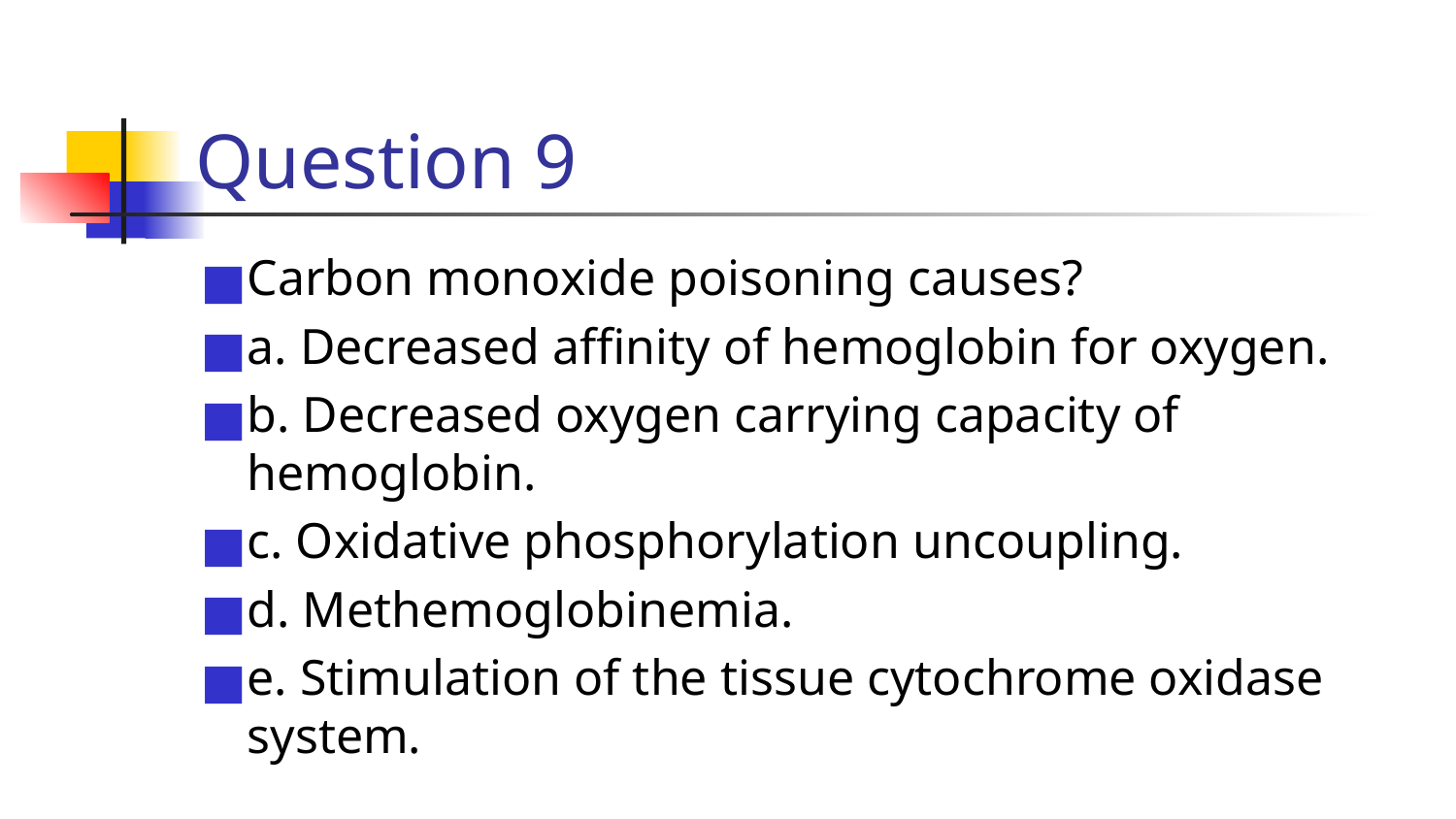

# Question 9
Carbon monoxide poisoning causes?
a. Decreased affinity of hemoglobin for oxygen.
b. Decreased oxygen carrying capacity of hemoglobin.
c. Oxidative phosphorylation uncoupling.
d. Methemoglobinemia.
e. Stimulation of the tissue cytochrome oxidase system.

## Slide 20
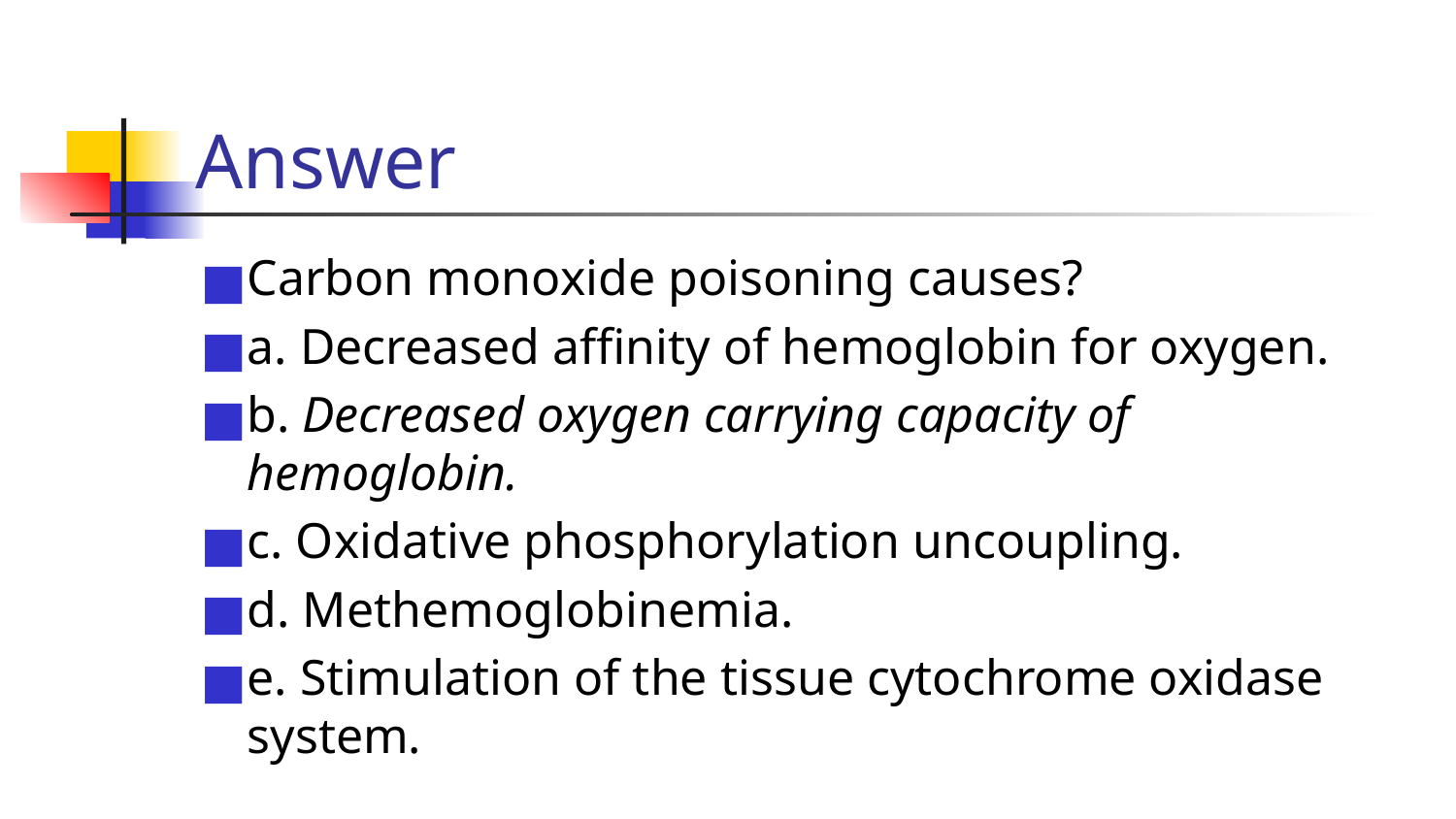

# Answer
Carbon monoxide poisoning causes?
a. Decreased affinity of hemoglobin for oxygen.
b. Decreased oxygen carrying capacity of hemoglobin.
c. Oxidative phosphorylation uncoupling.
d. Methemoglobinemia.
e. Stimulation of the tissue cytochrome oxidase system.

## Slide 21
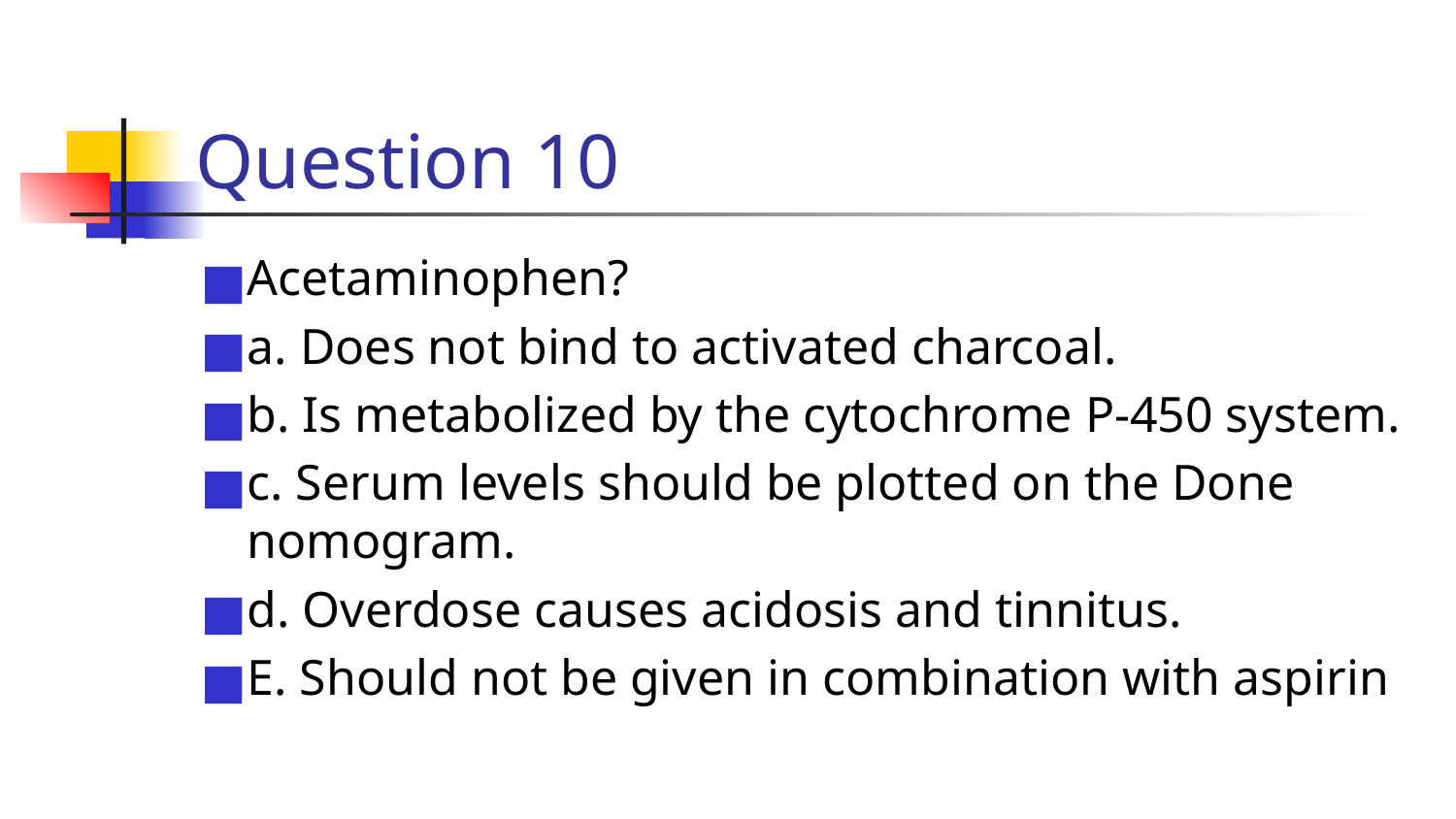

# Question 10
Acetaminophen?
a. Does not bind to activated charcoal.
b. Is metabolized by the cytochrome P-450 system.
c. Serum levels should be plotted on the Done nomogram.
d. Overdose causes acidosis and tinnitus.
E. Should not be given in combination with aspirin

## Slide 22
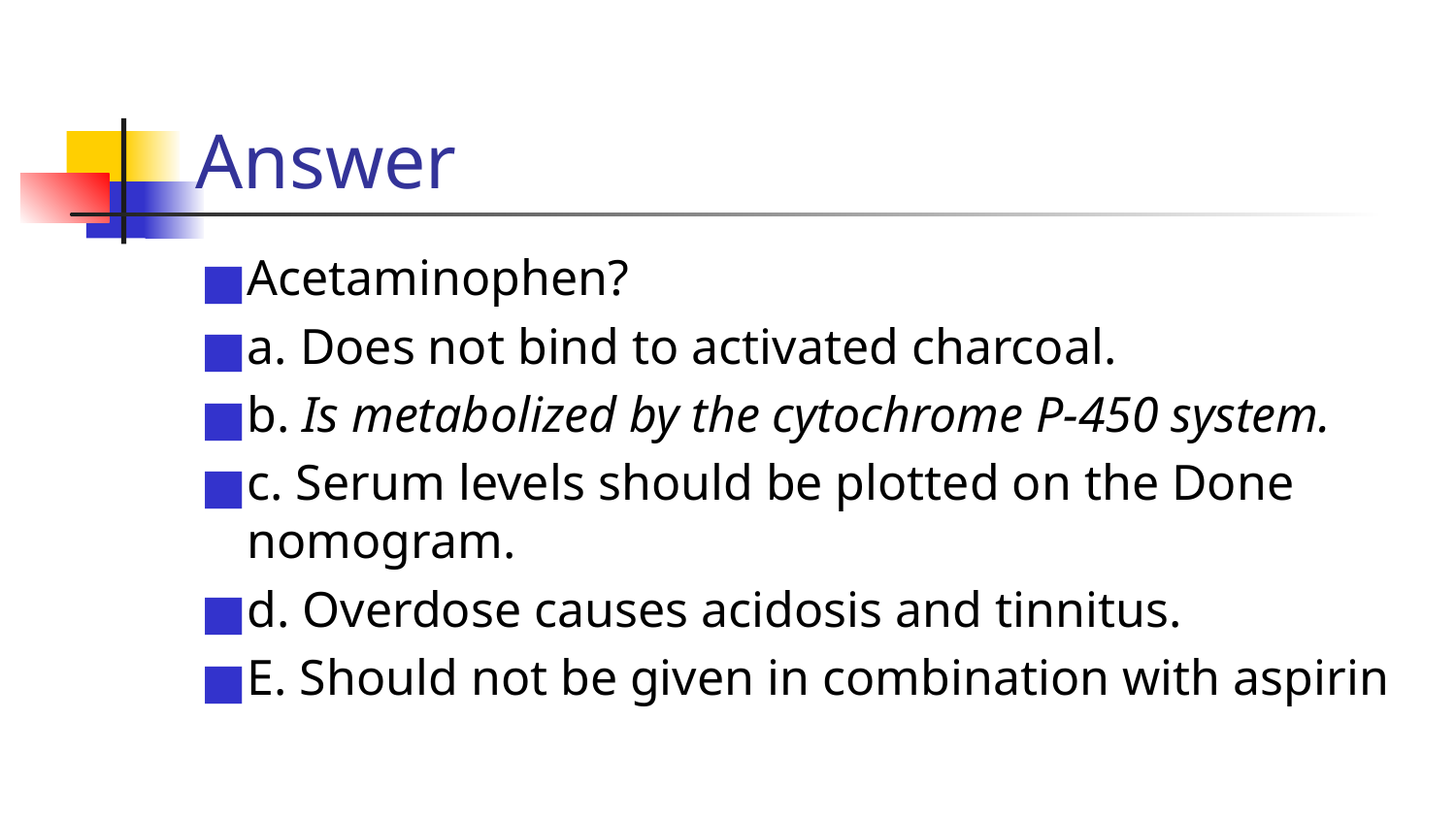

# Answer
Acetaminophen?
a. Does not bind to activated charcoal.
b. Is metabolized by the cytochrome P-450 system.
c. Serum levels should be plotted on the Done nomogram.
d. Overdose causes acidosis and tinnitus.
E. Should not be given in combination with aspirin

## Slide 23
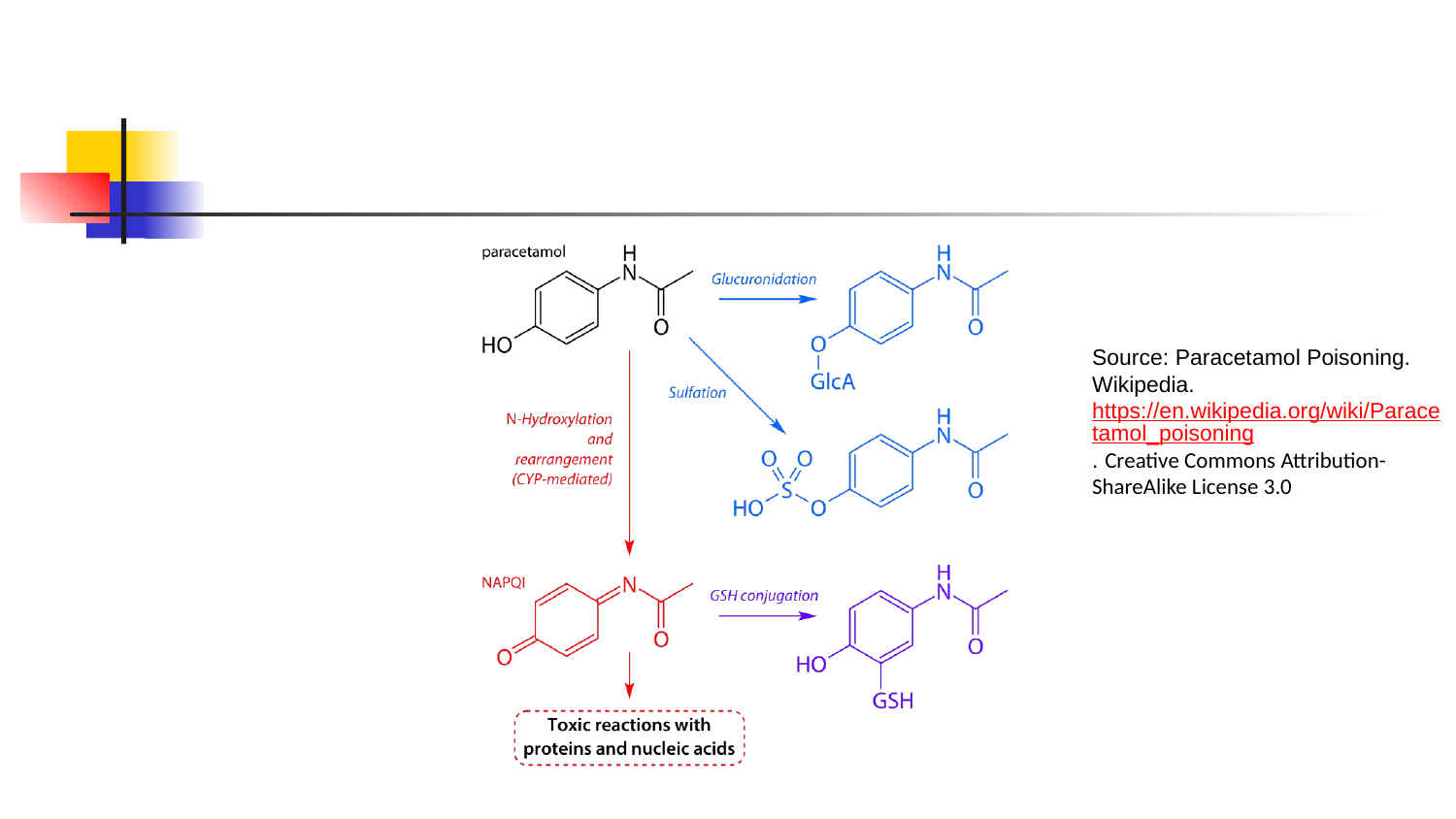

#
Source: Paracetamol Poisoning.
Wikipedia. https://en.wikipedia.org/wiki/Paracetamol_poisoning. Creative Commons Attribution-ShareAlike License 3.0

## Slide 24
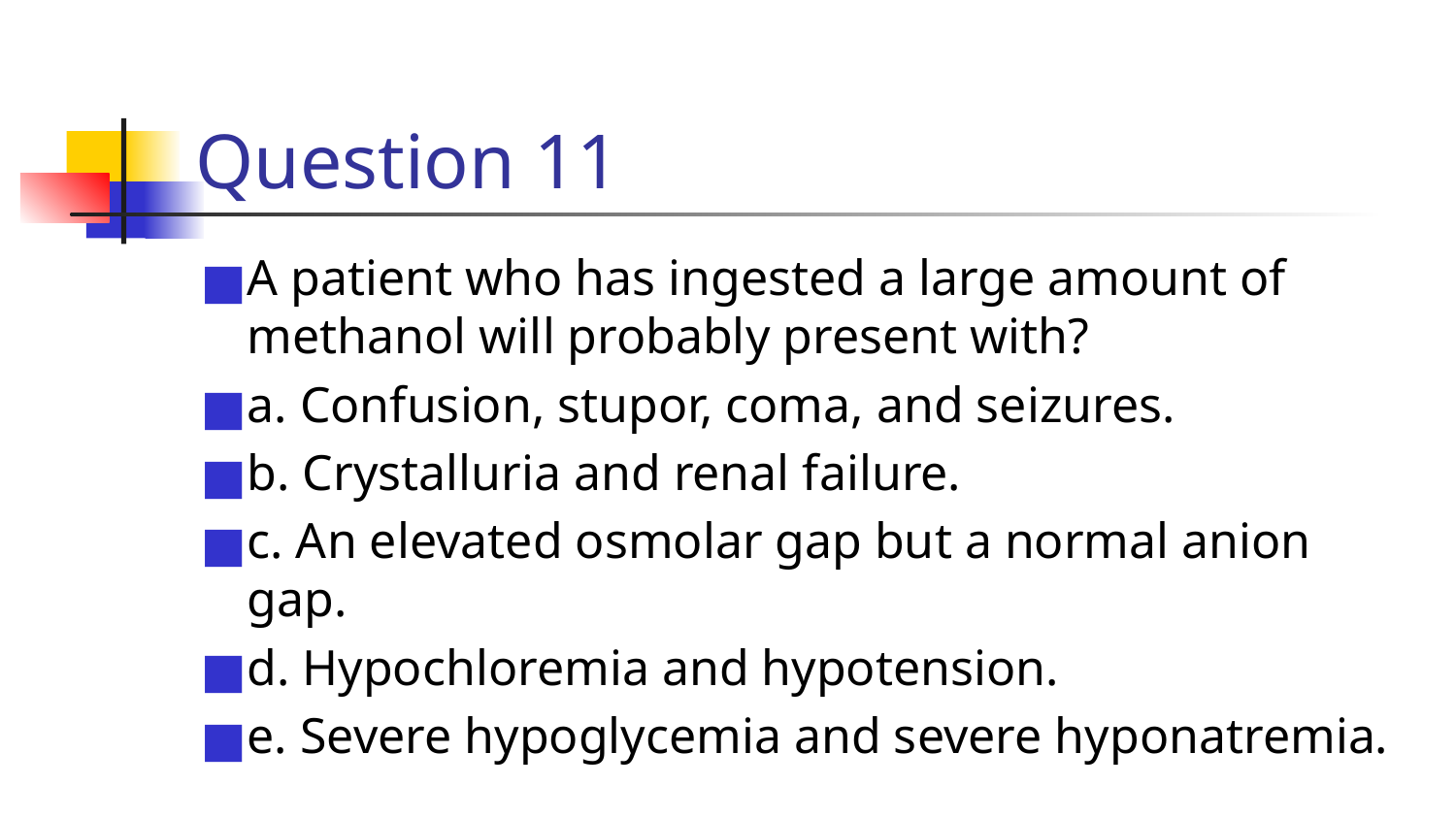

# Question 11
A patient who has ingested a large amount of methanol will probably present with?
a. Confusion, stupor, coma, and seizures.
b. Crystalluria and renal failure.
c. An elevated osmolar gap but a normal anion gap.
d. Hypochloremia and hypotension.
e. Severe hypoglycemia and severe hyponatremia.

## Slide 25
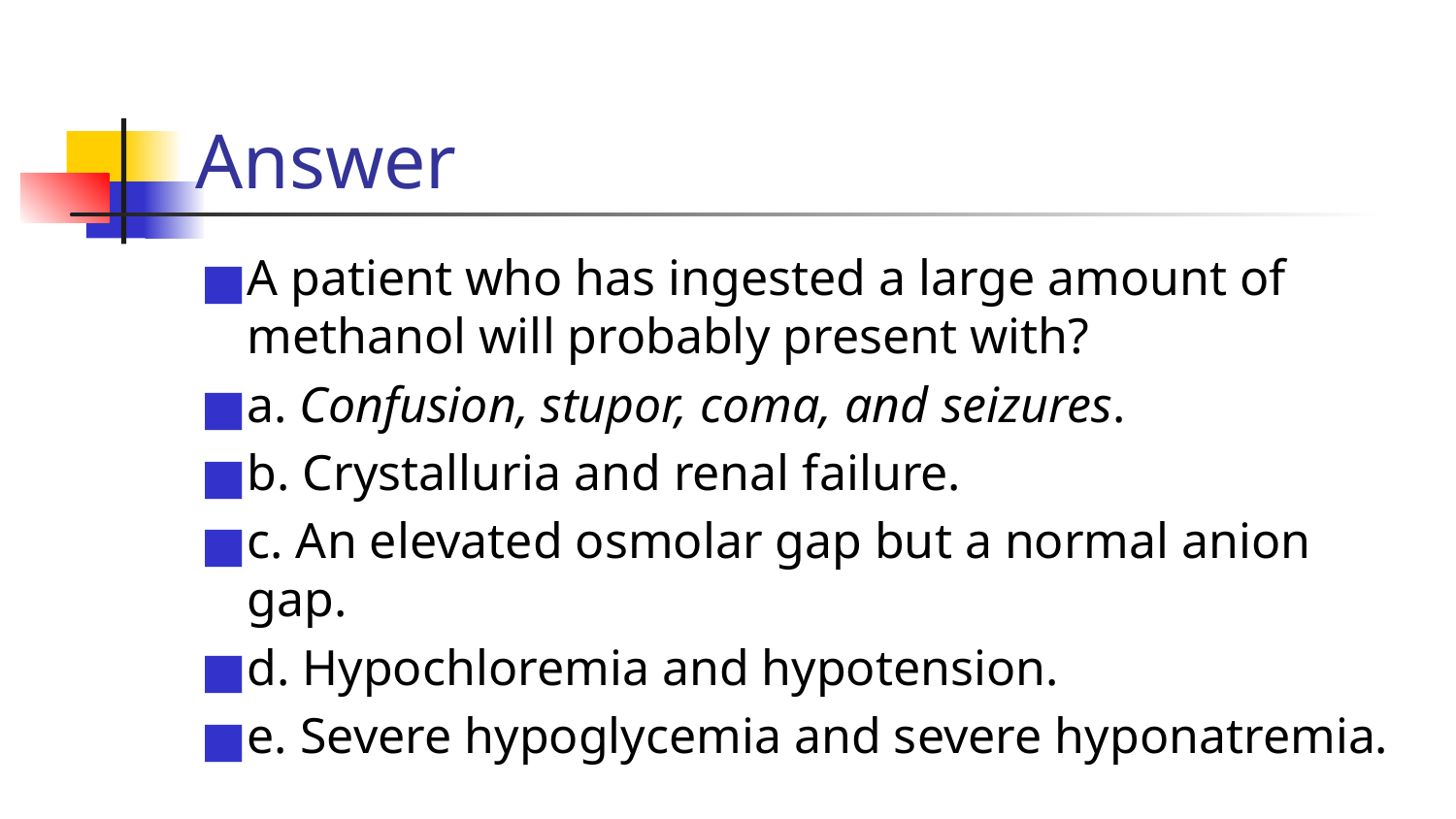

# Answer
A patient who has ingested a large amount of methanol will probably present with?
a. Confusion, stupor, coma, and seizures.
b. Crystalluria and renal failure.
c. An elevated osmolar gap but a normal anion gap.
d. Hypochloremia and hypotension.
e. Severe hypoglycemia and severe hyponatremia.

## Slide 26
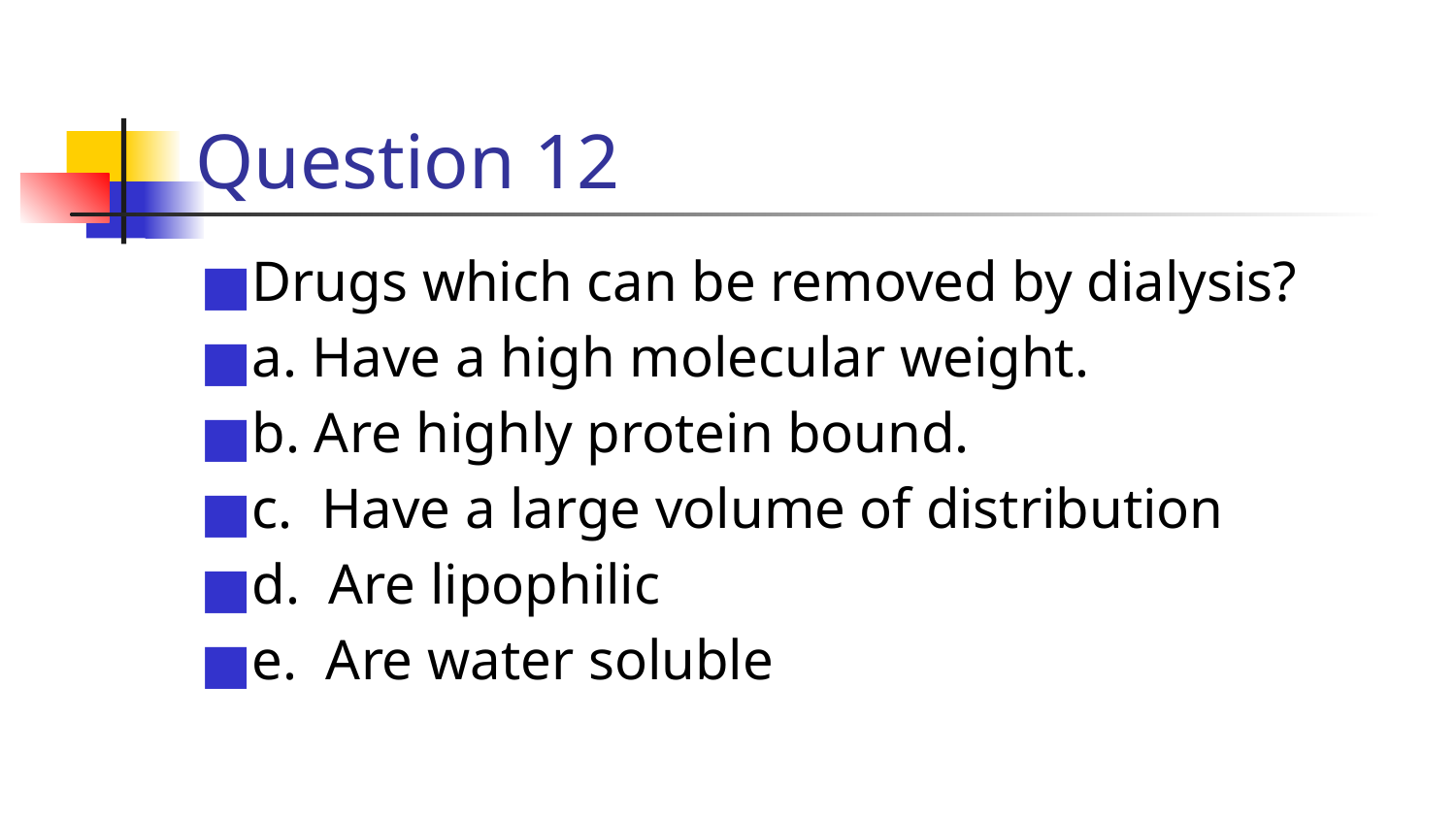

# Question 12
Drugs which can be removed by dialysis?
a. Have a high molecular weight.
b. Are highly protein bound.
c.  Have a large volume of distribution
d.  Are lipophilic
e.  Are water soluble

## Slide 27
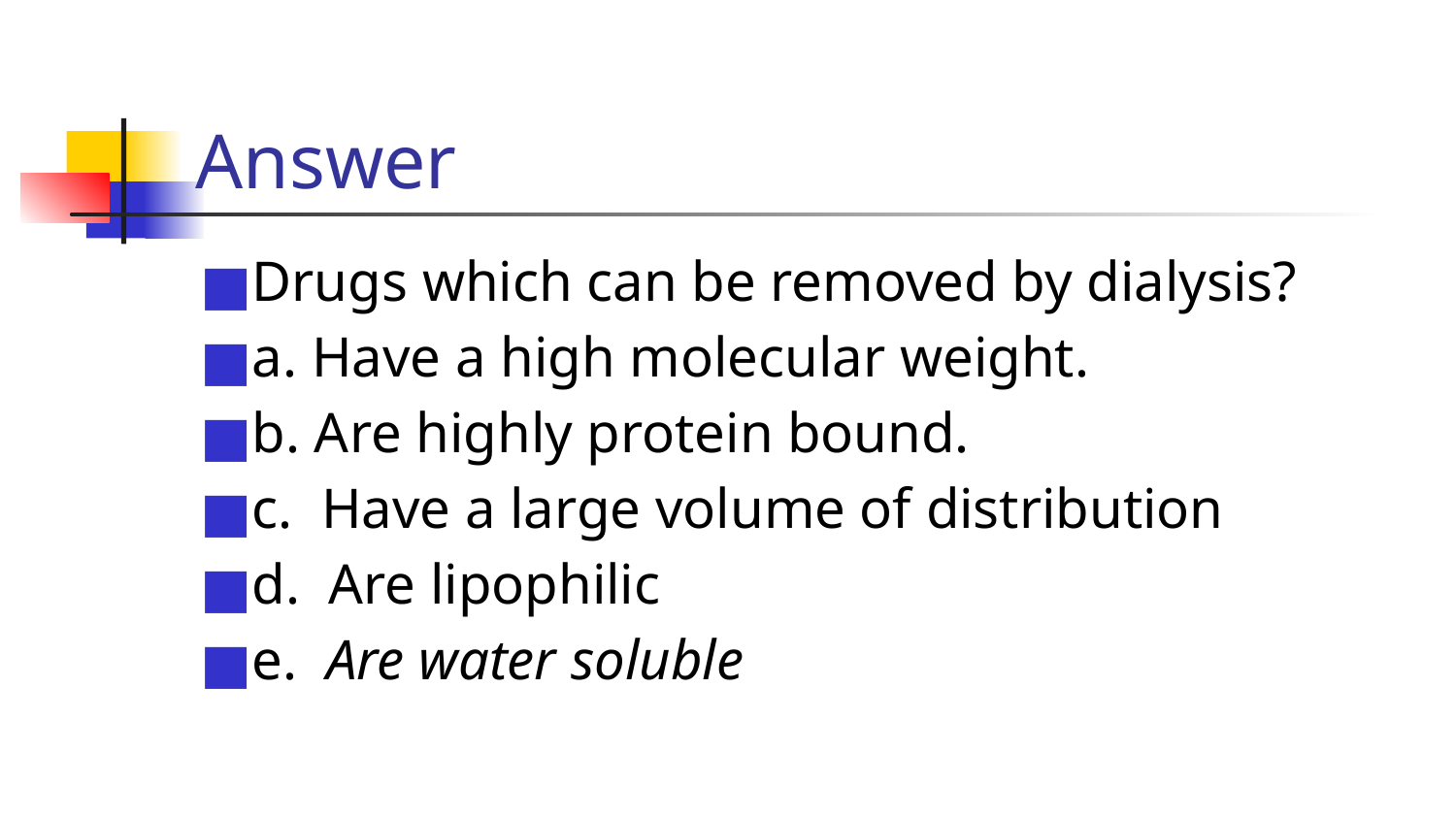

# Answer
Drugs which can be removed by dialysis?
a. Have a high molecular weight.
b. Are highly protein bound.
c.  Have a large volume of distribution
d.  Are lipophilic
e.  Are water soluble

## Slide 28
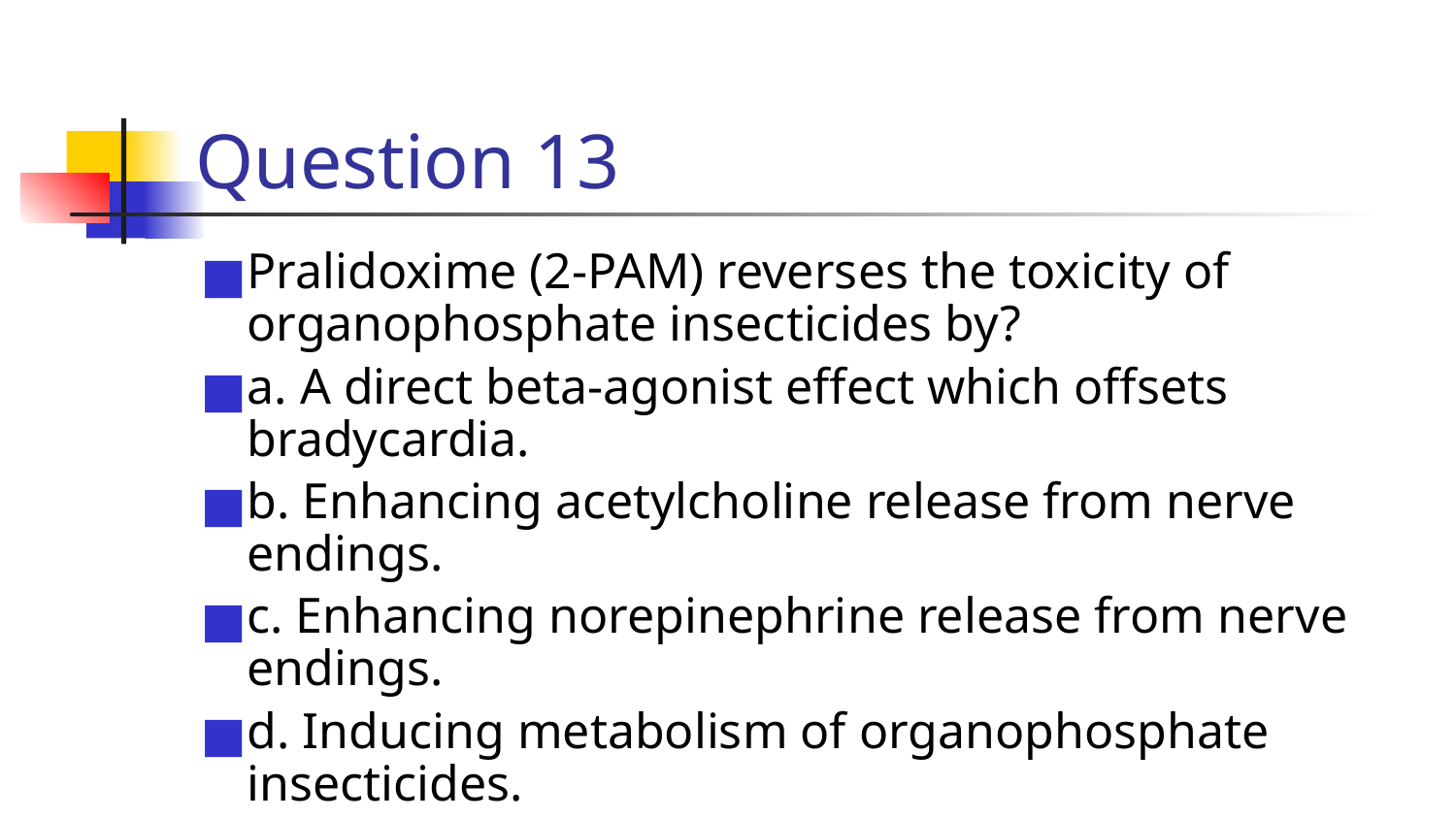

# Question 13
Pralidoxime (2-PAM) reverses the toxicity of organophosphate insecticides by?
a. A direct beta-agonist effect which offsets bradycardia.
b. Enhancing acetylcholine release from nerve endings.
c. Enhancing norepinephrine release from nerve endings.
d. Inducing metabolism of organophosphate insecticides.
e. Reversing the inhibition of acetylcholinesterase.

## Slide 29
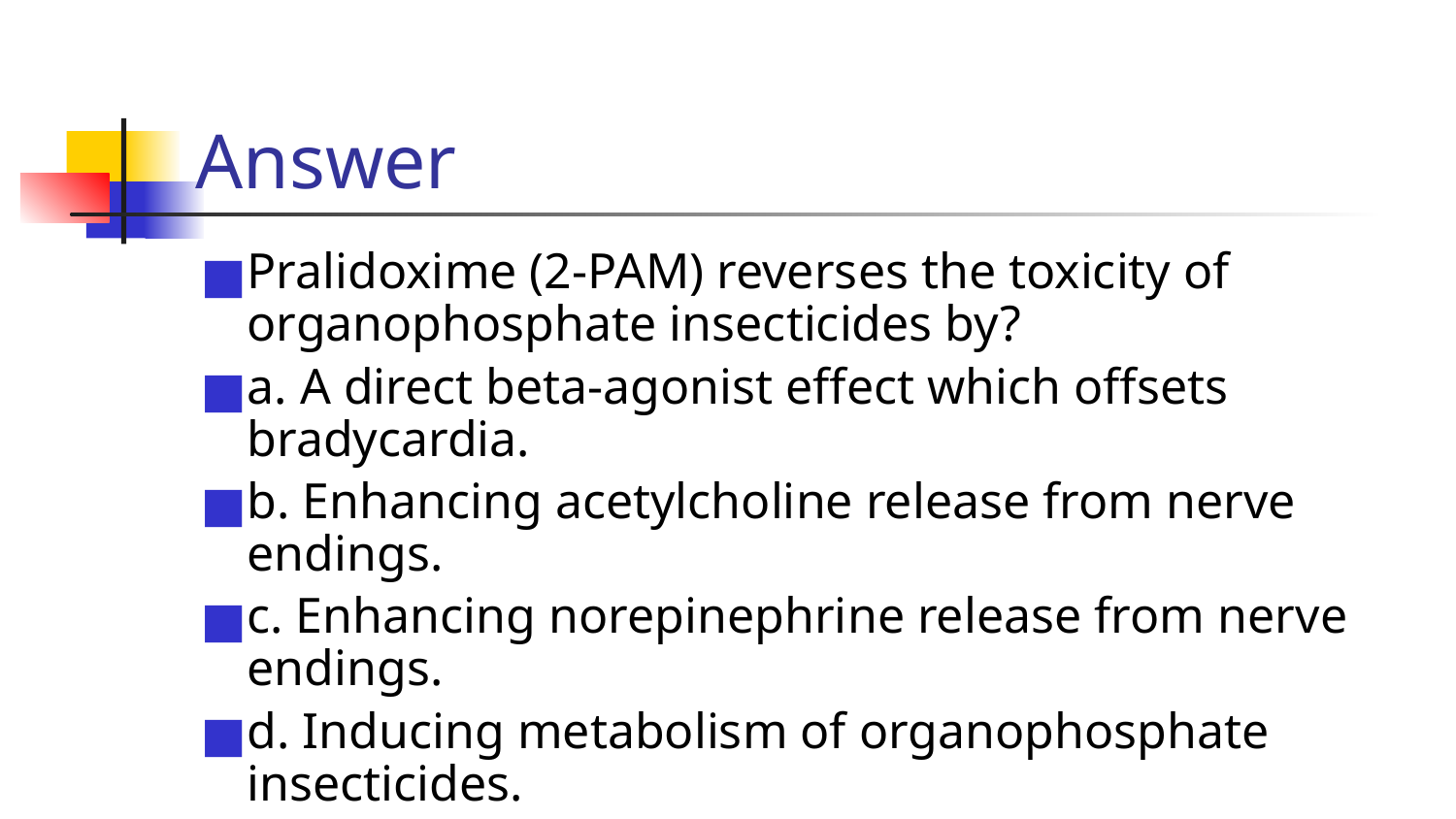

# Answer
Pralidoxime (2-PAM) reverses the toxicity of organophosphate insecticides by?
a. A direct beta-agonist effect which offsets bradycardia.
b. Enhancing acetylcholine release from nerve endings.
c. Enhancing norepinephrine release from nerve endings.
d. Inducing metabolism of organophosphate insecticides.
e. Reversing the inhibition of acetylcholinesterase.

## Slide 30
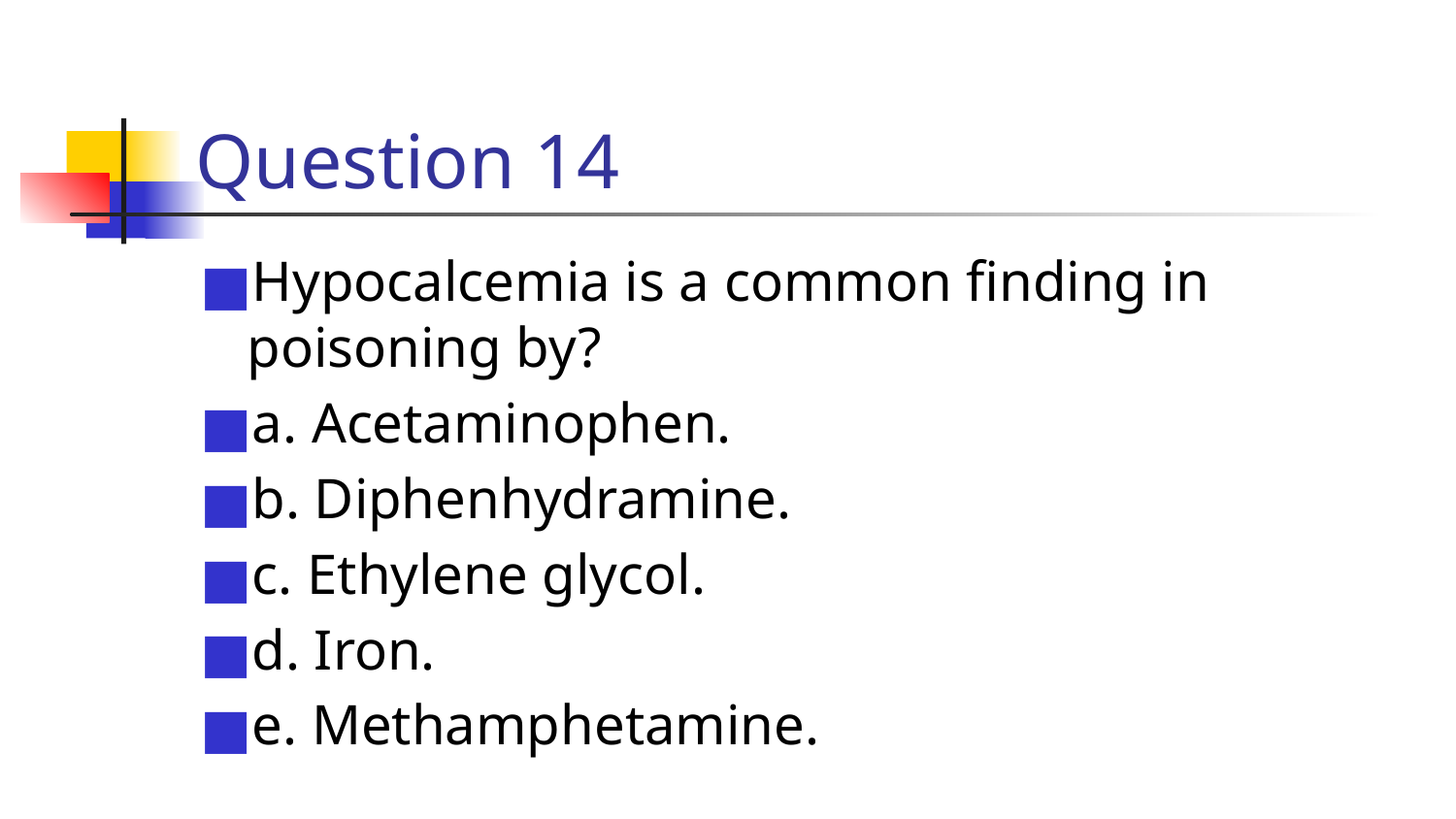

# Question 14
Hypocalcemia is a common finding in poisoning by?
a. Acetaminophen.
b. Diphenhydramine.
c. Ethylene glycol.
d. Iron.
e. Methamphetamine.

## Slide 31
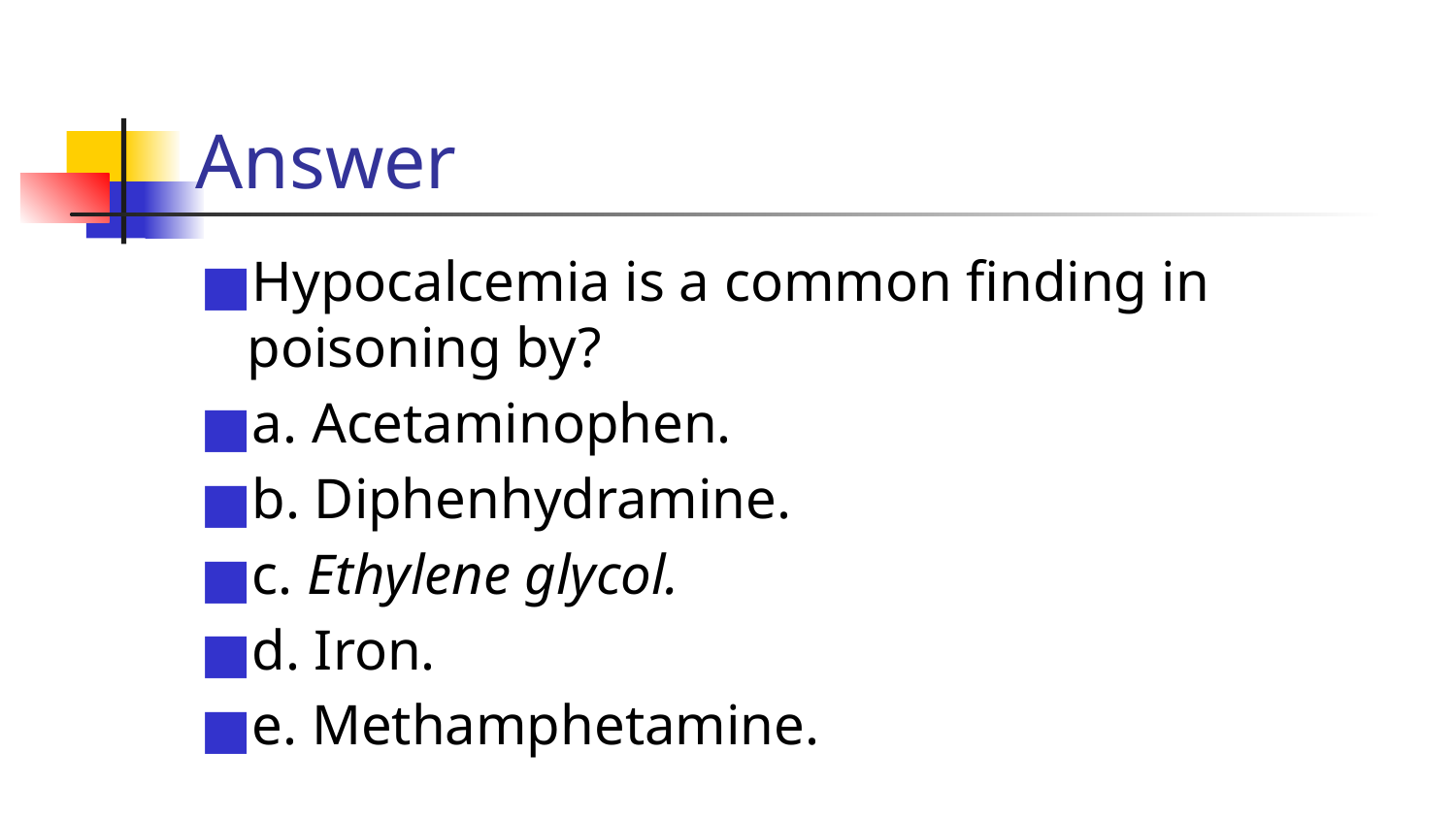

# Answer
Hypocalcemia is a common finding in poisoning by?
a. Acetaminophen.
b. Diphenhydramine.
c. Ethylene glycol.
d. Iron.
e. Methamphetamine.

## Slide 32
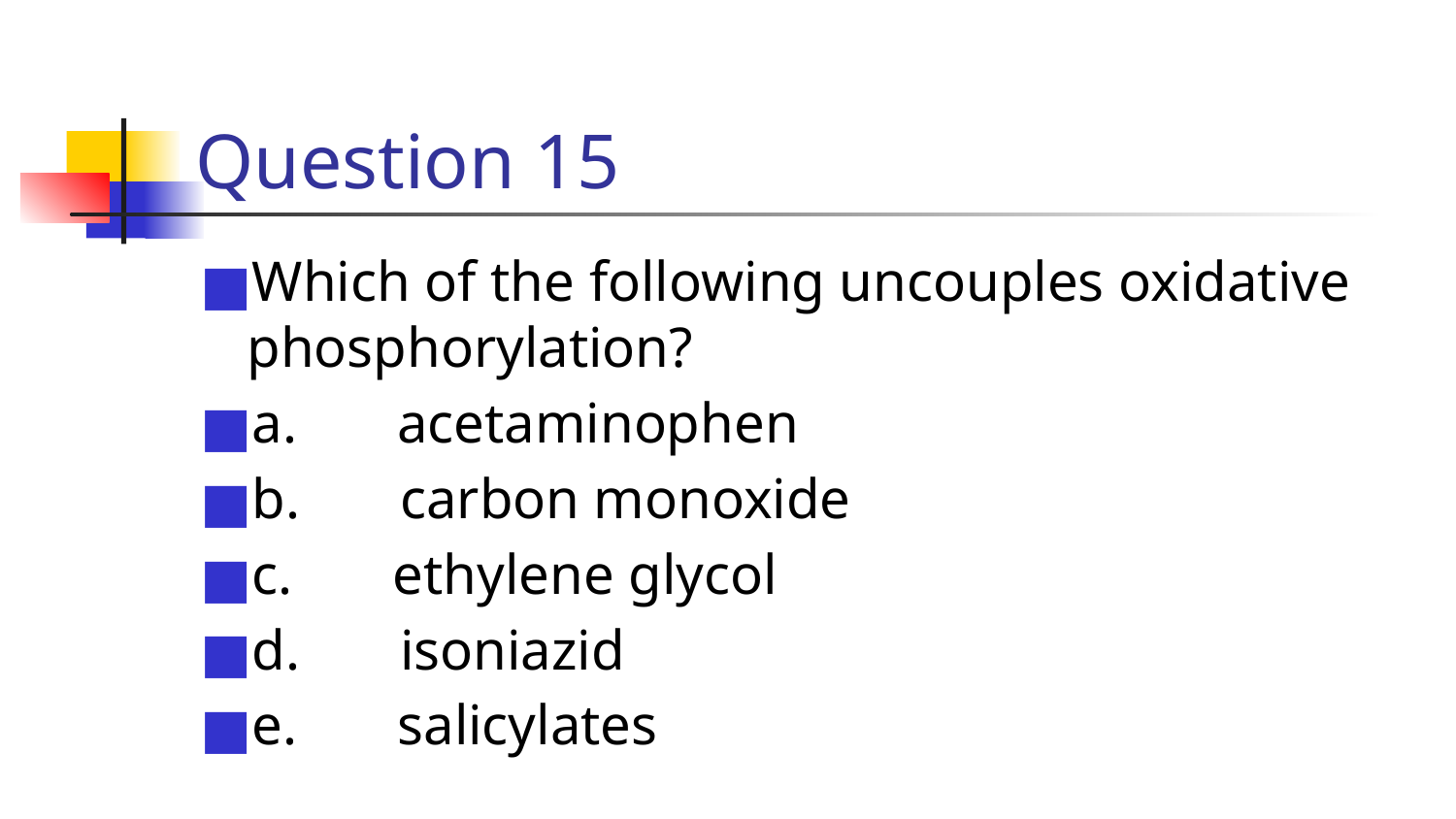

# Question 15
Which of the following uncouples oxidative phosphorylation?
a.       acetaminophen
b.       carbon monoxide
c.       ethylene glycol
d.       isoniazid
e.       salicylates

## Slide 33
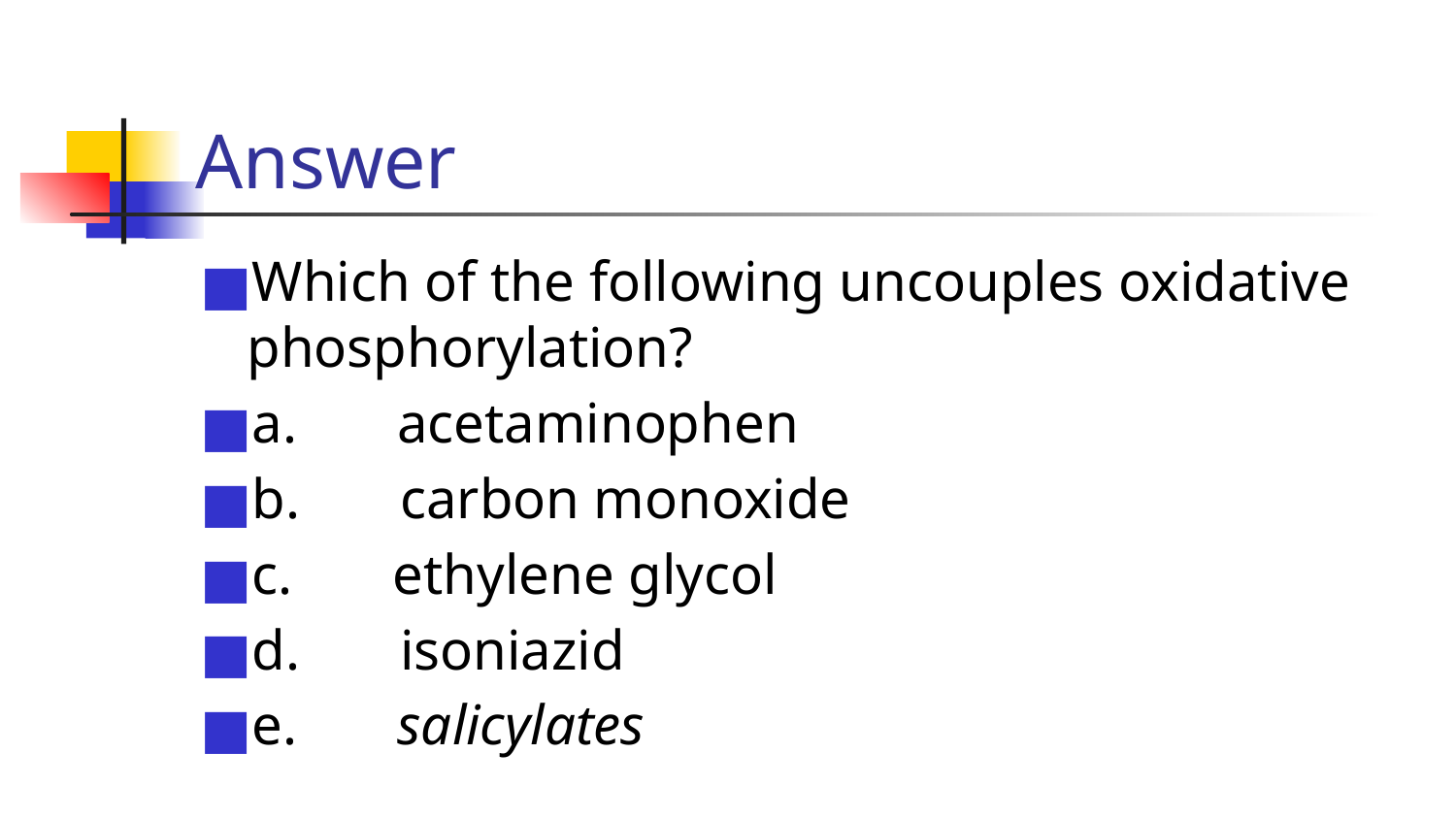

# Answer
Which of the following uncouples oxidative phosphorylation?
a.       acetaminophen
b.       carbon monoxide
c.       ethylene glycol
d.       isoniazid
e.       salicylates

## Slide 34
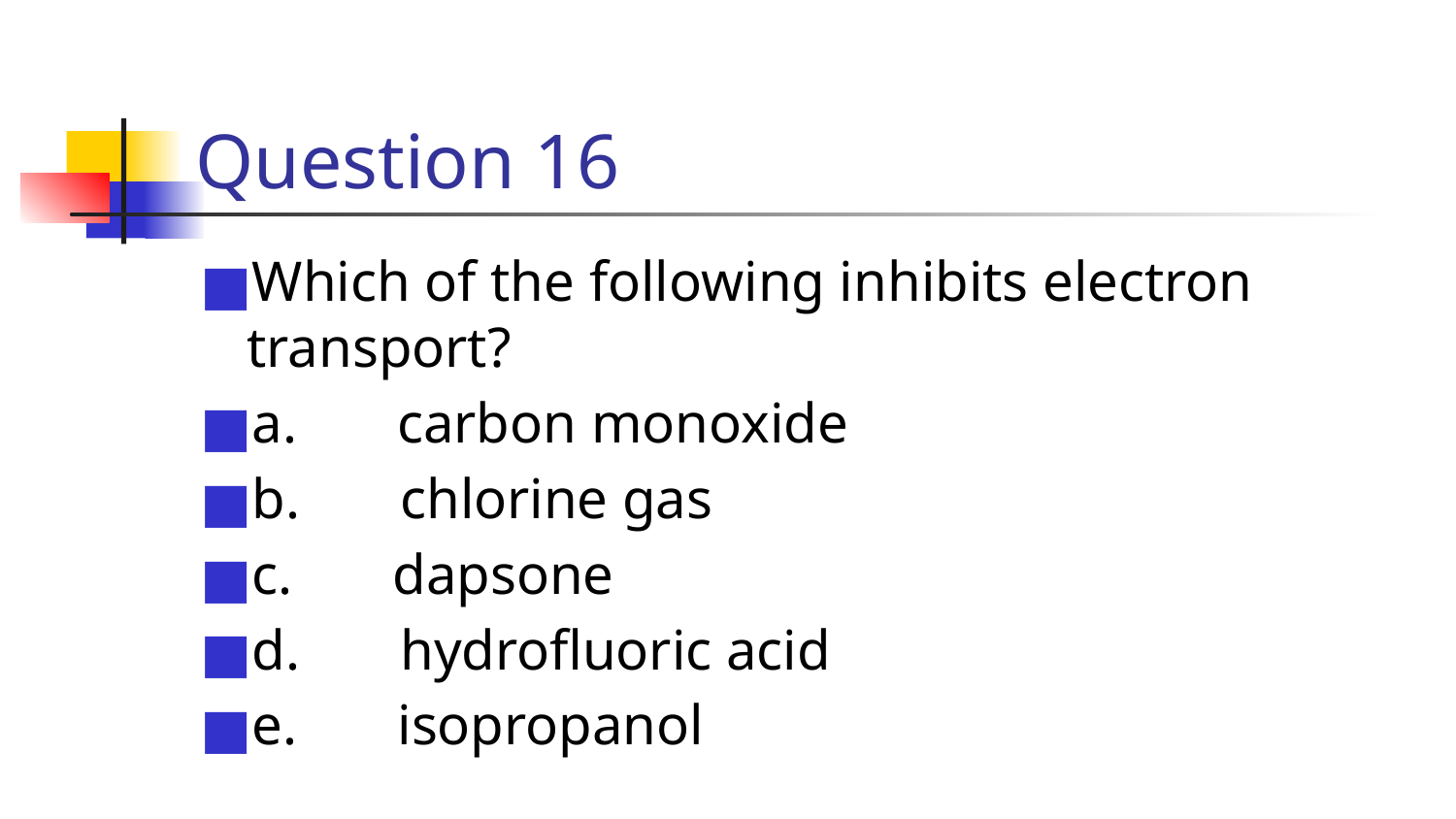

# Question 16
Which of the following inhibits electron transport?
a.       carbon monoxide
b.       chlorine gas
c.       dapsone
d.       hydrofluoric acid
e.       isopropanol

## Slide 35
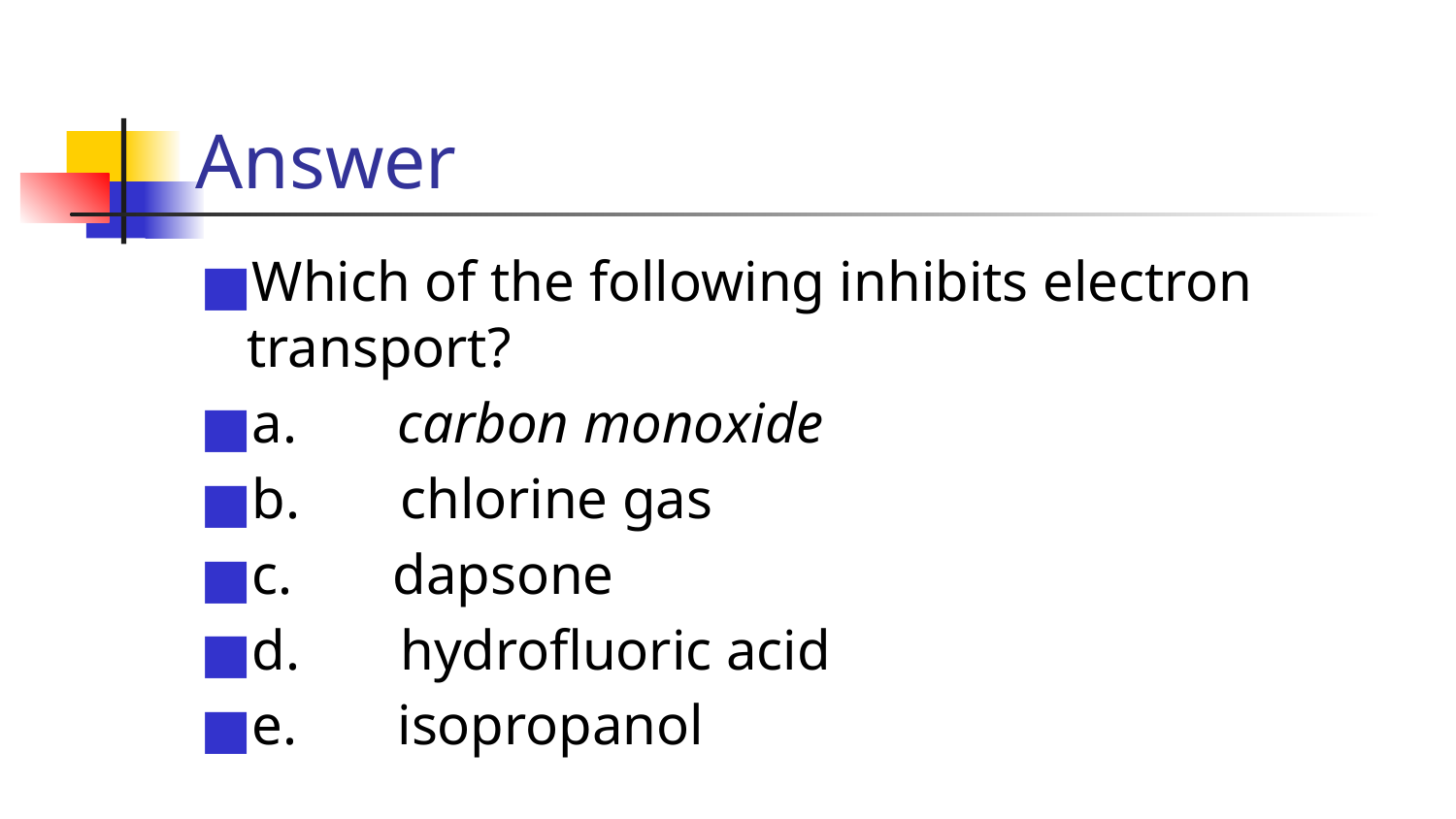

# Answer
Which of the following inhibits electron transport?
a.       carbon monoxide
b.       chlorine gas
c.       dapsone
d.       hydrofluoric acid
e.       isopropanol

## Slide 36
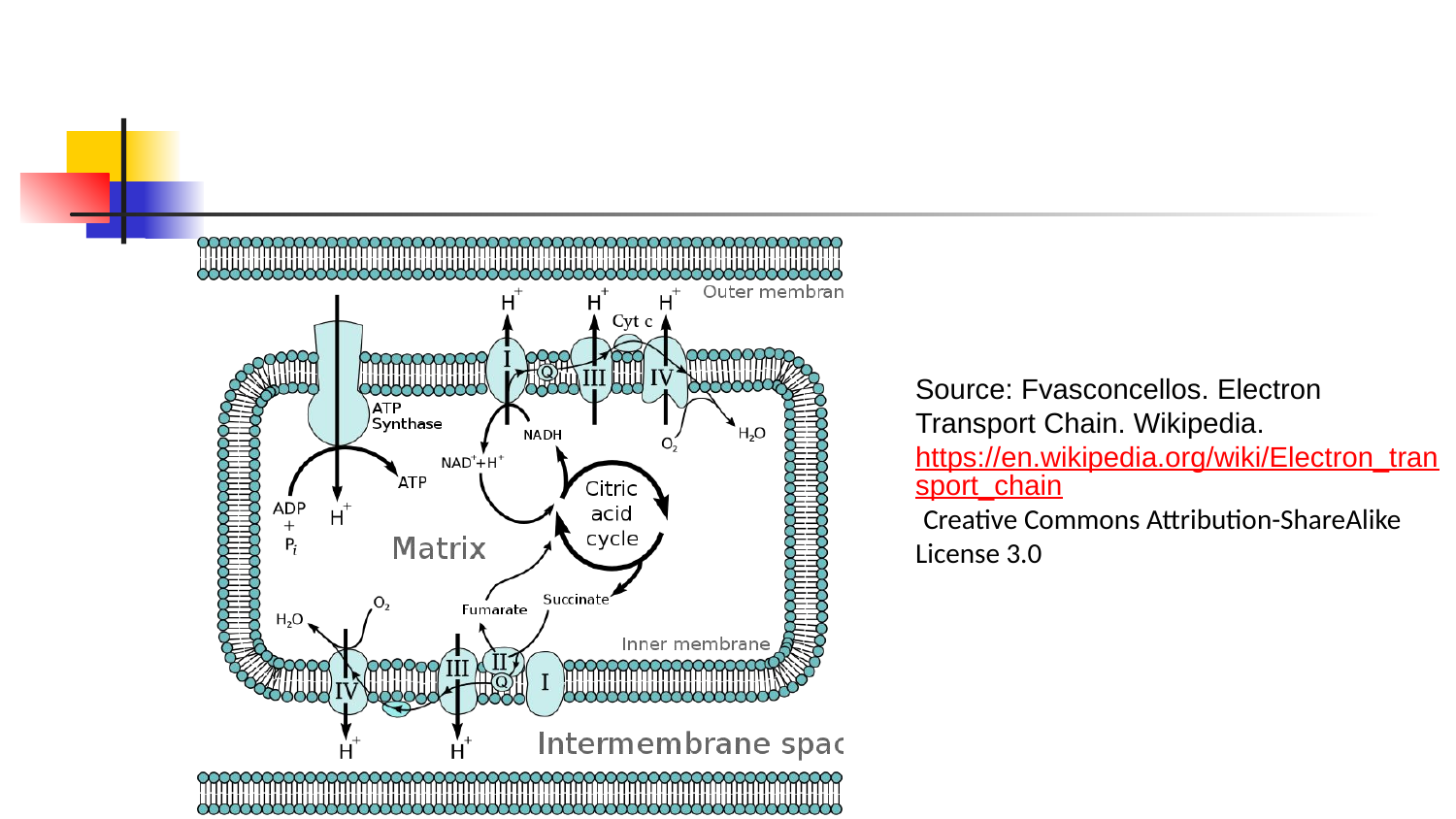

Source: Fvasconcellos. Electron Transport Chain. Wikipedia. https://en.wikipedia.org/wiki/Electron_transport_chain Creative Commons Attribution-ShareAlike License 3.0

## Slide 37
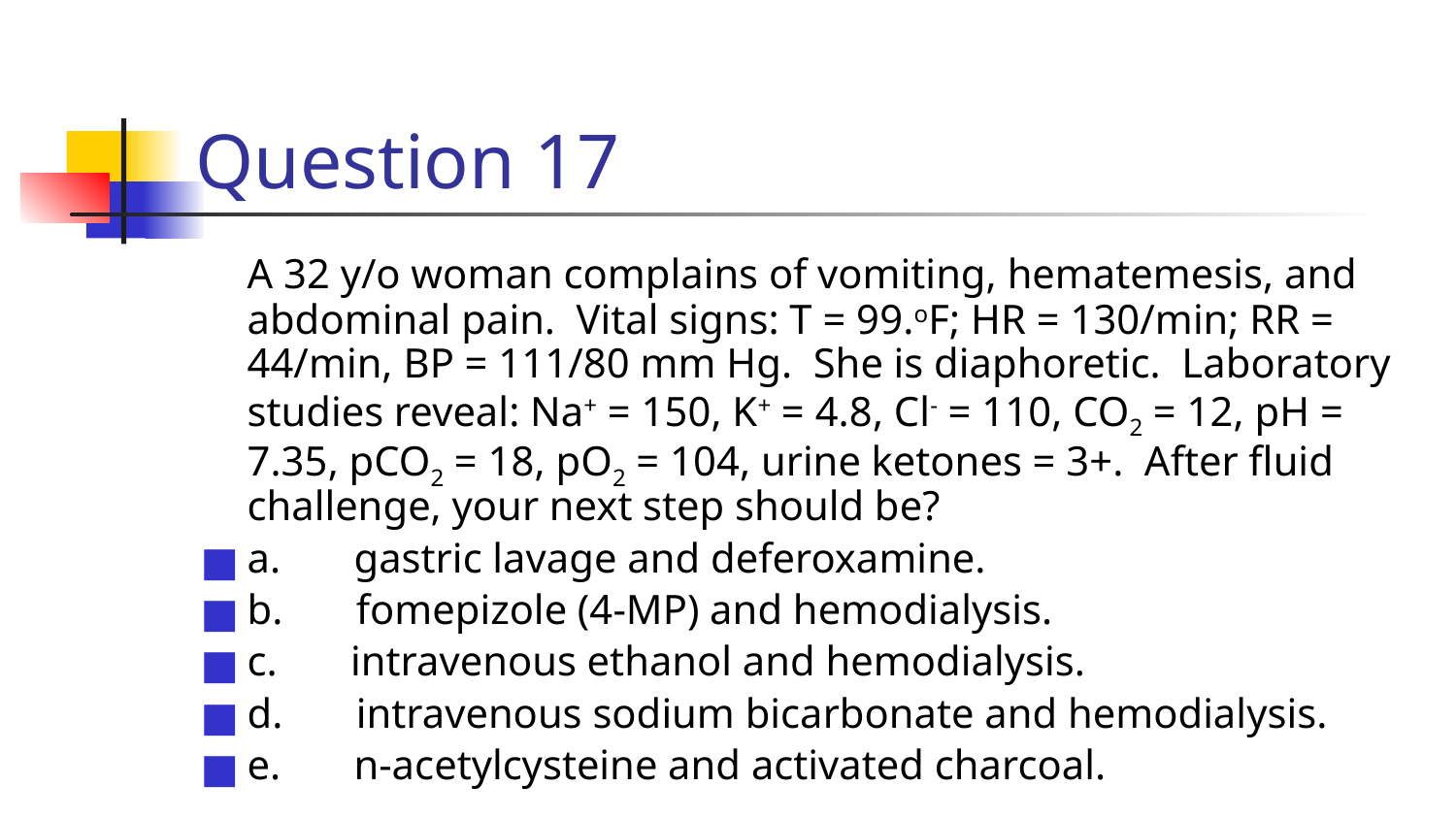

# Question 17
	A 32 y/o woman complains of vomiting, hematemesis, and abdominal pain. Vital signs: T = 99.oF; HR = 130/min; RR = 44/min, BP = 111/80 mm Hg. She is diaphoretic. Laboratory studies reveal: Na+ = 150, K+ = 4.8, Cl- = 110, CO2 = 12, pH = 7.35, pCO2 = 18, pO2 = 104, urine ketones = 3+. After fluid challenge, your next step should be?
a.       gastric lavage and deferoxamine.
b.       fomepizole (4-MP) and hemodialysis.
c.       intravenous ethanol and hemodialysis.
d.       intravenous sodium bicarbonate and hemodialysis.
e.       n-acetylcysteine and activated charcoal.

## Slide 38
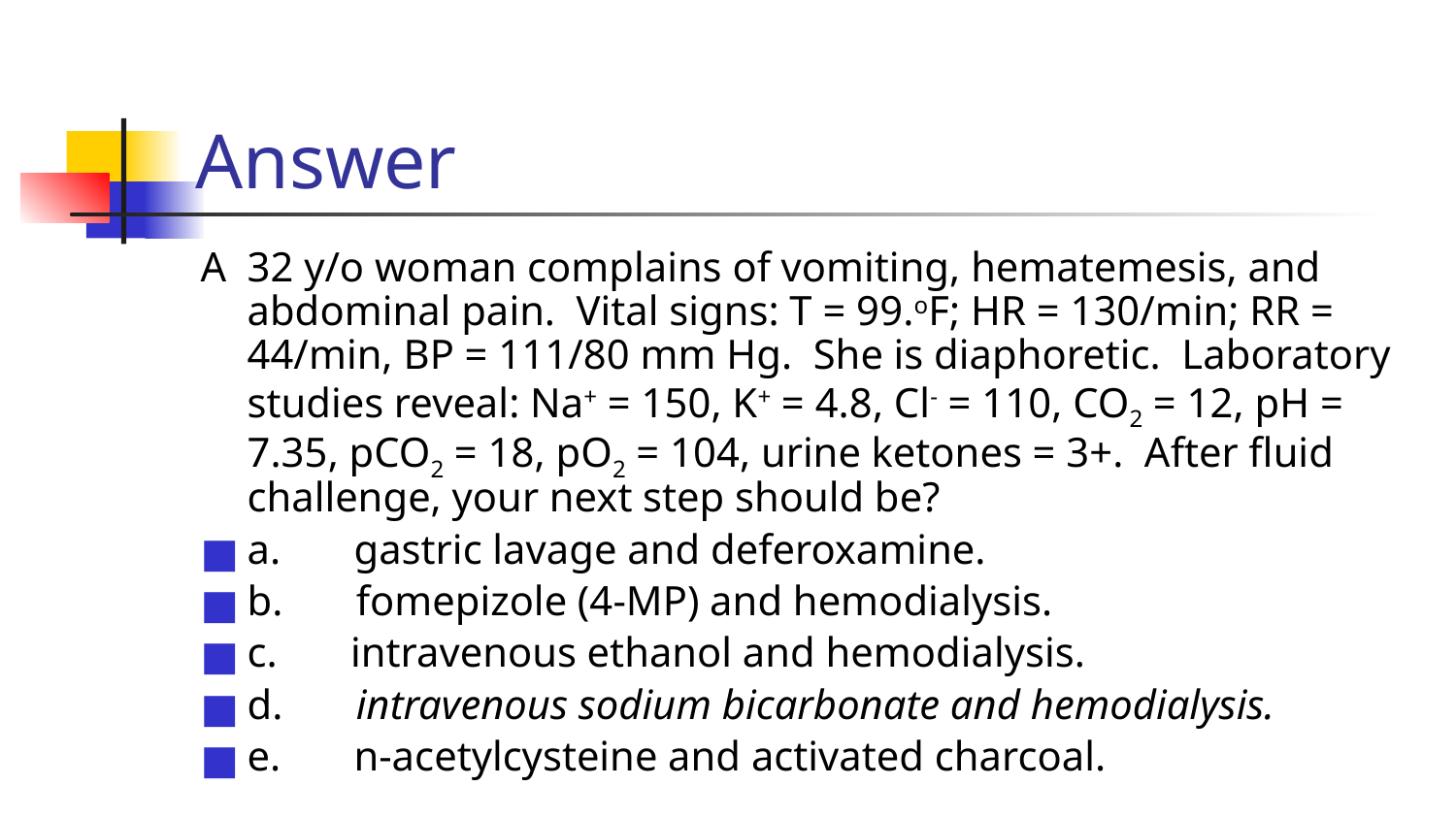

# Answer
A 	32 y/o woman complains of vomiting, hematemesis, and abdominal pain. Vital signs: T = 99.oF; HR = 130/min; RR = 44/min, BP = 111/80 mm Hg. She is diaphoretic. Laboratory studies reveal: Na+ = 150, K+ = 4.8, Cl- = 110, CO2 = 12, pH = 7.35, pCO2 = 18, pO2 = 104, urine ketones = 3+. After fluid challenge, your next step should be?
a.       gastric lavage and deferoxamine.
b.       fomepizole (4-MP) and hemodialysis.
c.       intravenous ethanol and hemodialysis.
d.       intravenous sodium bicarbonate and hemodialysis.
e.       n-acetylcysteine and activated charcoal.

## Slide 39
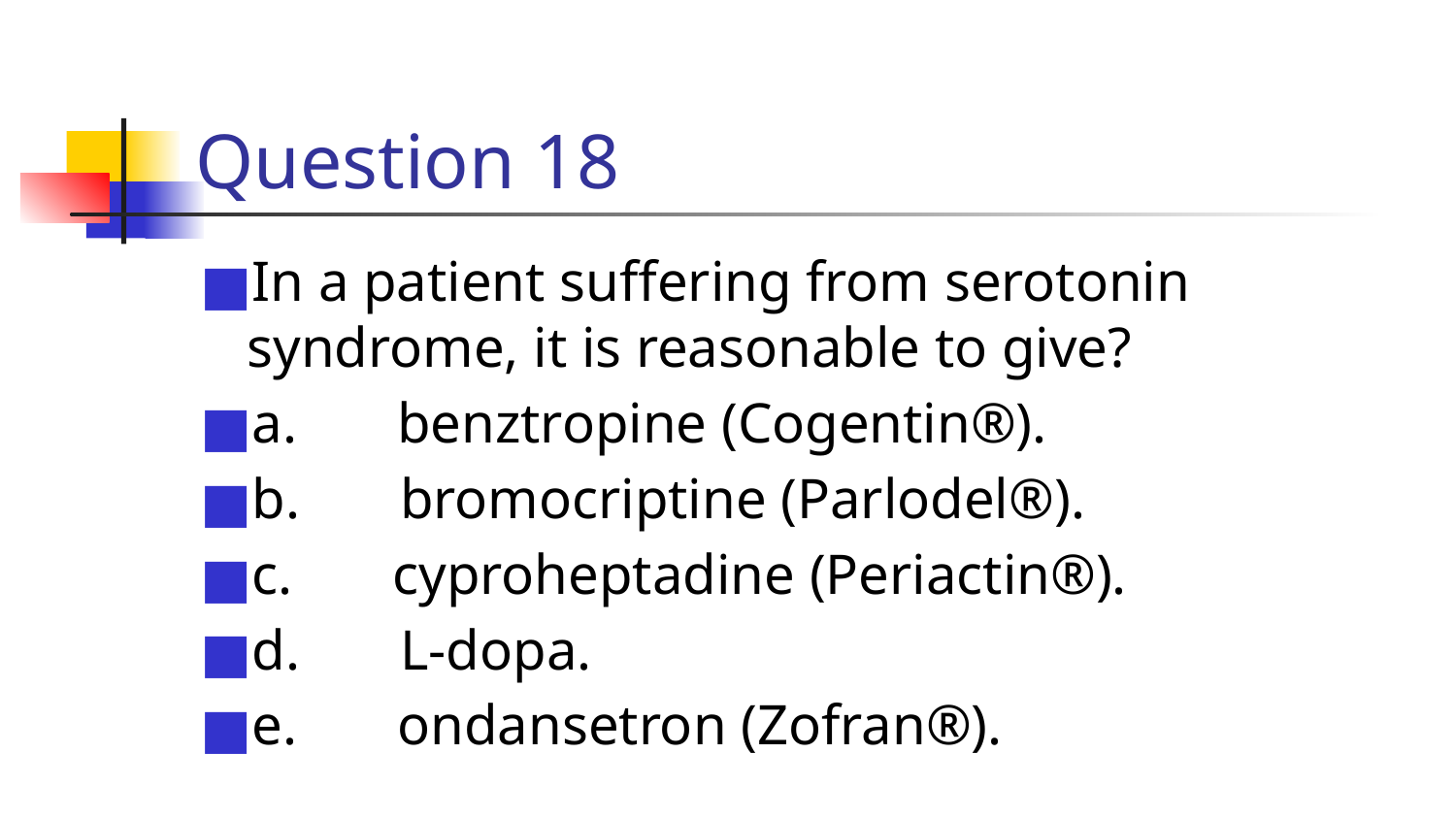

# Question 18
In a patient suffering from serotonin syndrome, it is reasonable to give?
a.       benztropine (Cogentin®).
b.       bromocriptine (Parlodel®).
c.       cyproheptadine (Periactin®).
d.       L-dopa.
e.       ondansetron (Zofran®).

## Slide 40
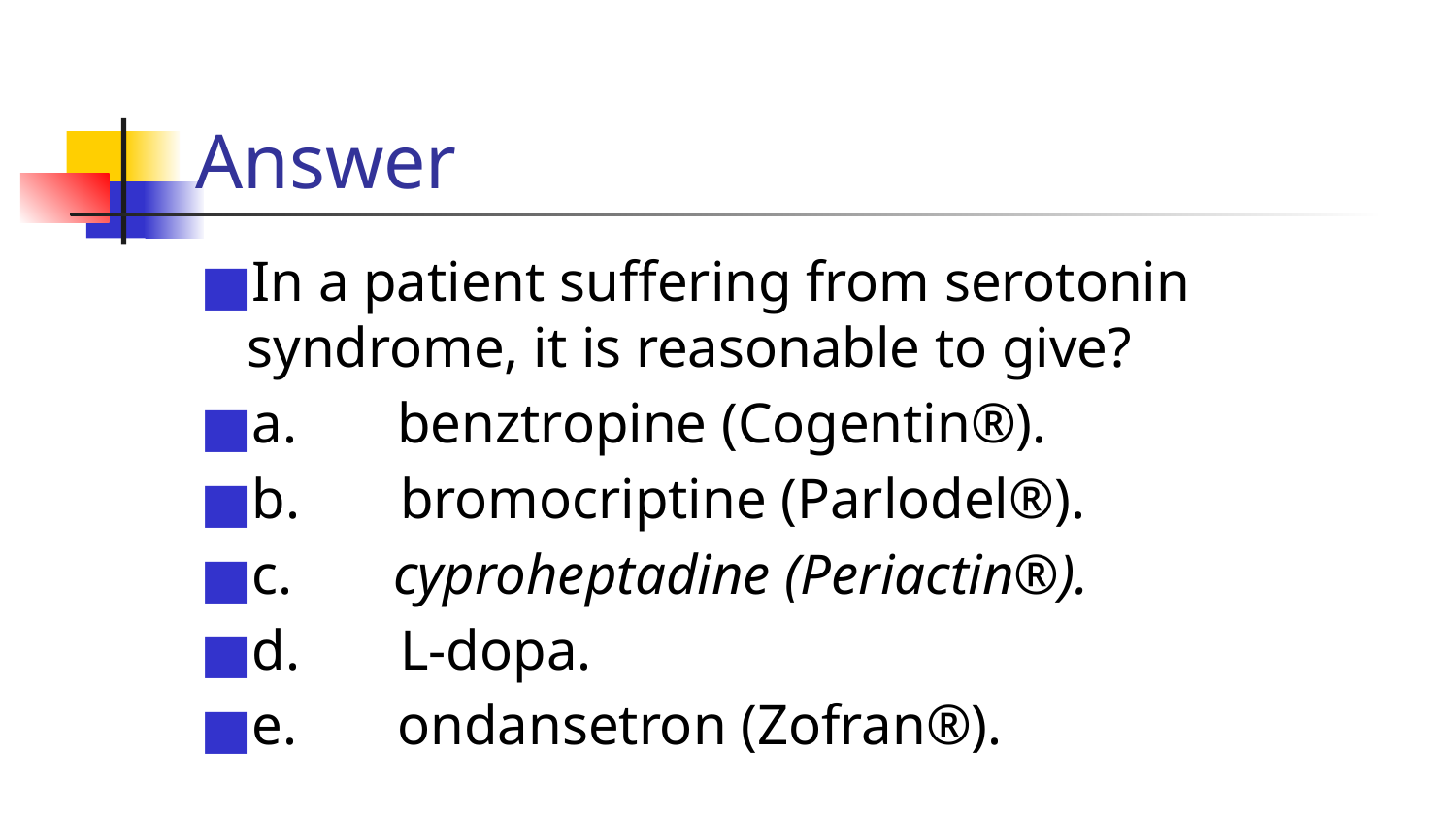

# Answer
In a patient suffering from serotonin syndrome, it is reasonable to give?
a.       benztropine (Cogentin®).
b.       bromocriptine (Parlodel®).
c.       cyproheptadine (Periactin®).
d.       L-dopa.
e.       ondansetron (Zofran®).

## Slide 41
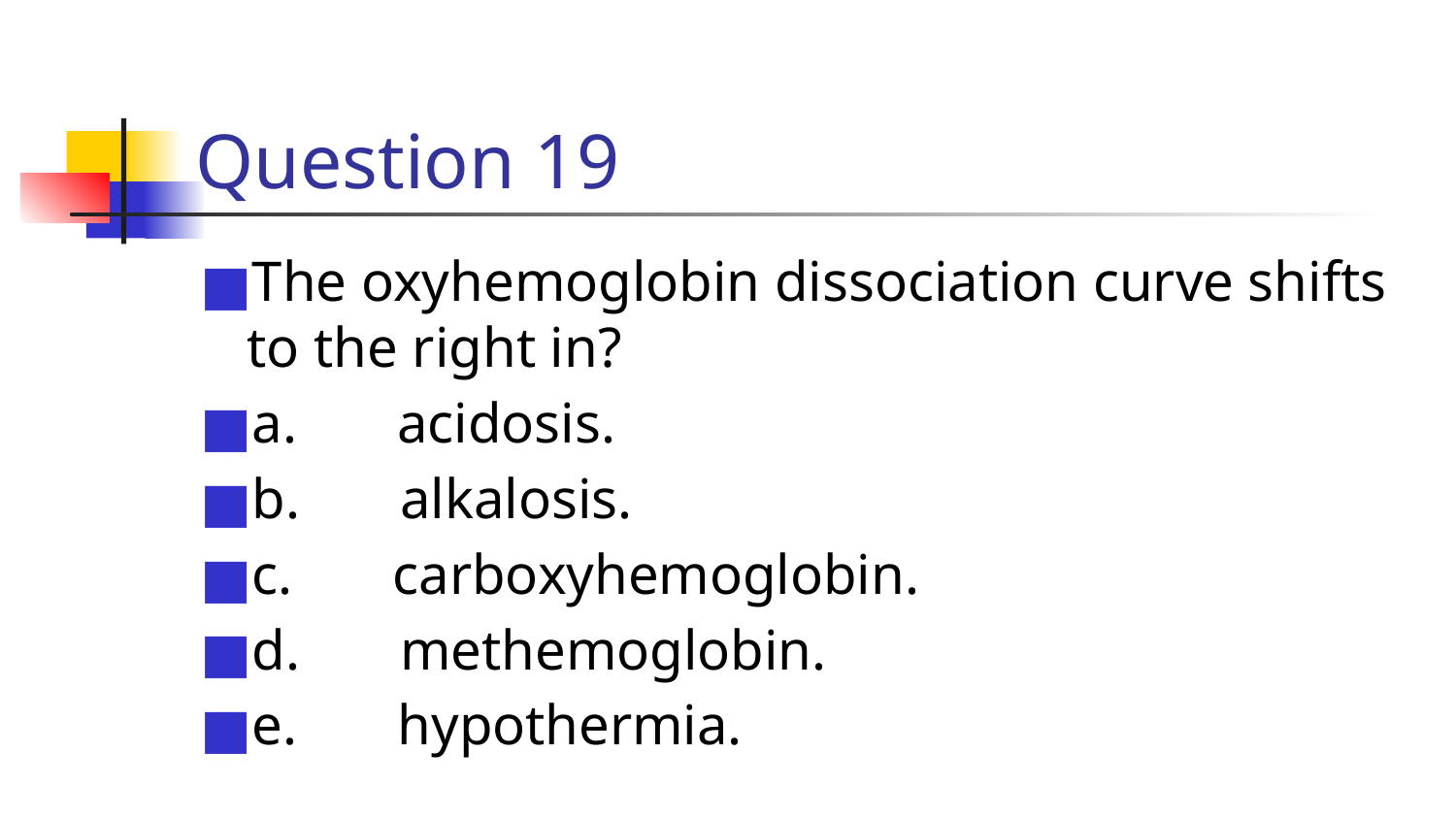

# Question 19
The oxyhemoglobin dissociation curve shifts to the right in?
a.       acidosis.
b.       alkalosis.
c.       carboxyhemoglobin.
d.       methemoglobin.
e.       hypothermia.

## Slide 42
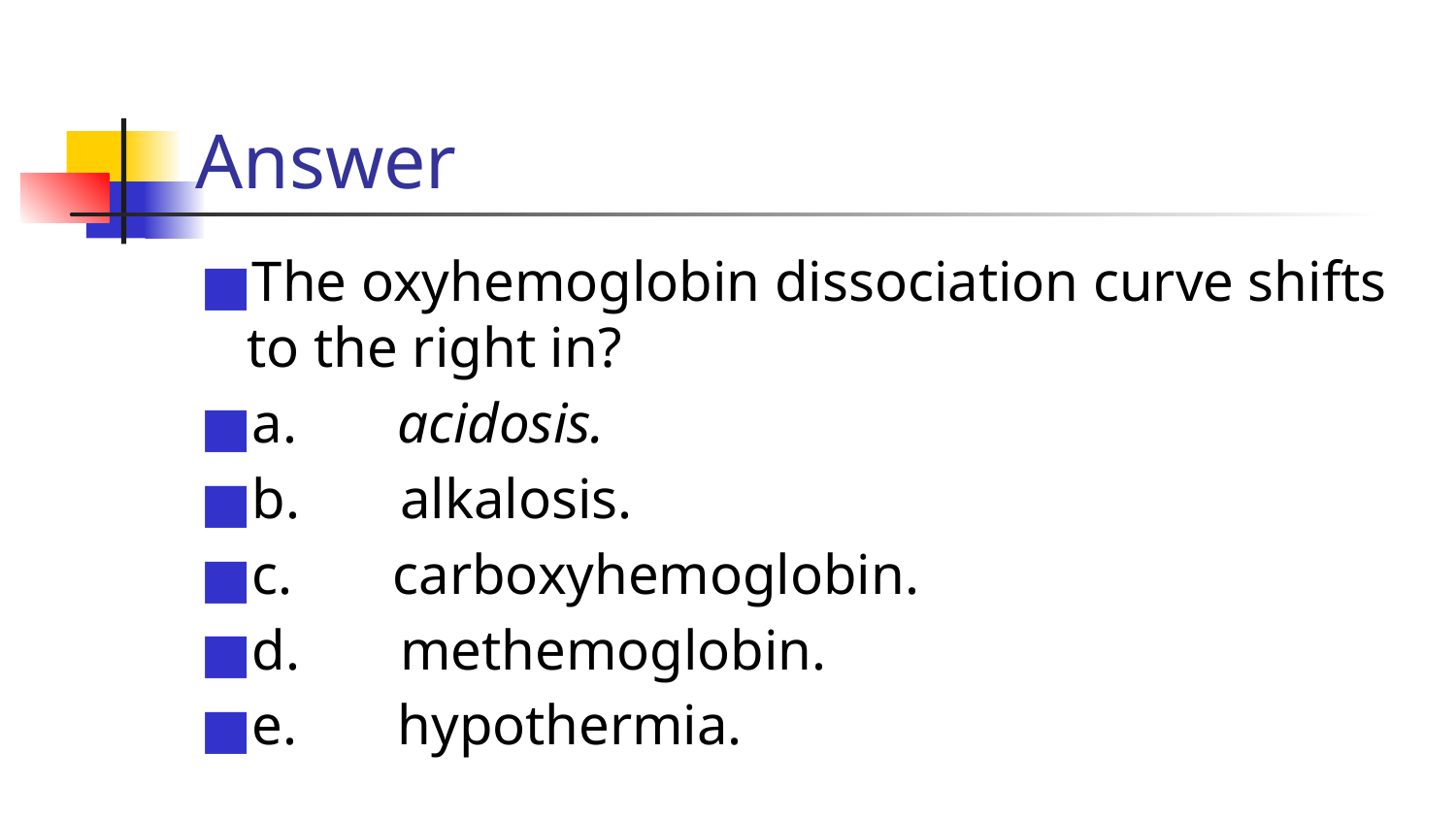

# Answer
The oxyhemoglobin dissociation curve shifts to the right in?
a.       acidosis.
b.       alkalosis.
c.       carboxyhemoglobin.
d.       methemoglobin.
e.       hypothermia.

## Slide 43
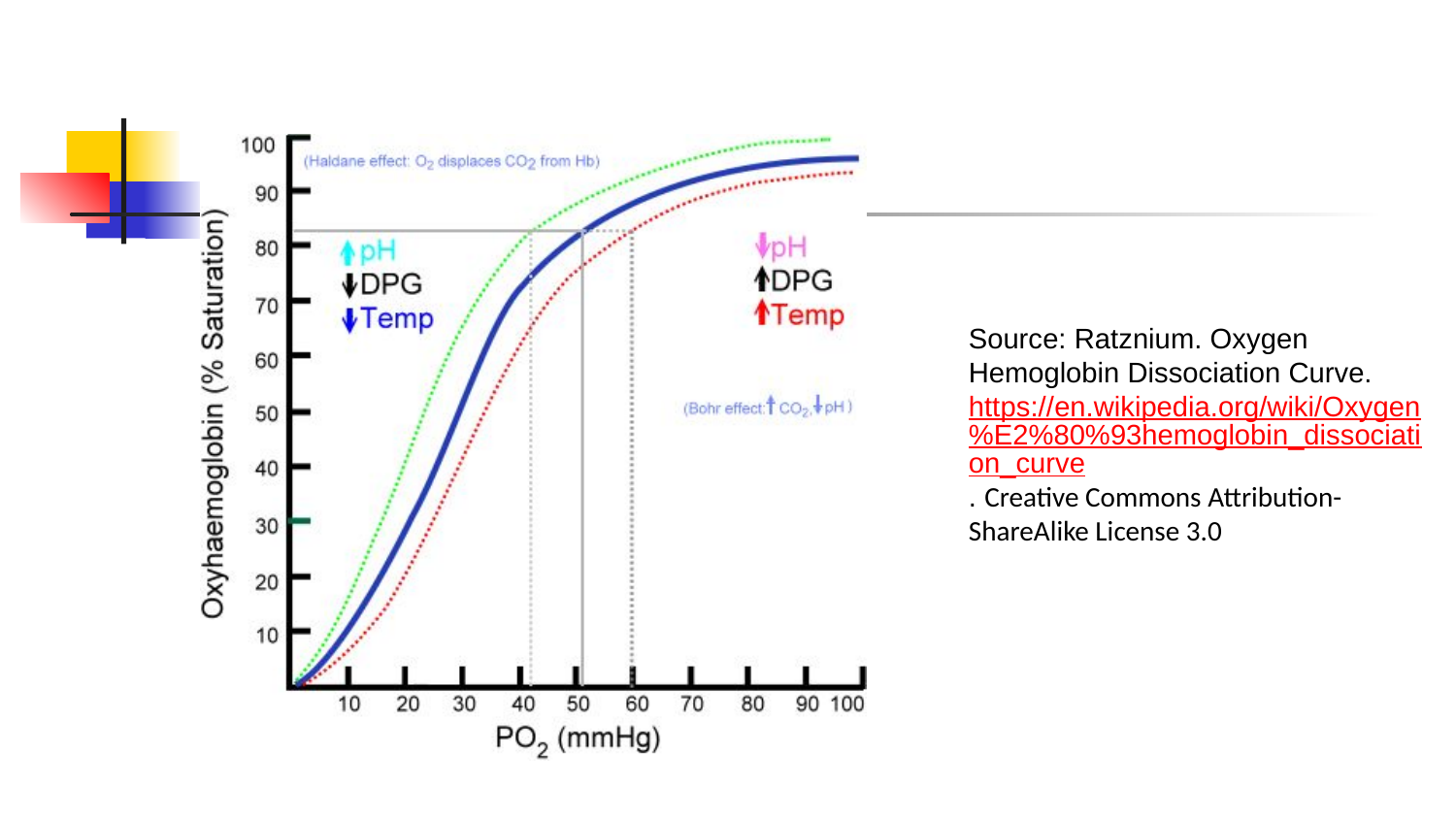

Source: Ratznium. Oxygen Hemoglobin Dissociation Curve. https://en.wikipedia.org/wiki/Oxygen%E2%80%93hemoglobin_dissociation_curve. Creative Commons Attribution-ShareAlike License 3.0

## Slide 44
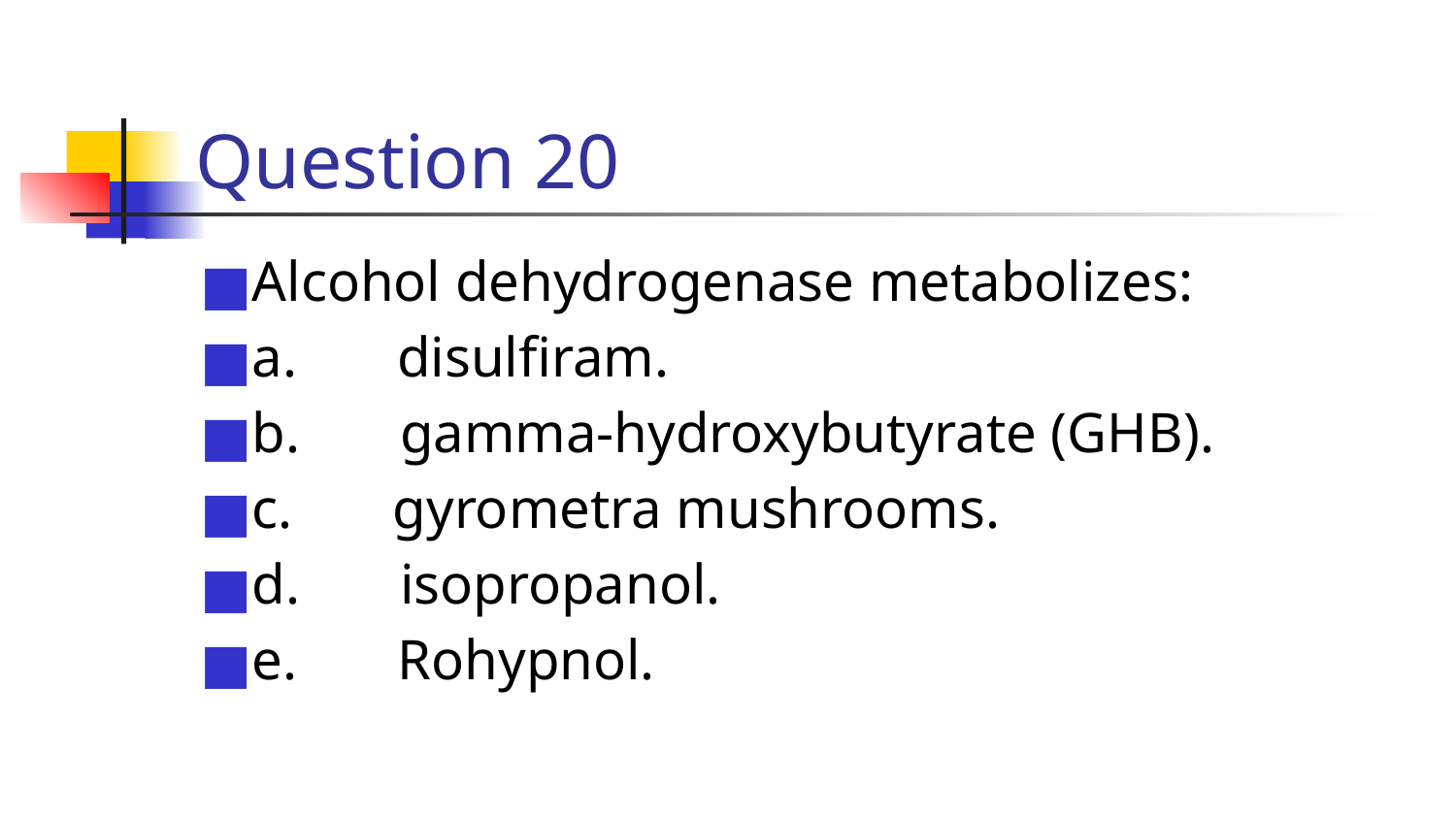

# Question 20
Alcohol dehydrogenase metabolizes:
a.       disulfiram.
b.       gamma-hydroxybutyrate (GHB).
c.       gyrometra mushrooms.
d.       isopropanol.
e.       Rohypnol.

## Slide 45
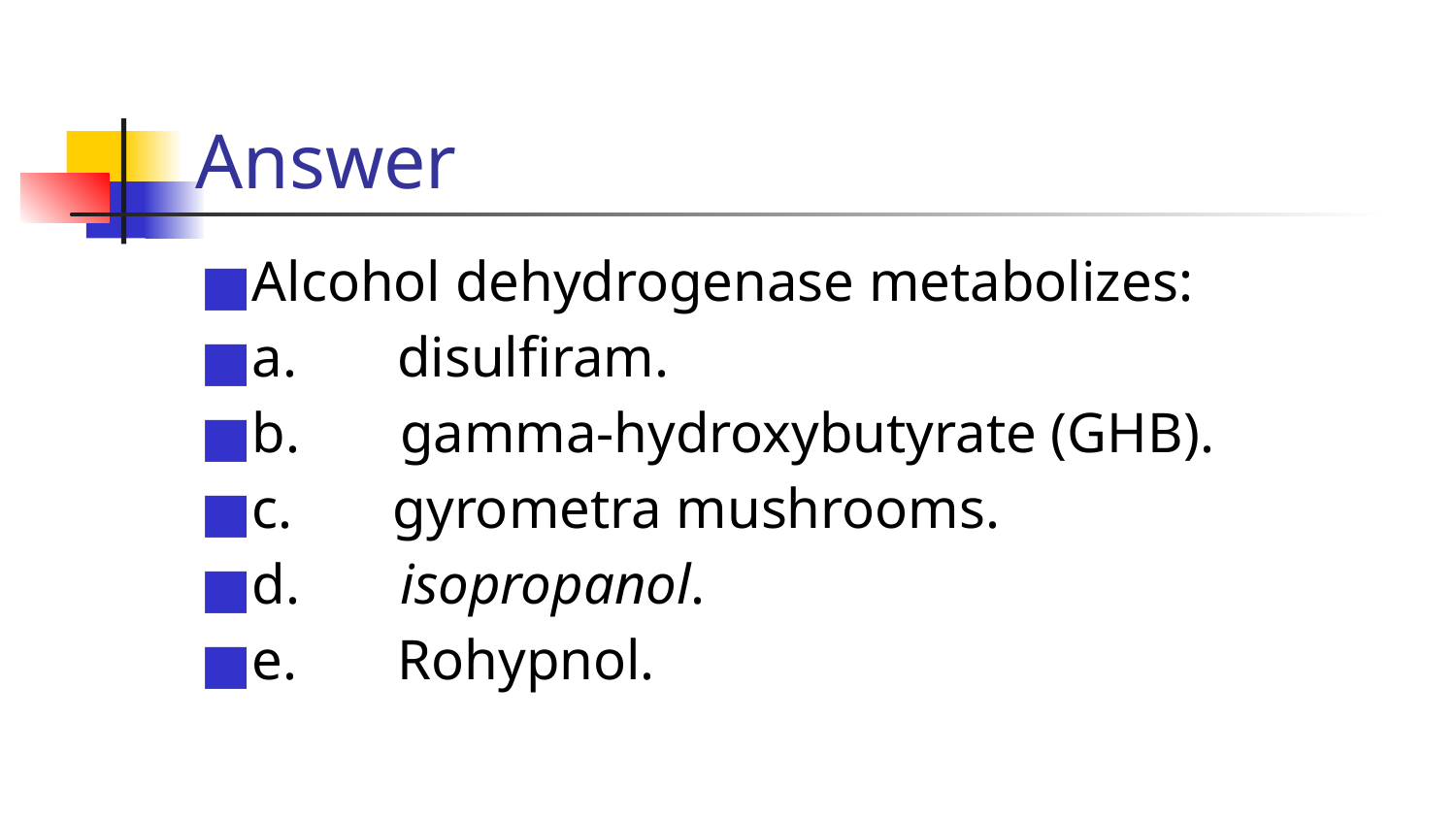

# Answer
Alcohol dehydrogenase metabolizes:
a.       disulfiram.
b.       gamma-hydroxybutyrate (GHB).
c.       gyrometra mushrooms.
d.       isopropanol.
e.       Rohypnol.

## Slide 46
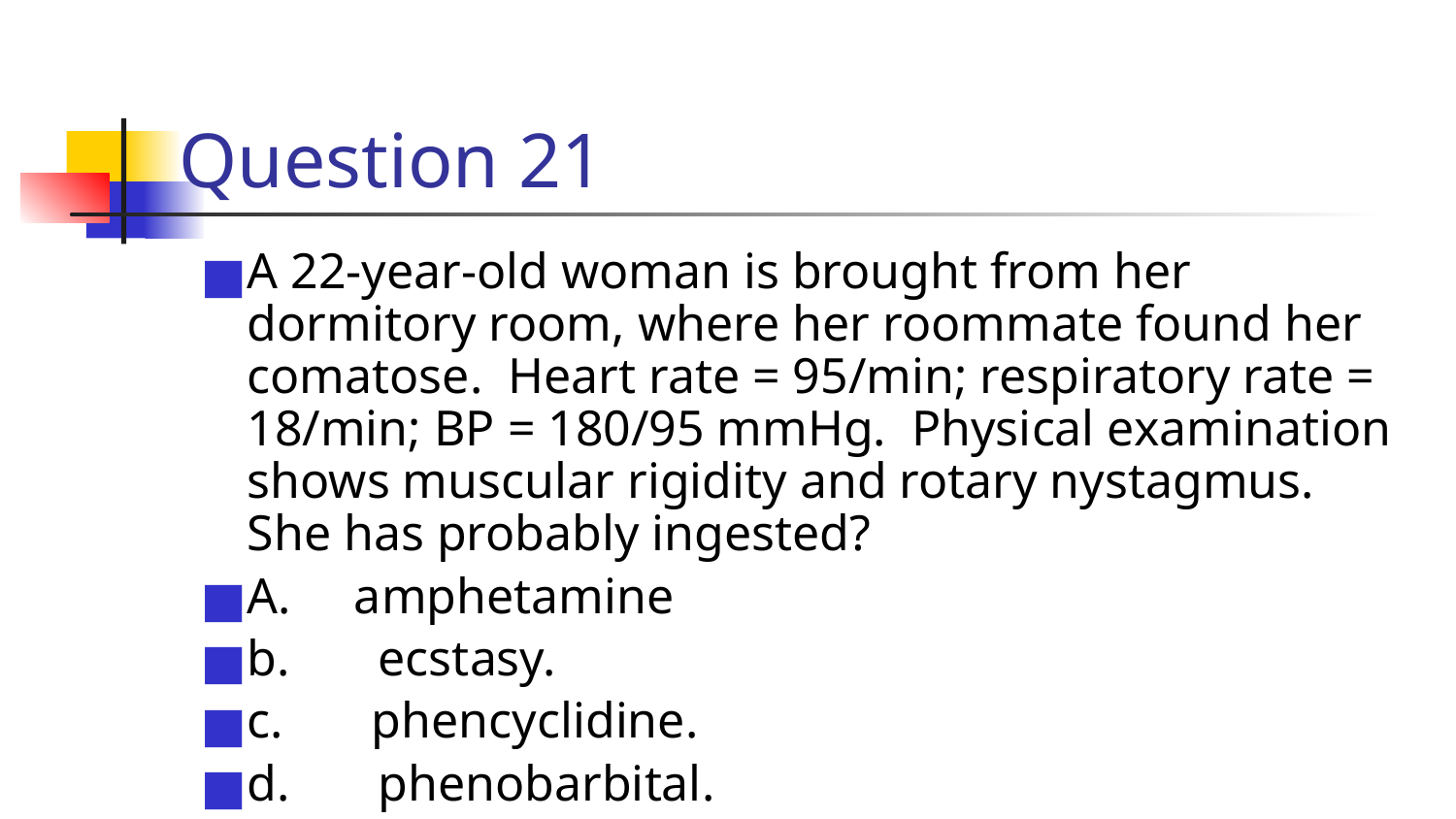

# Question 21
A 22-year-old woman is brought from her dormitory room, where her roommate found her comatose. Heart rate = 95/min; respiratory rate = 18/min; BP = 180/95 mmHg. Physical examination shows muscular rigidity and rotary nystagmus. She has probably ingested?
A. amphetamine
b.       ecstasy.
c.       phencyclidine.
d.       phenobarbital.
e.       diazepam.

## Slide 47
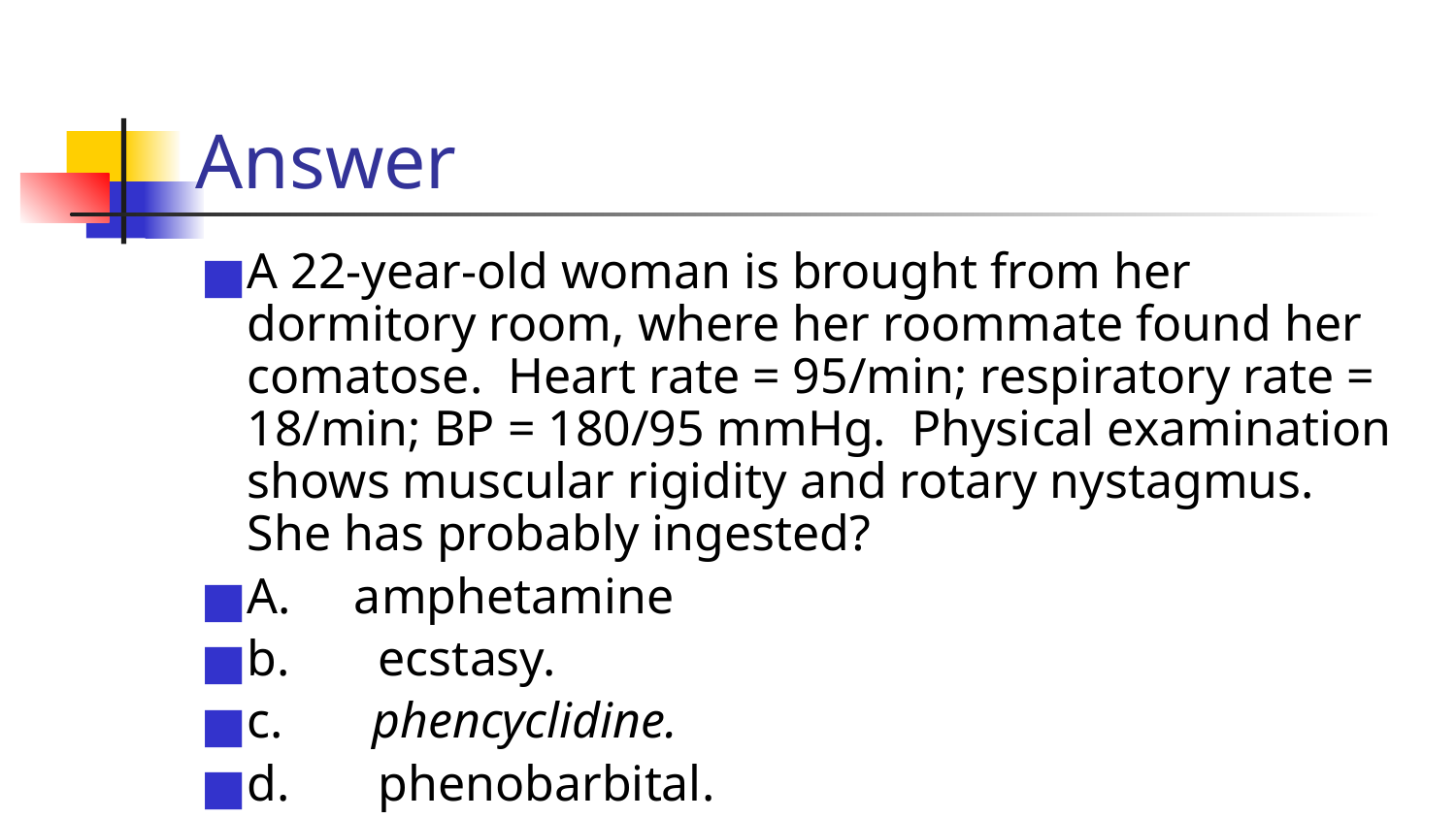

# Answer
A 22-year-old woman is brought from her dormitory room, where her roommate found her comatose. Heart rate = 95/min; respiratory rate = 18/min; BP = 180/95 mmHg. Physical examination shows muscular rigidity and rotary nystagmus. She has probably ingested?
A. amphetamine
b.       ecstasy.
c.       phencyclidine.
d.       phenobarbital.
e.       diazepam.

## Slide 48
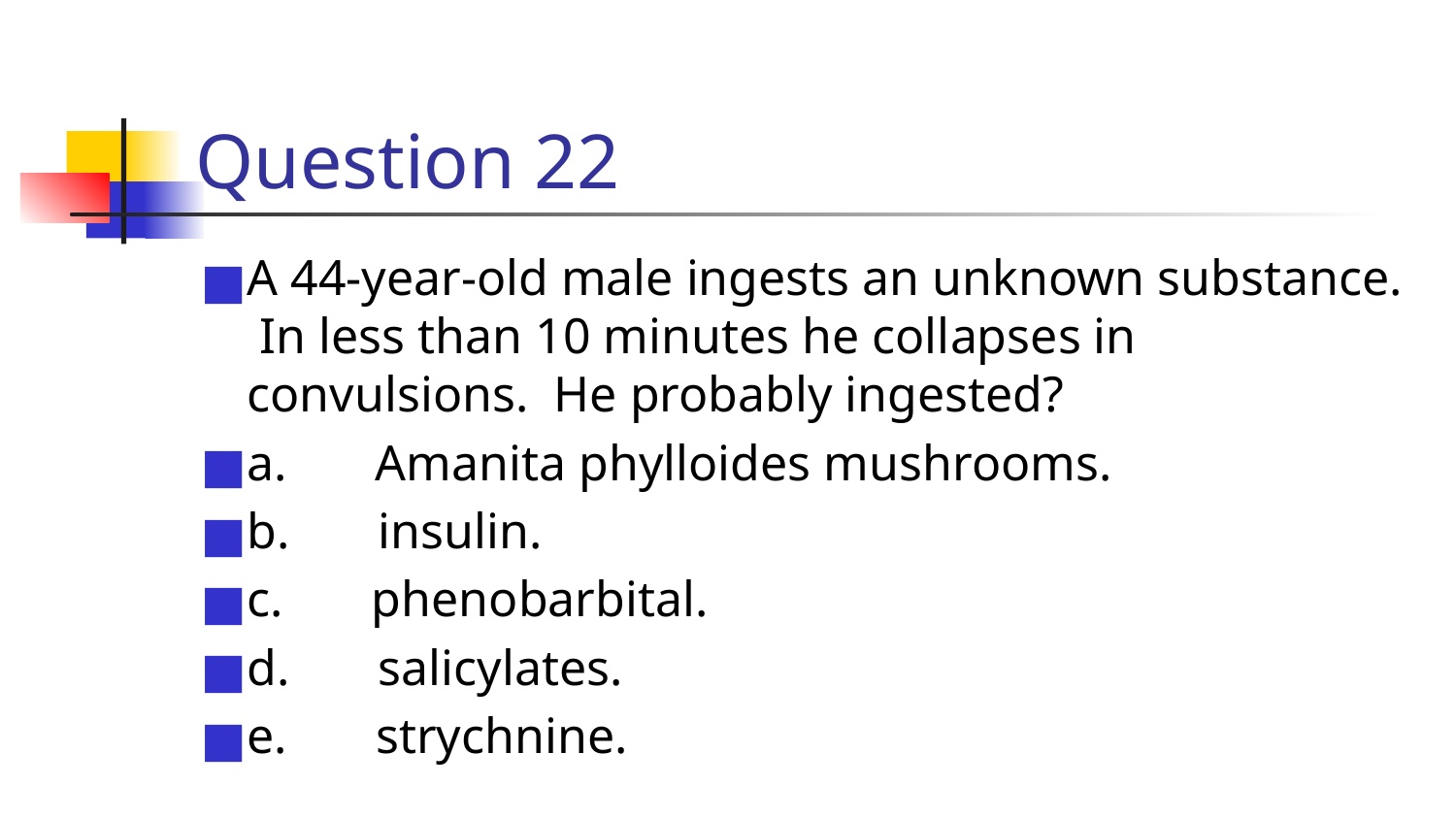

# Question 22
A 44-year-old male ingests an unknown substance. In less than 10 minutes he collapses in convulsions. He probably ingested?
a.       Amanita phylloides mushrooms.
b.       insulin.
c.       phenobarbital.
d.       salicylates.
e.       strychnine.

## Slide 49
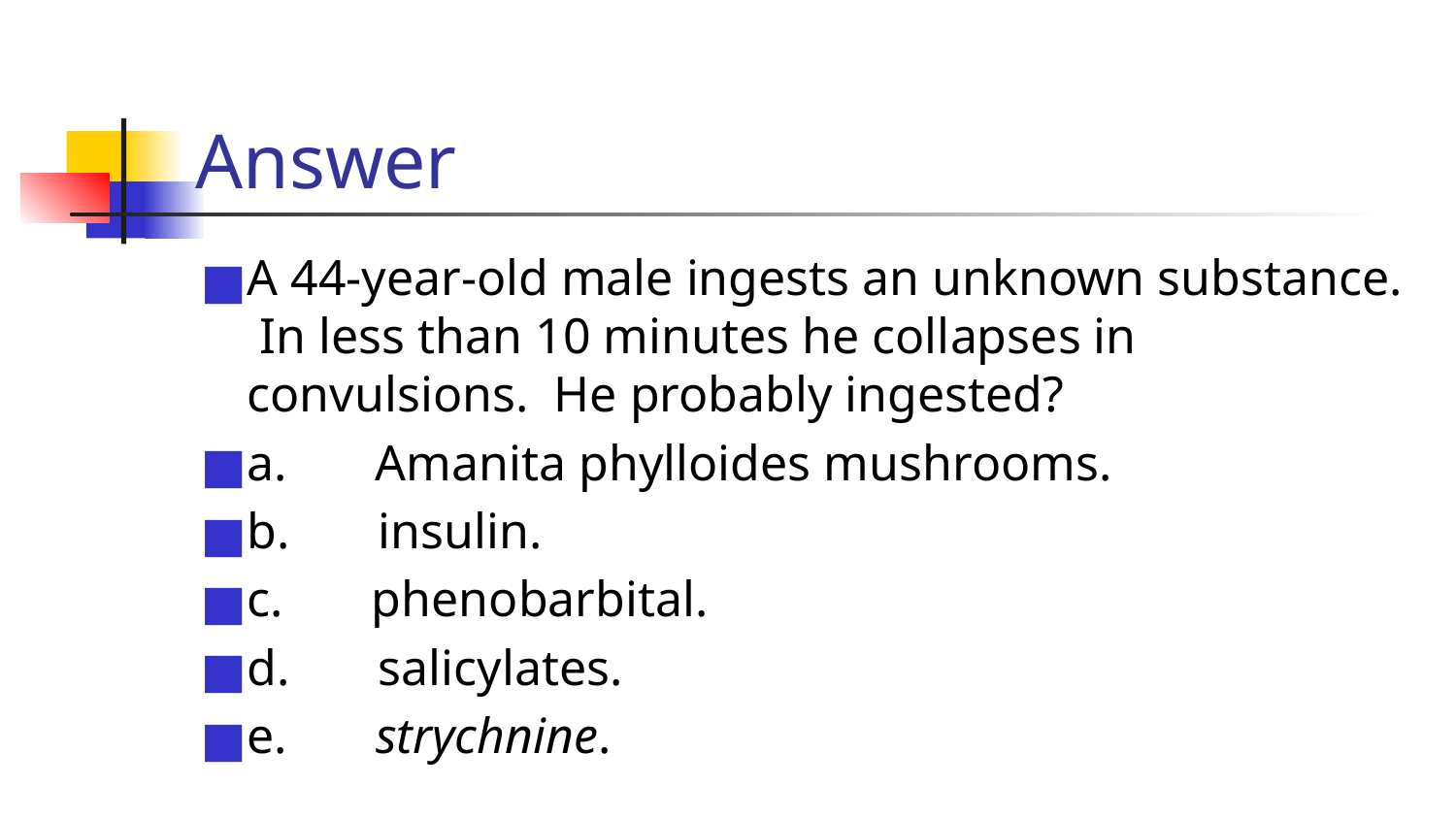

# Answer
A 44-year-old male ingests an unknown substance. In less than 10 minutes he collapses in convulsions. He probably ingested?
a.       Amanita phylloides mushrooms.
b.       insulin.
c.       phenobarbital.
d.       salicylates.
e.       strychnine.

## Slide 50
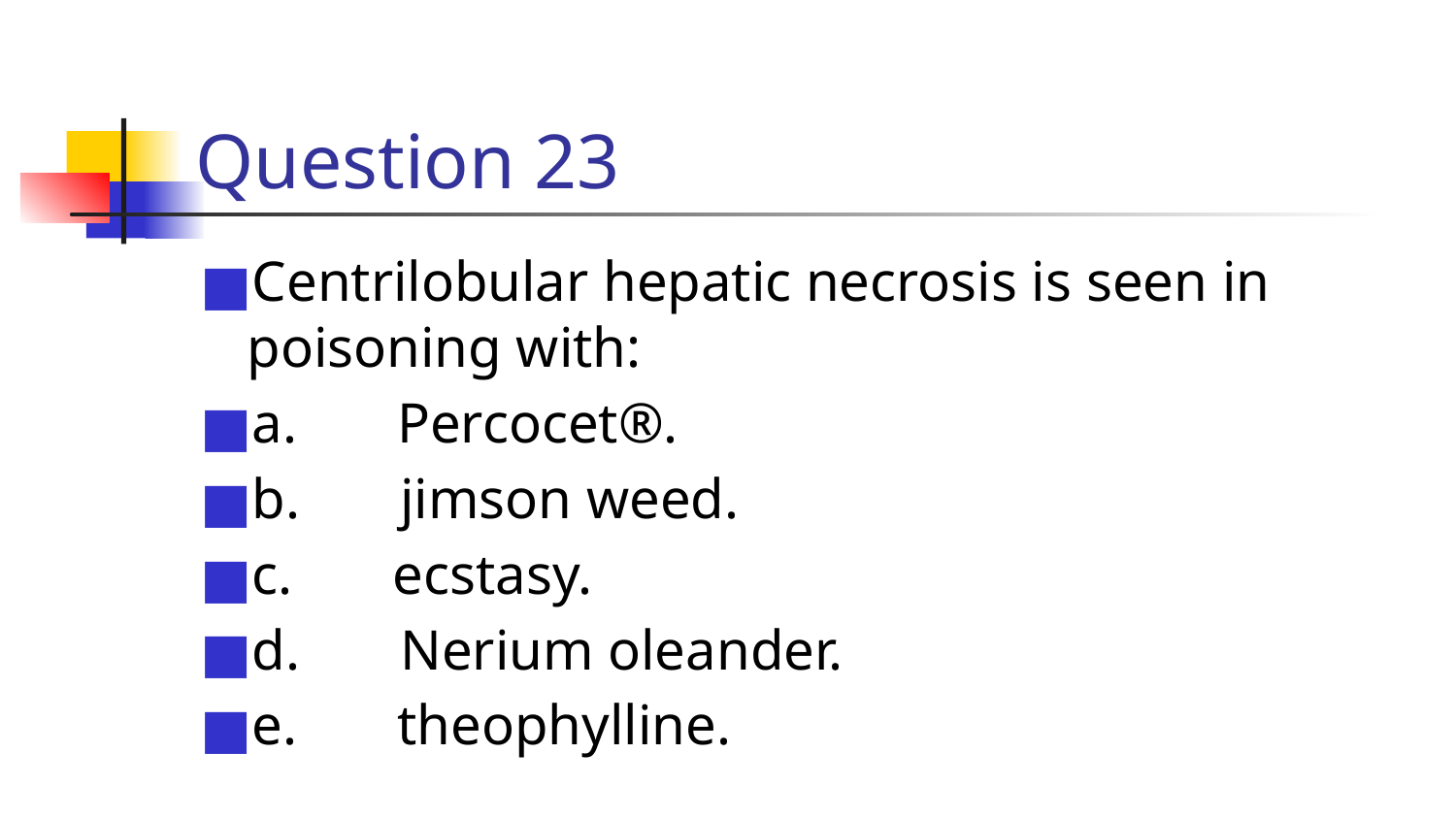

# Question 23
Centrilobular hepatic necrosis is seen in poisoning with:
a.       Percocet®.
b.       jimson weed.
c.       ecstasy.
d.       Nerium oleander.
e.       theophylline.

## Slide 51
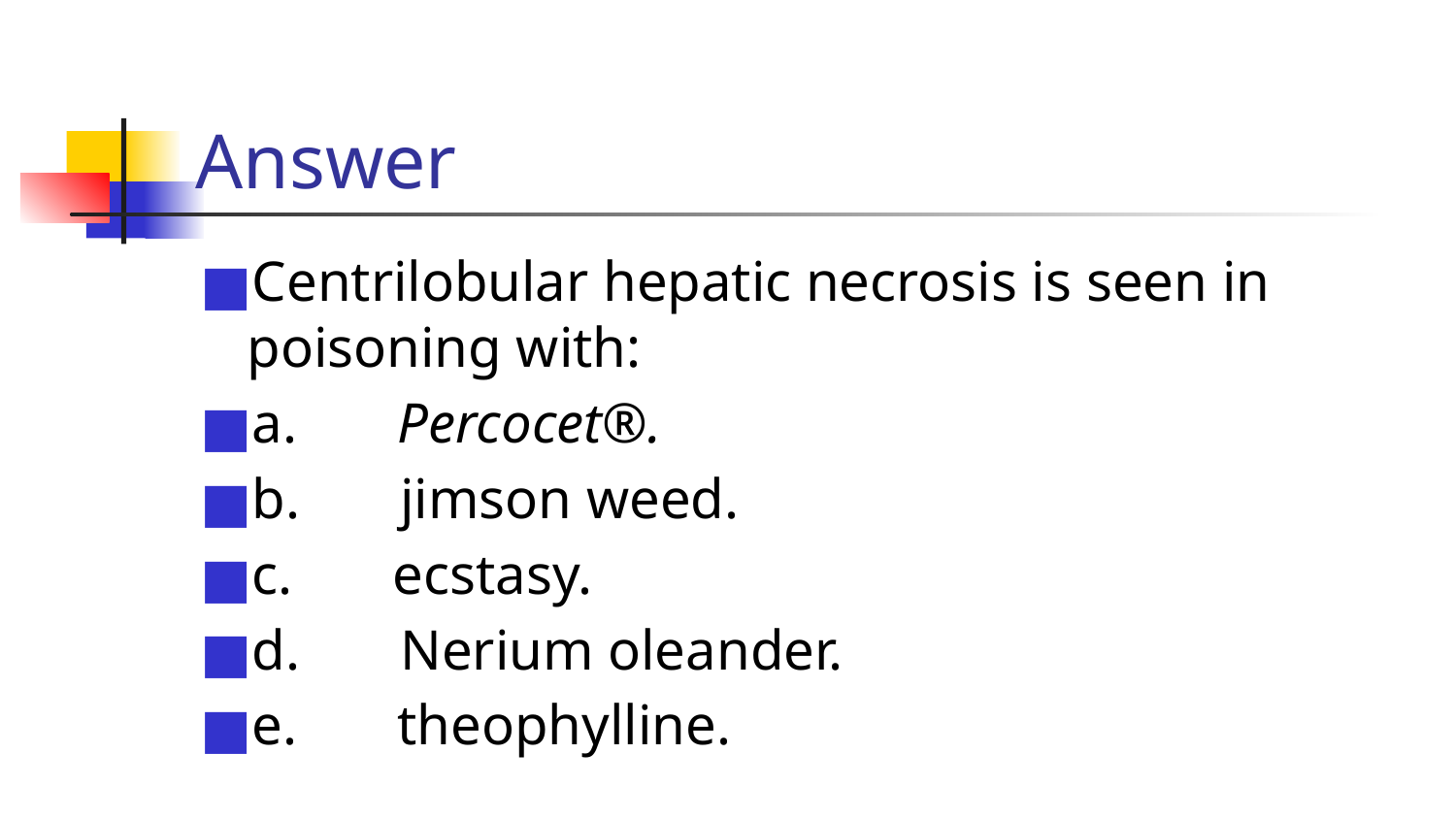

# Answer
Centrilobular hepatic necrosis is seen in poisoning with:
a.       Percocet®.
b.       jimson weed.
c.       ecstasy.
d.       Nerium oleander.
e.       theophylline.

## Slide 52
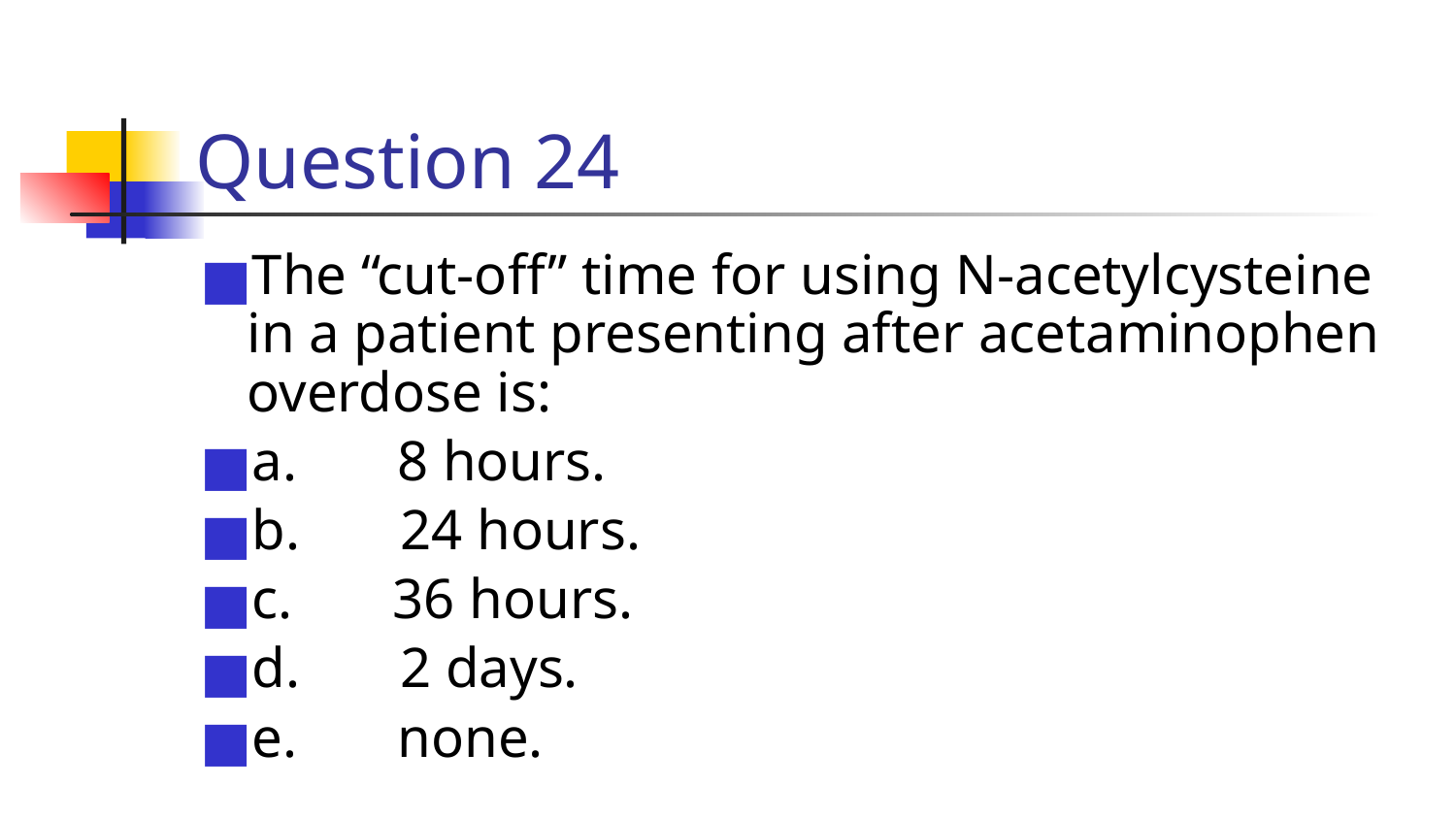

# Question 24
The “cut-off” time for using N-acetylcysteine in a patient presenting after acetaminophen overdose is:
a.       8 hours.
b.       24 hours.
c.       36 hours.
d.       2 days.
e.       none.

## Slide 53
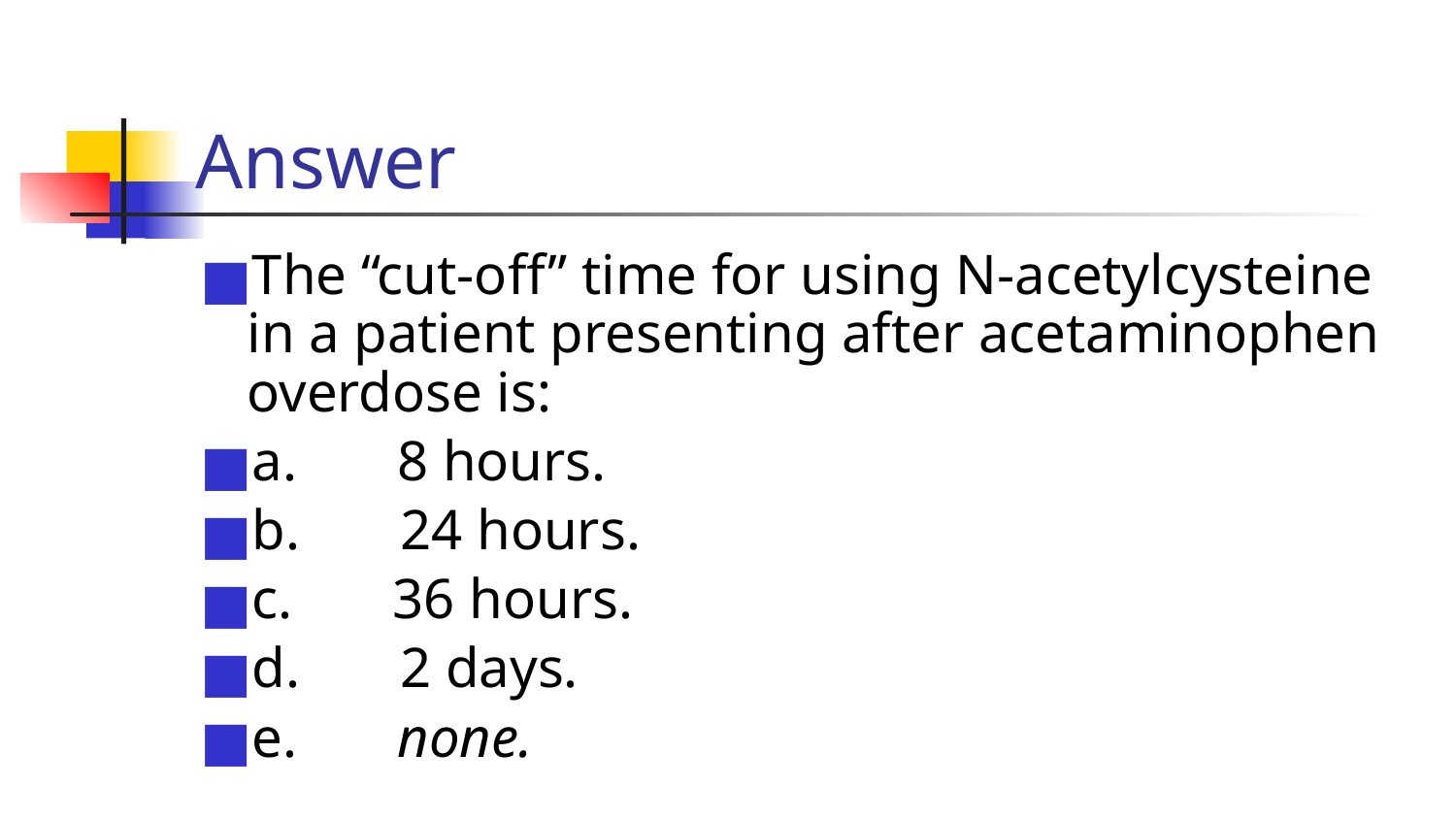

# Answer
The “cut-off” time for using N-acetylcysteine in a patient presenting after acetaminophen overdose is:
a.       8 hours.
b.       24 hours.
c.       36 hours.
d.       2 days.
e.       none.

## Slide 54
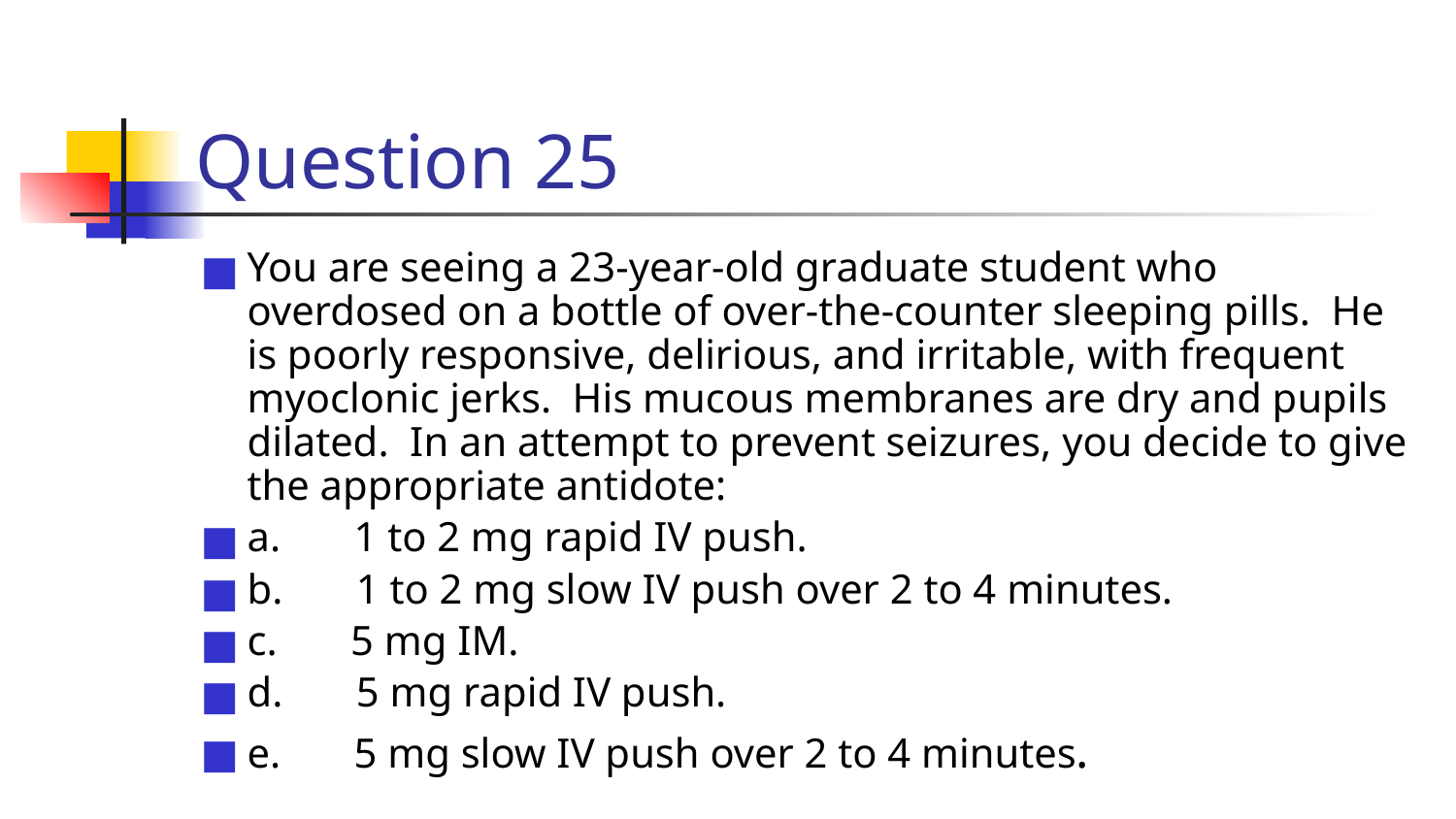

# Question 25
You are seeing a 23-year-old graduate student who overdosed on a bottle of over-the-counter sleeping pills. He is poorly responsive, delirious, and irritable, with frequent myoclonic jerks. His mucous membranes are dry and pupils dilated. In an attempt to prevent seizures, you decide to give the appropriate antidote:
a.       1 to 2 mg rapid IV push.
b.       1 to 2 mg slow IV push over 2 to 4 minutes.
c.       5 mg IM.
d.       5 mg rapid IV push.
e.       5 mg slow IV push over 2 to 4 minutes.

## Slide 55
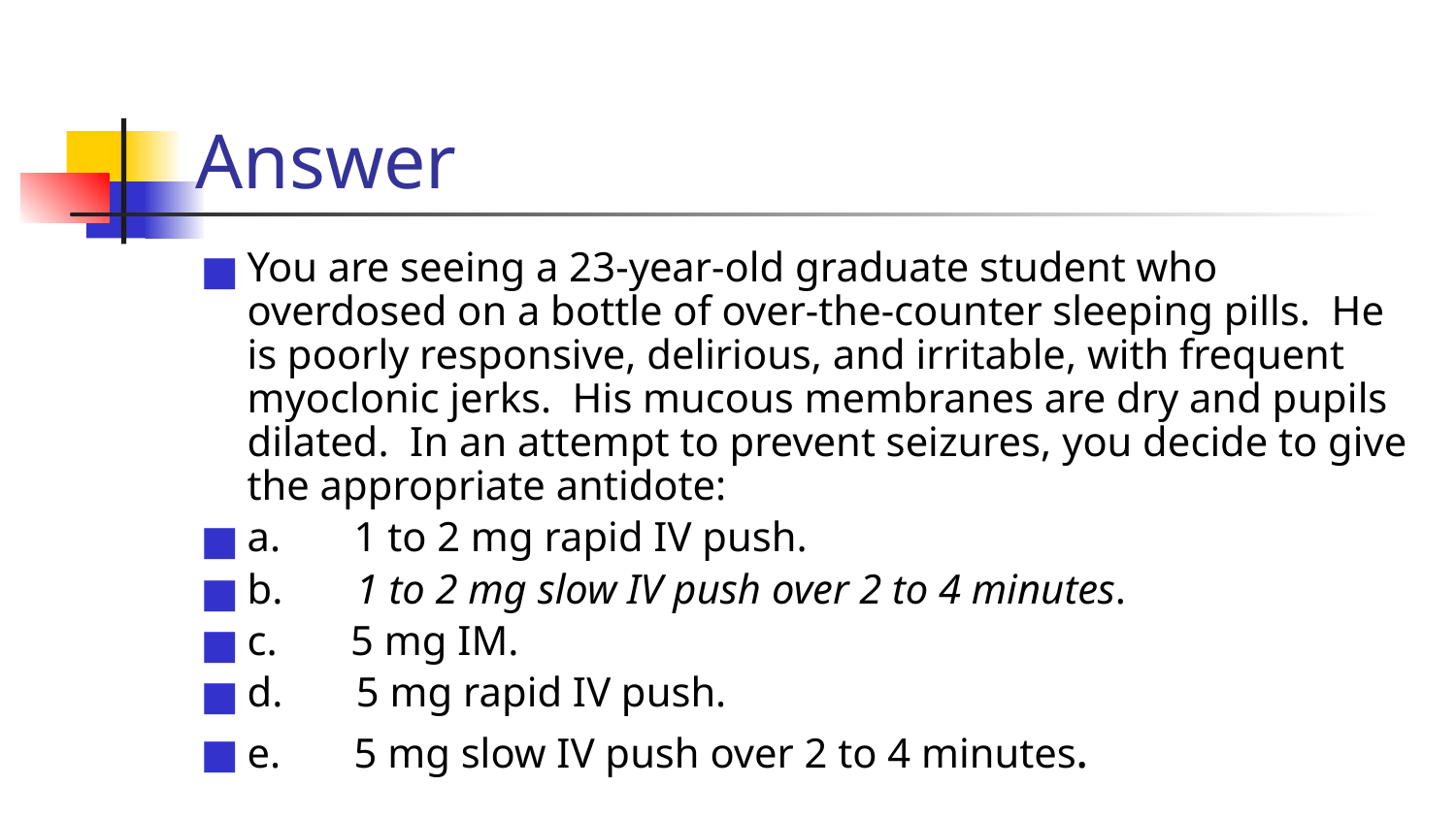

# Answer
You are seeing a 23-year-old graduate student who overdosed on a bottle of over-the-counter sleeping pills. He is poorly responsive, delirious, and irritable, with frequent myoclonic jerks. His mucous membranes are dry and pupils dilated. In an attempt to prevent seizures, you decide to give the appropriate antidote:
a.       1 to 2 mg rapid IV push.
b.       1 to 2 mg slow IV push over 2 to 4 minutes.
c.       5 mg IM.
d.       5 mg rapid IV push.
e.       5 mg slow IV push over 2 to 4 minutes.

## Slide 56
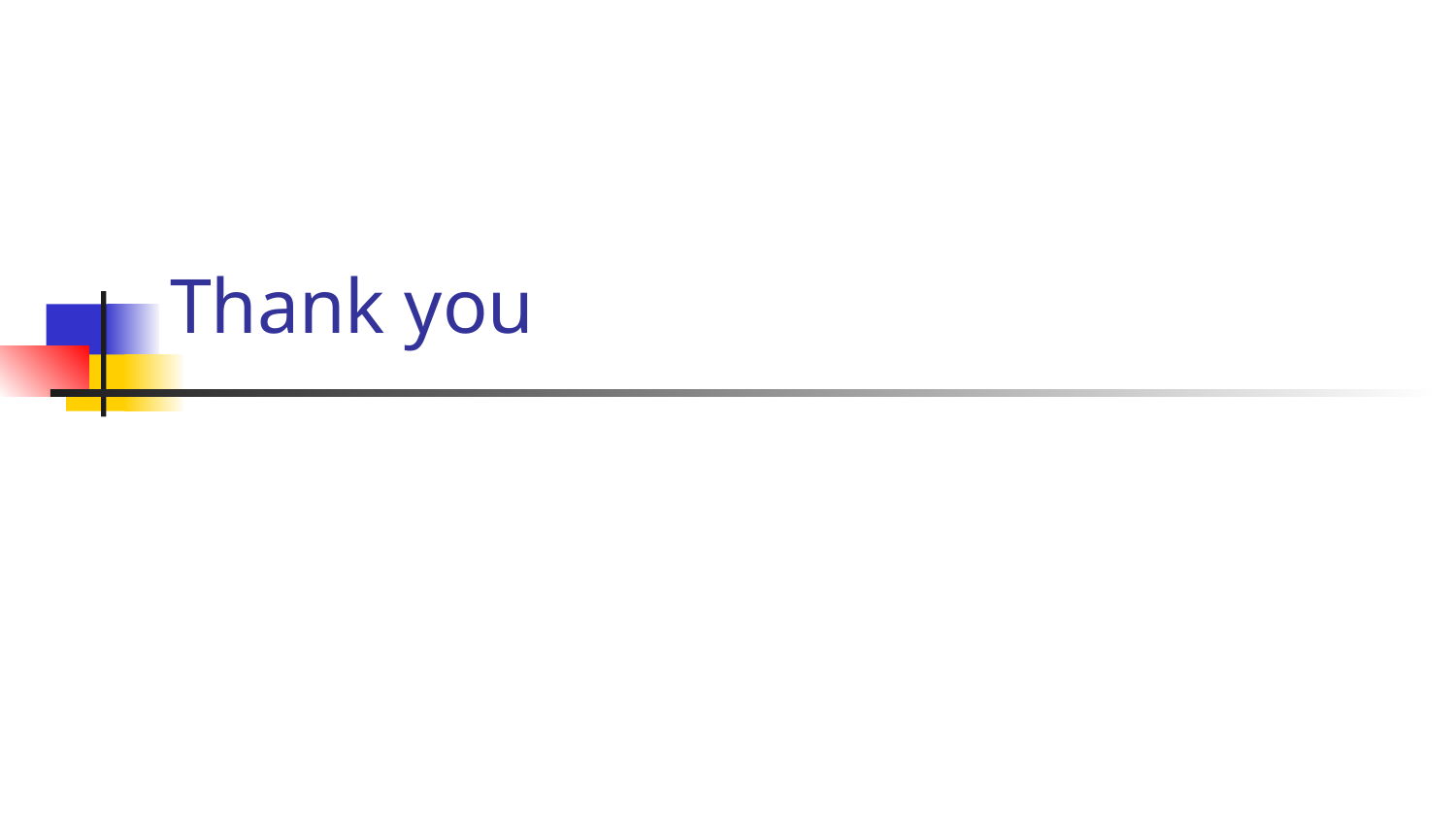

# Thank you
